# Oligodendrocyte differentiation alters tRNA modifications and codon optimality-mediated mRNA decay

**DOI:** 10.1038/s41467-022-32766-3

**Published:** 2022-08-25

**Authors:** Sophie Martin, Kevin C. Allan, Otis Pinkard, Thomas Sweet, Paul J. Tesar, Jeff Coller

**Affiliations:** 1 Department of Molecular Biology and Genetics, Johns Hopkins University School of Medicine, Baltimore, MD 21205 USA; 2 Department of Genetics and Genome Sciences, Case Western Reserve University School of Medicine, Cleveland, OH 44106 USA; 3 Center for Proteomics and Bioinformatics, Department of Nutrition, Case Western Reserve University School of Medicine, Cleveland, OH 44106 USA; 4 Department of Biology, Johns Hopkins University School of Medicine, Baltimore, MD 21205 USA

**Keywords:** RNA decay, tRNAs, Oligodendrocyte

## Abstract

Oligodendrocytes are specialized cells that confer neuronal myelination in the central nervous system. Leukodystrophies associated with oligodendrocyte deficits and hypomyelination are known to result when a number of tRNA metabolism genes are mutated. Thus, for unknown reasons, oligodendrocytes may be hypersensitive to perturbations in tRNA biology. In this study, we survey the tRNA transcriptome in the murine oligodendrocyte cell lineage and find that specific tRNAs are hypomodified in oligodendrocytes within or near the anticodon compared to oligodendrocyte progenitor cells (OPCs). This hypomodified state may be the result of differential expression of key modification enzymes during oligodendrocyte differentiation. Moreover, we observe a concomitant relationship between tRNA hypomodification and tRNA decoding potential; observing oligodendrocyte specific alterations in codon optimality-mediated mRNA decay and ribosome transit. Our results reveal that oligodendrocytes naturally maintain a delicate, hypersensitized tRNA/mRNA axis. We suggest this axis is a potential mediator of pathology in leukodystrophies and white matter disease when further insult to tRNA metabolism is introduced.

## Abstract (web summary)

Mutations in tRNA processing factors can lead to myelin disorders. This study shows that differentiated oligodendrocytes, cells that make the myelin, are characterized by different tRNA modifications and mRNA decay compared to their precursor cells.

## Introduction

Deciphering the genetic code involves transfer RNA (tRNA) selection by the ribosome to match a cognate codon displayed in the ribosomal A-site. Depending on functional tRNA concentrations; decoding speed, efficiency and accuracy for each codon varies. Moreover, codon context can influence decoding in similar ways. Thus, for many reasons, some codons/tRNA combinations are more optimal relative to others. This concept is termed c*odon optimality* which is defined broadly as the non-uniform decoding rate, determined (in part) by the functional concentration of tRNAs^1–3^.

Transfer RNA functionality not only impacts the rate by which an mRNA is read by the ribosome, but it also is a major determinant of the mRNA’s stability. First shown in budding yeast, *Codon Optimality-Mediated mRNA decay* (CO-MD) is now recognized as a general mechanism to regulate transcript stability, contributing broadly to the range of eukaryotic mRNA half-lives^4–8^. Key is that minute decoding hesitations induced by subtle difference in tRNA levels will result in a transient ribosome conformation^9–11^. This transitory state is recognized by mRNA degradation machinery; thus, each hesitation increases the probabilistic chance of degrading the mRNA transcript. The cumulative codon composition sets the transcript’s overall decay rate with transcripts enriched in codons that are translated efficiently (optimal) being more stable than mRNAs enriched in non-optimal codons. In this context, tRNAs themselves become master arbiters of both mRNA expression and stability.

Since the ribosome/tRNA axis mediates the overall rate of translational elongation and mRNA stability, perturbations to tRNA levels could powerfully influence overall gene expression. In a simple organism like yeast, tRNA pools are relatively static under normal conditions. Metazoans, however, are hallmarked by specialized cell types. Thus, tRNA pools might exhibit plasticity between cell types and tissues^12–14^. Indeed, proliferative and differentiated cells can vary tRNA expression and this is well matched to the cell specific mRNA codon content in some cases^15–17^.

Importantly, however, tRNA levels are not the only feature that should alter ribosome decoding potential. Translational elongation requires a fully functionalized tRNA in order to proceed normally. And the genesis of tRNAs is complex involving RNA processing, splicing (for some), 3′ end CCA addition, amino acid charging, and extensive RNA modification at numerous positions (including the anticodon loop; ACL)^18–20^. Thus, changes to any of these events can contribute to the “functional” concentration of cellular tRNAs and alter ribosome decoding efficiency.

In particular, tRNA nucleotide composition is heavily post-transcriptionally altered, with over 200 unique modifications occurring within tRNA transcripts. Many of these unique nucleotide modifications are required for tRNA stability, folding, and appropriate amino acid charging. An important class of modifications occur at or near the anticodon thereby affecting decoding efficiency and accuracy by the ribosome^21–24^. Like tRNA expression, tRNAs modifications can vary between tissues, during stress, cancer, or during cellular differentiation^13,25–29^. Of interest is when these programed changes in tRNA decoding potential might hypersensitize cells to disease susceptibility.

Leukodystrophies are rare progressive genetic disorders affecting the Central Nervous System (CNS) white matter resulting in neurodegeneration. A defined class of leukodystrophies result from aberrant myelin production^30,31^. Oligodendrocyte Progenitor Cells (OPCs) are proliferative glial cells that mature into myelin-producing oligodendrocytes during CNS development^32^. Oligodendrocytes generate myelin as an extension of their plasma membrane which wraps around neuronal axons, insulating and allowing for proper action potential conduction^33,34^. Loss of oligodendrocyte function (i.e., myelin production or maintenance) is highly debilitating, resulting ultimately in premature death. Critically, a large cohort of patients with hypomyelinating leukodystrophies have mutations mapping to factors implicated in the ribosome/tRNA axis:^35^ for example, mutations in the translation factor eIF2B cause Vanishing White Matter disease^36^, and mutations in RNA polymerase III subunits POLR3A and POLR3B, required for tRNA transcription, cause 4H Leukodystrophy or POLR3-Related Leukodystrophy, respectively^37–41^. Mutations in numerous aminoacyl-tRNA synthetases (ARSs) are also linked to hypomyelination, such as in HBSL (Hypomyelination with Brainstem and Spinal cord involvement and Leg spasticity, DARS related disorder) or HLD9 (Hypomyelinating Leukodystrophy 9, RARS related disorder)^42–46^. However, the mechanism governing this hypersensitivity of oligodendrocytes to perturbations in tRNA metabolism remains unknown.

In this study, we focus on the tRNA/mRNA interrelationship in oligodendrocytes in an attempt to understand the underlying hypersensitivity of these cells to perturbations in tRNA metabolism. Using a combination of approaches, we determine tRNA levels and their modification status in murine OPCs and oligodendrocytes. We observe key differences in the metabolism of certain tRNAs that may alter their function in oligodendrocytes vs. their progenitor cells. In particular, we observe several tRNAs are hypomodified in oligodendrocytes, impacting their decoding potential and concomitantly impacting CO-MD in these cells. Our results highlight specific characteristics of tRNA biology and its relationship to the post-transcriptional control of mRNA within oligodendrocytes. The observed hypersensitized ribosome/tRNA state provides some insight into the molecular underpinning that might result in leukodystrophy etiology and pathology whenever additional mutations occur to the translational apparatus.

## Results

### Differential tRNA expression in oligodendrocytes and their progenitors

For the most part, tRNA concentrations seem relatively constant between distinct tissues (Pinkard et al. 2020). Importantly, however, tRNA modifications^25^, tRNA abundance^16,17^ and/or tRNA aminoacylation^17,47^ can differ between distinct cellular proliferation states especially in the CNS^13,48^.

**Fig. 1:**
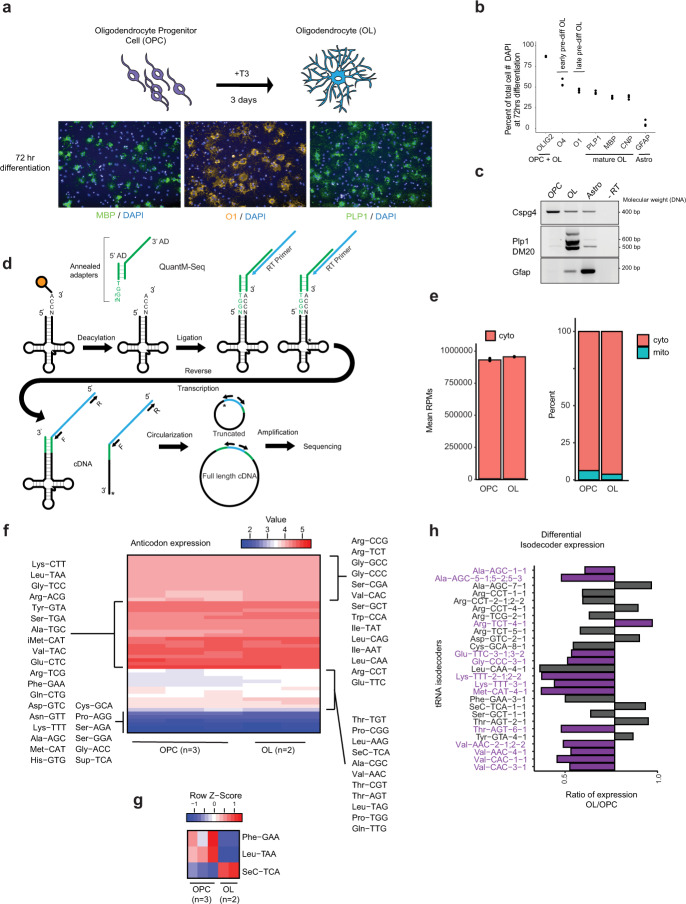
Differential tRNA expression in oligodendrocytes and their progenitors. **a** Model of differentiation of OPCs to oligodendrocytes (OL). At 3 days after addition of thyroid hormone T3, markers of mature oligodendrocytes are detected by immunocytochemistry. **b** Immunofluorescence quantitation after 3 days of differentiation. Percentage of cells that express the oligodendrocytes markers MBP, PLP1, CNP (mature oligodendrocytes), O1 (late pre-differentiating oligodendrocytes), O4 (early pre-differentiating oligodendrocytes), the oligodendrocyte lineage marker OLIG2 (OPCs and oligodendrocytes), or the astrocytes marker GFAP. A total number of cells n of 2341 for OLIG2, 2587 for O4 and GFAP, 5865 for O1 and MBP, and 2491 for PLP1 and CNP, were examined over 3 replicate wells (8 fields per replicate). **c** Detection of *Cspg4*, *Plp1* and *Gfap* mRNAs, respective markers of OPCs, oligodendrocytes (OL) and astrocytes (Astro), by RT-PCR. *-RT: control without Reverse Transcriptase*. **d** Outline of the QuantM-seq, that relies on the ligation of a double-stranded adapter complementary to the 5′ and 3′ termini of mature tRNAs, and circularization of the cDNA before library preparation. **e** Mean cytosolic tRNA reads per million (RPMs) (bar chart, left) and percentage of reads that correspond to cytoplasmic or mitochondrial tRNAs (stacked bar chart, right) in OPCs and oligodendrocytes (OLs). The values of *n* = 3 independent experiments for OPCs, and *n* = 2 independent experiments for oligodendrocytes, are superposed to the means. **f** Heatmap of tRNA reads collapsed by anticodon groups. *n* = number of biological replicates. **g** Row *Z*-score heatmap of expression for tRNAs that significantly change between OPCs and oligodendrocytes (adjusted *p* value < 0.01 from DESeq2 analysis, Wald test with Benjamini–Hochberg correction). *n* = number of biological replicates. **h** Expression of tRNA isodecoders that are significantly different between OPCs and oligodendrocytes (OL), represented as a log2 ratio OL/OPC (adjusted *p* value < 0.01 from DESeq2 analysis, Wald test with Benjamini–Hochberg correction).

Given that numerous mutations in tRNA biogenesis enzymes lead to defects in myelin levels, we investigated tRNA expression and aminoacylation levels in OPCs and oligodendrocytes. We used a well-established method to generate OPCs from mouse pluripotent stem cells and differentiated them into oligodendrocytes in vitro^49–52^ (Fig. 1a and Supplementary Fig. 1a). We confirmed the expression of oligodendrocytes markers including the cell surface marker O1 and the myelin membrane proteins, myelin basic protein (MBP) and proteolipid protein 1 (PLP1), at 3 days after addition of thyroid hormone (T3) (Fig. 1a, b). Differentiation results in the majority of cells forming oligodendrocytes (mature or pre-differentiated), while a population remains as undifferentiated OPCs, as quantified by immunocytochemistry and transcript expression of OPC and oligodendrocyte specific markers (Fig. 1b, c).

We measured tRNA levels in OPCs and oligodendrocytes using QuantM-seq, which allowed robust quantitation of tRNAs in different cell lines and mouse tissues^5,13^ (Fig. 1d). The contribution of cytoplasmic and mitochondrial tRNA genes to the total number of reads was similar in OPCs and oligodendrocytes, with more than 90% being cytoplasmic in both cell types, although the mitochondrial pool was higher in OPCs than oligodendrocytes (Fig. 1e). Interestingly, when grouped by anticodon pools, most tRNAs are expressed at similar levels between OPCs and oligodendrocytes (Fig. 1f), similar to what was observed between mouse tissues^13^. However, three tRNAs showed a significant difference in expression between the two cell types at the anticodon level (Fig. 1g, adjusted *p* value 0.01). Phe-GAA and Leu-UAA isoacceptor classes were less expressed in oligodendrocytes while SeC-UCA tRNA was more expressed in oligodendrocytes. Importantly, while there are a few differences in tRNA anticodon pools between OPCs and oligodendrocytes, the expression of numerous isodecoders (different tRNAs with same anticodon) changed between the two cell types (Fig. 1h). Interestingly, half of the isodecoders differentially expressed between OPCs and oligodendrocytes are enriched in the CNS compared to non-CNS tissues (Fig. 1h; in purple^13^,), suggesting a cell-type specific regulation of expression within the CNS that might be important for function in oligodendrocytes. Thus, by QuantM-seq, total tRNA anticodon pools are relatively constant but isodecoder expression fluctuates between cell types within the oligodendrocyte lineage.

### tRNA charging status between OPCs and oligodendrocytes

Numerous leukodystrophies are linked to mutations in tRNA synthetases^43,44,53^. Appropriate aminoacylation of a tRNA will impact their decoding potential. Thus, in addition to evaluating tRNA levels by QuantM-seq, we also evaluated the aminoacylation status of each tRNA class using acid-urea PAGE followed by Northern analysis. This allows for the electrophoretic separation of different forms of a tRNA due to changes in bulk, charge, and/or conformation, notably depending on aminoacylation^54^. For this analysis, we included astrocytes, which were derived from the same OPCs, as a control to separate differentiation processes from oligodendrocyte-specific biology (Supplementary Fig. 1b). We observed that most tRNAs are fully aminoacylated in all cell types (Supplementary Fig. 2), with the exception of Cys-GCA which shows more deacylated species in oligodendrocytes. These results suggest that most tRNAs are aminoacylated to the same extent between different glial populations. While the Cys-GCA tRNA was less aminoacylated in oligodendrocytes, this phenomenon will not be the focus of this study.

### tRNA sequencing distinctions between OPCs and oligodendrocytes

**Fig. 2:**
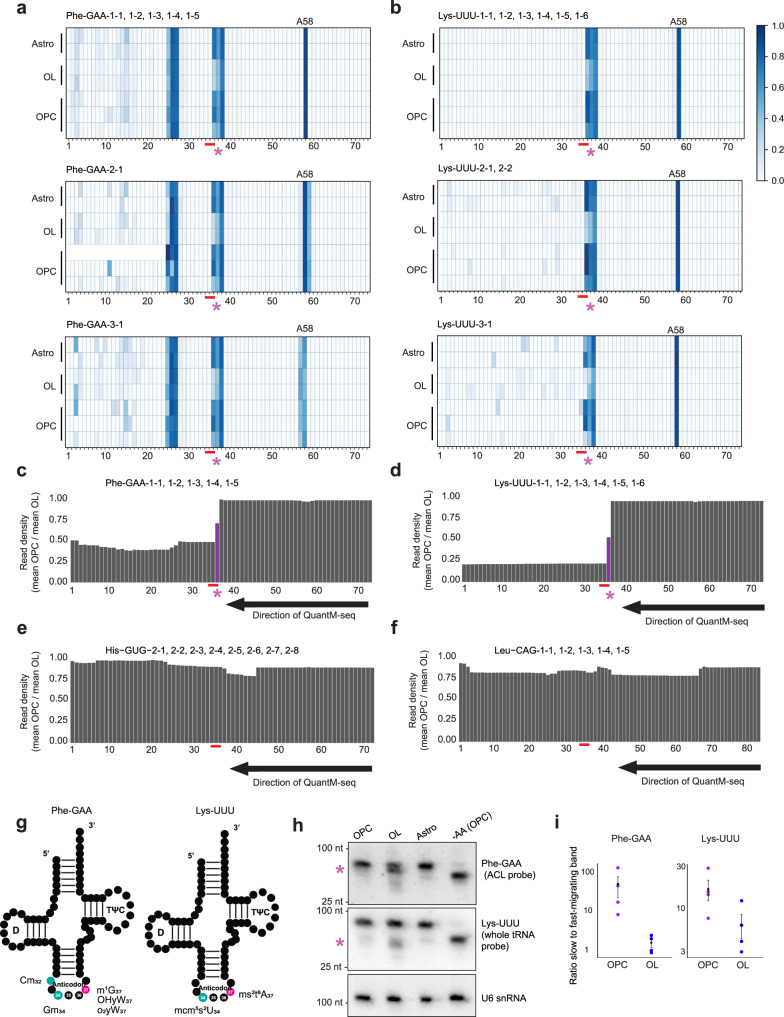
tRNA sequencing distinctions between OPCs and oligodendrocytes. **a–b** Heatmaps for the different isodecoders of Phe-GAA (**a**) and Lys-UUU (**b**), representing sequencing variant fractions at each tRNA position (*x*-axis) across OPCs (3 biological replicates), oligodendrocytes (OL, 2 biological replicates) and astrocytes (Astro, 2 biological replicates) (*y-*axis). The numbers below the plots indicate nucleotide position, the red line shows the anticodon position (nucleotides 34–36). The color bar indicates the color scale of the variant frequency. DEXSeq differential analysis with Benjamini–Hochberg correction between oligodendrocytes and OPCs/astrocytes at position 37 of Phe-GAA and Lys-UUU: adjusted *p* value < 0.02. **c–d** Bar charts of the ratio of reads density in OPCs vs. oligodendrocytes (OL), at each nucleotide position of Phe-GAA (isodecoder 1, in **c**) and Lys-UUU (isodecoder 1, in **d**) tRNAs. **e–f** Same as (**c–d**), for His-GUG (isodecoder 2, in **e**) and Leu-CAG (isodecoder 1, in **f**), showing the same density OPC vs. oligodendrocytes (OL) at each nucleotide. **g** Two-dimensional representation of Phe-GAA tRNA and Lys-UUU tRNA, with the modified nucleosides at position 32, 34 and 37 in the anticodon loop highlighted in color. **h** Northern blot analysis of Phe-GAA and Lys-UUU tRNAs. Probes complementary to the anticodon loop (ACL) or full-length tRNA (whole tRNA) sequence were used. U6 snRNA is used as a loading control. *-AA (Amino Acid)*: tRNAs were deacylated by alkaline hydrolysis. Asterisk: tRNA species with higher electrophoretic mobility in OL. nt: nucleotides, molecular weight estimated from bromophenol blue and xylene cyanol dyes signal. **i** Quantitation of the signal intensity of the two tRNA species detected by Northern blot after acid urea PAGE, for Phe-GAA and Lys-UUU tRNAs. Plotted are the values of four biological replicates in OPCs (purple) and oligodendrocytes (OL, blue), with the mean ± SEM (error bars) in black.

tRNAs are heavily modified with unique nucleotide analogs. These modifications are required for tRNA function and can change under various cellular conditions^21,25,26^. To evaluate possible changes in tRNA modifications that might occur during oligodendrocyte differentiation, we analyzed sequence variances observed by QuantM-seq compared to the reference genome for each individual isodecoder. This analysis takes advantage of reduced reverse transcriptase (RT) fidelity in response to a non-canonical modified nucleotide. In addition, this approach allows for read length distinctions to be monitored in response to abortive transcription, which can be the result of some modified nucleotides. We and others have previously used this approach as a means to approximate the position and level of select tRNA base modifications^13,23,55^. For example, we can see a misincorporation by the RT in the TψC-loop (nucleotide position 58) of most of tRNAs known to be modified with N^1^-methyladenosine (m1A; Fig. 2a, b). For most tRNAs, we observed few distinctions between OPCs, oligodendrocytes and astrocytes; sequence variation and read length observed by QuantM-seq was uniform between cell types. This was true except for four specific tRNA classes which showed a difference in sequence identity and read length by QuantM-Seq in oligodendrocytes when compared to either OPCs or astrocytes. Specifically, all isodecoders for Phe-GAA tRNA and all isodecoders for the Lys-UUU tRNA exhibited unique read signatures in the ACL (asterisk, nucleotides 36–38) in OPCs and astrocytes that are diminished in oligodendrocytes (Fig. 2a, b). Moreover, most of the isoacceptors and isodecoders for the Ala tRNAs family exhibited unique D-loop read signature in OPCs and astrocytes that are decreased in oligodendrocytes (Supplementary Fig. 3a). Lastly, a read signature in the Arg-UCU isodecoder family was seen in the ACL, that had a modest decrease in oligodendrocytes compared to OPCs and astrocytes (Supplementary Fig. 3b). Importantly, the nature of QuantM-seq requires a mature tRNA, thus read length distinctions are not the result of tRNA fragmentation, rather are the result of abortive transcription in response to a modification^13^. In each case, the fidelity of read identity and length in oligodendrocytes is suggestive of a hypomodified state to these tRNAs that is specific to the cell type.

Phe-GAA and Lys-UUU tRNAs showed the highest degree of variation between OPCs/astrocytes and oligodendrocytes (significant from DEXSeq analysis with adjusted *p* value < 0.02^13^). Analysis of the reads density at each position confirmed a drop of reads in 5′ of these tRNAs, starting at nucleotide 37 in the ACL, suggestive of reverse transcription being impeded by a modification. However, there were more full-length reads in oligodendrocytes, with a twofold decrease in the ratio of reads OPC/oligodendrocyte from the anticodon position for tRNA Phe-GAA (Fig. 2c), and a 4-fold decrease for Lys-UUU (Fig. 2d), which supports the notion of a hypomodified state for these tRNAs in oligodendrocytes. The ratio of reads OPC/ oligodendrocyte for other tRNAs such as His-GUG (Fig. 2e) or Leu-CAG (Fig. 2f) tRNAs is close to 1 along the whole tRNA sequence, suggesting hypomodification -for modifications that impact the RT fidelity and can be detected by QuantM-seq- is specific to select tRNAs in oligodendrocytes (Supplementary Fig. 3c, d).

### Phe-GAA and Lys-UUU tRNA are hypomodified in oligodendrocytes

Phe-GAA and Lys-UUU tRNAs are heavily modified in their ACL^56^. Phe-GAA carries a 2′-O-methylguanosine (Gm) at the wobble position (nucleotide 34). At position 37, Phe-GAA bears 1-methylguanosine (m^1^G) which can be further modified to the complex nucleoside wybutosine (yW; Fig. 2g). The yW modification is unique to phenylalanine tRNA and enhances the stability of the ACL in the ribosome A-site upon tRNA accommodation^57^. Wybutosine synthesis is complex and in mammals it is further hydroxylated leading to the formation of the derivatives hydroxywybutosine (OHyW) and peroxywybutosine (o2yW)^20,58^.

In Lys-UUU tRNA, the wobble uridine U_34_ is modified to 5-methoxycarbonylmethyluridine (mcm^5^U) and further thiolated to mcm^5^s^2^U (5-methylcarboxymethyl-2-thiouridine)^59^. The nucleotide adjacent to the anticodon is also modified to ms^2^t^6^A (2-methylthio-N6-threonylcarbamoyladenosine), and both U_34_ and A_37_ modifications are suggested to be important in decoding potential^60^ (Fig. 2g).

To validate the use of QuantM-seq and analysis of sequencing variants as a proxy for select modification differences between cell types, we performed QuantM-seq on budding yeast RNA from WT and *TYW1* deletion (*tyw1∆)*. Tyw1 is an enzyme involved in the biosynthesis of wybutosine at G_37_ of Phe-GAA tRNA, both in yeast and mouse. The unique read signature observed at position 37-38 of Phe-GAA in WT, due to RT stall, is strongly diminished in *tyw1∆*, and gradually diminished in samples containing different ratios of RNA from WT and *tyw1∆* (Supplementary Fig. 4a). This decrease is linear (Supplementary Fig. 4b), validating the use of our first approach to study differences in the modification of Phe-GAA tRNA.

However, in an effort to validate and investigate the modification status of tRNA Phe-GAA and Lys-UUU in more detail, we performed acid urea PAGE/Northern blotting. This revealed a striking mobility pattern that is specific to oligodendrocytes for both tRNAs (Fig. 2h). In OPCs and astrocytes, a single predominant tRNA species is detected, while in oligodendrocytes a second faster migrating species is also observed (Fig. 2h, marked by asterisk). Quantitation of the signal intensity from the two Phe-GAA species shows a significant difference between OPCs and oligodendrocytes, as the ratio of the two bands (slow:fast) is more than 50:1 in OPCs while it is about 2:1 in oligodendrocytes (Fig. 2i). Similarly, for the Lys-UUU tRNA, the ratio is about 20:1 in OPCs but 6:1 in oligodendrocytes (Fig. 2i). In both cases, the faster migrating species do not co-migrate with the deacylated control, thus these unique species do not represent uncharged tRNA. Further, the mobility distinctions observed for Phe-GAA and Lys-UUU are distinct. As shown below, the mobility pattern observed for Phe-GAA is due to hypomodification of this tRNA. Although different from Phe-GAA tRNA, we hypothesize that the mobility pattern observed for Lys-UUU tRNA may also be the result of hypomodification for this tRNA in oligodendrocytes (see below). Finally, quantification of the signal from Phe-GAA from three different northern blots confirms the decrease of the levels of this tRNA in oligodendrocytes that was observed by QuantM-seq (Supplementary Fig. 5a).

### The Phe-GAA tRNA is hypomodified in oligodendrocytes

Looking at each of the two tRNAs (Phe-GAA and Lys-UUU tRNA) of interest, we chose to analyze them in isolation. First, focusing in on Phe-GAA tRNA, we hypothesized that the observed electrophoretic mobility distinctions are the result of differential G_37_ modification. Wybutosine derivatives are tricyclic nucleosides characterized by large hydrophobic side chains. However, wybutosine intermediates with hypo-modified lateral groups have been isolated in various tumor cells. These differently modified groups on yW precursors are known to affect their chromatographic mobility and hydrophobic properties^61–64^. They could alter their ionic charge under acidic gel electrophoresis conditions, thereby inducing a mobility shift^54^. Importantly, however, we had to rule out other possibilities.

**Fig. 3:**
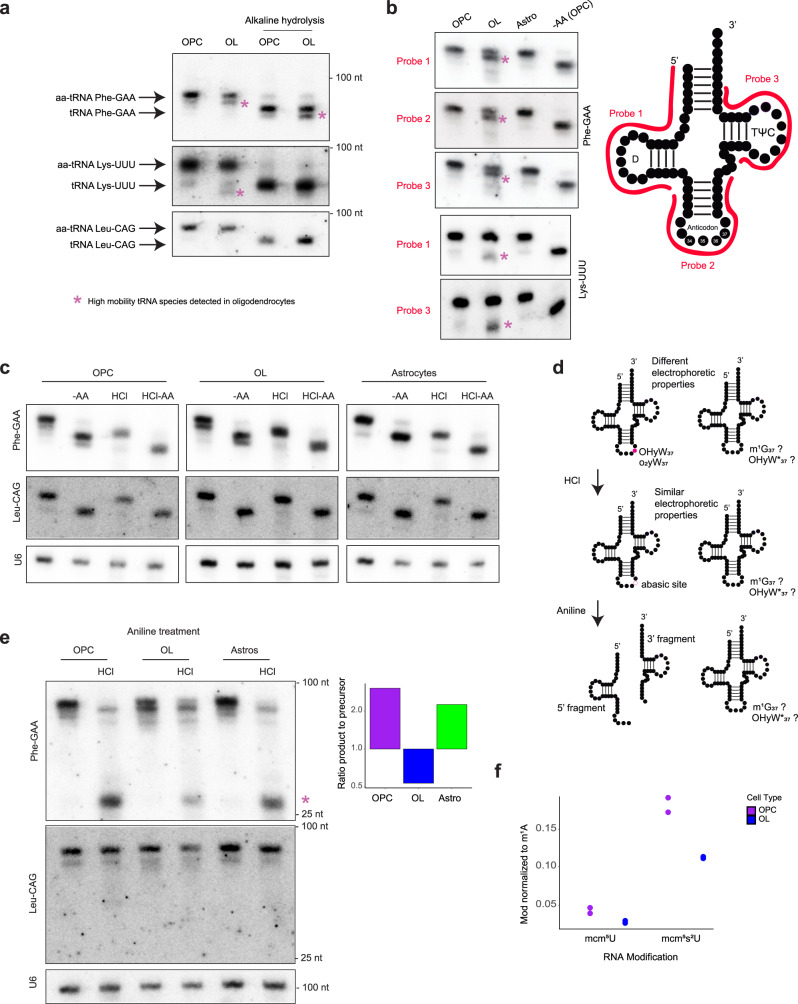
Phe-GAA and Lys-UUU modifications in the ACL differ between OPCs and oligodendrocytes. **a** Acid-urea PAGE and Northern blot analysis for Phe-GAA, Lys-UUU and Leu-CAG in OPCs and oligodendrocytes (OL), charged with amino acid (AA) or uncharged (alkaline hydrolysis). nt: nucleotides, molecular weight estimated from bromophenol blue and xylene cyanol signals. **b** Acid-urea PAGE and Northern blot analysis of Phe-GAA and Lys-UUU, probed with different oligonucleotides covering the D-loop, ACL or TψC-loop (-AA = minus amino acid, deacylation induced by alkaline hydrolysis). On the right is a two-dimensional representation of the tRNA. **c** Acid-urea PAGE and Northern blot analysis of OPC, oligodendrocytes (OL) and astrocytes (Astro) RNA submitted to alkaline hydrolysis (-AA, deacylation), acidic hydrolysis (HCl, depurination of wybutine) or acidic followed by alkaline hydrolysis (HCl -AA). Phe-GAA was detected with a full-length tRNA complementary probe. U6 snRNA is used as a loading control. **d** Two-dimensional representation of Phe-GAA tRNA modified with wybutosine (yW) derivatives (left), or hypomodified (right). Schematic of the consequences of acidic hydrolysis leading to the formation of an abasic site of the yW modified tRNA, and aniline treatment leading to chain scission at the abasic site. Phe-GAA tRNA that would not present the fully modified yW or derivatives (right) would not be sensitive to treatment. Modifications at G_37_: OHyW_37_, hydroxywybutosine; o_2_yW_37_, peroxywybutosine, m¹G_37_, 1-methylguanosine; OHyW*_37_, hypomodified hydroxywybutosine. **e** Acid-urea PAGE and Northern blot analysis of OPC, oligodendrocytes (OL) and astrocytes (Astro) RNA submitted to acidic hydrolysis (HCl, depurination of wybutine) and aniline treatment (chain scission). Phe-GAA was detected with probe 3 from (**b**), and the control Leu-CAG with an oligonucleotide probe complementary to the ACL. U6 snRNA is used as a loading control. On the right is a quantitation of the signal intensities for Phe-GAA after HCl and aniline treatment, expressed as a ratio of the product band (tRNA halves) intensity to the precursor tRNA (full-length) intensity. Asterisk: tRNA halves. nt: nucleotides, molecular weight estimated from bromophenol blue and xylene cyanol dyes signal. **f** Bar chart of the levels of mcm^5^U and mcm^5^s^2^U nucleosides normalized to m^1^A (LC-MS/MS quantification). *n* = 2 biological replicates.

First, we verified that the faster migrating species observed in oligodendrocytes is not the result of uncharged tRNAs accumulation. Alkaline hydrolysis, which leads to complete deacylation of the tRNA, was performed on tRNAs purified from OPCs and oligodendrocytes. While alkaline hydrolysis does result in a faster migrating band, the oligodendrocyte specific doublet is still observed when the amino acid is stripped off (Fig. 3a).

Next, we determined if the faster migrating band observed in oligodendrocytes is a result of tRNA fragmentation. This was accomplished using three distinct Northern probes to walk around the transcript (D-loop, ACL and TψC-loop; Fig. 3b). In each case, we observed the exact same electrophoretic migration pattern, demonstrating the faster migrating species is not a Phe-GAA 5′ or 3′ tRNA half (tiRNA) or fragment (tRF)^65–67^. Furthermore, the fact that we still observe the fast-migrating band after deacylation confirms it is not a tRNA with cleaved CCA tail, as this would not be affected by deacylation (Fig. 3a).

We next chemically probed the presence of yW on tRNAs in the three cell types. yW can be uniquely depurinated by hydrochloric acid treatment leading to the formation of an abasic site^68,69^. Acid hydrolysis was performed at pH2.9 and this dramatically affected the mobility of the Phe-GAA tRNAs as they collapsed to the same electrophoretic level in all three cell types (Fig. 3c–d). Moreover, the collapsed band co-migrates with the faster running species that naturally occurs in oligodendrocytes, suggesting they share similar isoelectric properties under these electrophoretic conditions (Fig. 3c). As a control, we also probed for the Leu-CAG tRNA. The only mobility shift seen for this tRNA occurs upon amino acid removal. This result strongly suggests that in oligodendrocytes a fraction of Phe-GAA tRNAs bears a hypo-modified yW derivative or a precursor intermediate, that changes the hydrophobic properties of the tRNA and is thus not affected by HCl (Fig. 3d).

Finally, the yW status of the Phe-GAA tRNA in oligodendrocytes was further tested by additional chemical probing techniques. Importantly, HCl treatment of yW creates an abasic site. This abasic ribose is susceptible to chain cleavage via a β-elimination reaction. We induced chain cleavage with the use of aniline in combination with heat and low pH. This assay has been used to study the maturation of Phe-GAA tRNA in yeast^70^ (Fig. 3d). As expected, the combination of HCl and aniline treatment leads to conversion of the slow-moving, full-length species into a faster migrating species of predicted size for the two 5′ and 3′ tRNA halves (Fig. 3e). Conversion toward a small species is not observed upon aniline treatment alone. Strikingly however, conversion of the full-length species to the tRNA halves is markedly reduced in oligodendrocytes (ratio product to full-length 0.5:1) when compared to OPCs and astrocytes (ratio product to full-length 2:1) (Fig. 3e). The resistance of the full-length tRNA to aniline/HCl treatment demonstrates that a hypo-modified state for Phe-GAA exists in oligodendrocytes. As a last control, we performed the same experiment with RNA from WT and *TYW1* deletion yeast strains (Supplementary Fig. 5b, c). Phe-GAA tRNA from *tyw1∆* has a different electrophoretic mobility compared to Phe-GAA from WT (migrates faster), confirming that loss of yW can indeed affect the tRNA mobility by acid-urea PAGE (Supplementary Fig. 5b). As expected, aniline and HCl treatment leads to fragmentation in WT but not in *tyw1∆* strain (Supplementary Fig. 5c).

Altogether, we predict that the hypomodified state will impact the function of the Phe-GAA tRNA specifically in oligodendrocytes compared to OPCs (see below).

### The Lys-UUU tRNA and select mcm^5^s^2^U_34_-bearing tRNAs are hypomodified in oligodendrocytes

Analysis of the Lys-UUU tRNA by Northern blot demonstrated a distinct electrophoretic mobility signature than was observed for Phe-GAA tRNA (Figs. 2g and 3a). The faster migrating species observed in oligodendrocytes does not appear to be the result of uncharged tRNAs accumulation as it runs slightly faster than the deacylated control (Fig. 3a). We repeated our northern probe walking strategy as described above. Using two distinct Northern probes to walk around the transcript (D-loop and TψC-loop; Fig. 3b) we observed the exact same electrophoretic migration pattern for Lys-UUU tRNA. A northern probe specific to the ACL failed to work in our hands and we cannot rule out the possibility that for this tRNA, the faster migrating species is a result of fragmentation. This would be supported by the fact that the species with high electrophoretic mobility doesn’t shift under deacylation in oligodendrocytes (Fig. 3a). Nonetheless, as seen below, we demonstrate that Lys-UUU tRNA appears to be hypomodified in oligodendrocytes. It has been shown that some modifications affect tRNA cleavage (inducing or inhibiting)^67^, thus the pattern observed for Lys-UUU tRNA may be an indirect effect of hypomodification.

As mentioned, nucleotides in the ACL in Lys-UUU tRNA are known to be modified and these modifications impact its decoding potential. In particular, position U_34_ is modified to mcm^5^U/mcm^5^s^2^U. To assay mcm^5^U/mcm^5^s^2^U levels in oligodendrocytes vs. OPCs, we conducted quantitative mass spectrometry analysis. Importantly, we observed a 38% reduction in the levels of mcm^5^s^2^U in oligodendrocytes as compared to OPCs (Fig. 3f). These data support that position U_34_ of Lys-UUU is hypomodified in oligodendrocytes in the anticodon. While U_34_ modification would not strongly impede the RT and would not be observed with the variant analyses, QuantM-seq analysis suggests that A_37_ is also hypomodified in oligodendrocytes. However, we were unable to confirm ms^2^t^6^A_37_ modification status on the Lys-UUU tRNA due to a lack of chemical means and commercially available standards.

Finally, we performed PAGE for OPCs and oligodendrocytes RNA in the presence of [(*N*-acryloylamino)phenyl]mercuric chloride (APM), which leads to slower migration of sulfur-containing tRNAs compared to their unthiolated counterparts^71^. If Lys-UUU tRNA is modified in the ACL (mcm^5^s^2^U_34_ and ms^2^t^6^A_37_), it will then affect its mobility. As expected, the ratio of thiolated to unthiolated Lys-UUU tRNA was lower in oligodendrocytes than in OPCs, confirming that this tRNA is less modified in oligodendrocytes (Supplementary Fig. 5d). In addition to Lys-UUU, mcm^5^s^2^U_34_ is found in other mammalian cytoplasmic tRNAs: Glu-UUC, Gln-UUG and Arg-UCU^59,72,73^. Consistent with the decrease in the levels of mcm^5^s^2^U modification (Fig. 3f) in oligodendrocytes, we observed a decrease in the ratio of sulfur-containing vs. unthiolated tRNA for Glu-UUC and Gln-UUG in oligodendrocytes compared to OPCs, suggesting they are less modified in oligodendrocytes, while there was no mobility shift for Arg-UCU in any cell type (Supplementary Fig. 5d).

Altogether, we conclude that multiple tRNA specifies are hypomodified in oligodendrocytes, potentially impacting decoding potential within these cells.

### Genes required for modification of the anticodon loop of Phe-GAA and Lys-UUU tRNAs are poorly expressed in oligodendrocytes

**Fig. 4:**
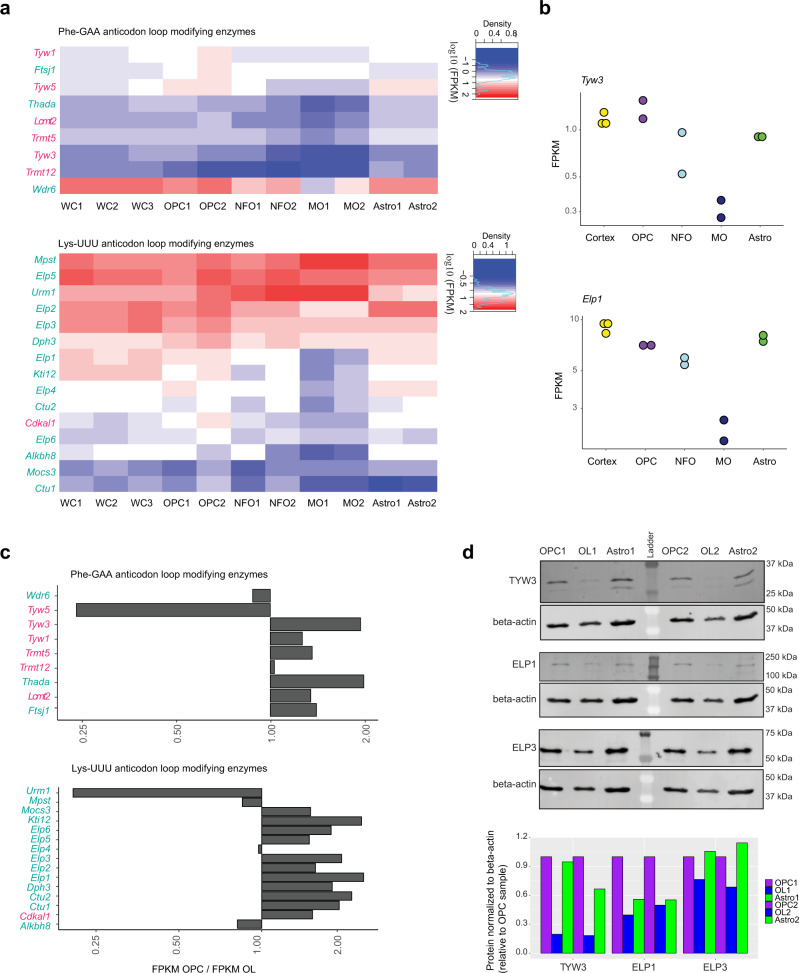
Genes required for modification of the anticodon loop of Phe-GAA and Lys-UUU tRNAs are poorly expressed in oligodendrocytes. **a** Heatmap of the expression levels (FPKM) of the factors involved in the modification pathways of Phe-GAA ACL (top) and Lys-UUU ACL (bottom), data from^111^ for the whole cortex (WC), OPCs, Newly Formed Oligodendrocytes (NFO), Myelinating Oligodendrocytes (MO) and astrocytes (Astro) purified from mouse brain. The numbers are the biological replicates numbers. **b** Expression levels (FPKM) from RNA-Seq data from (**a**) for examples of genes differentially expressed between OPCs and Myelinating oligodendrocytes (MO) (*p* < 0.05), in the whole cortex (*n* = 3 biological replicates), OPC (*n* = 2 biological replicates), NFO (*n* = 2 biological replicates), MO (*n* = 2 biological replicates) and Astro (*n* = 2 biological replicates). **c** Expression levels (FPKM) from RNA-Seq data from OPCs and OPCs differentiated to oligodendrocytes (OL), for the factors involved in the modification pathways of Phe-GAA ACL and Lys-UUU ACL, expressed as a ratio of FPKM OPC/OL. These values are the average from 5 biological replicates for OPCs and four biological replicates for oligodendrocytes. DESeq2 analysis: adjusted *p* value < 0.05 for *Thada*, *Urm1*, *Elp1*, *Elp3*, *Elp6* and *Kti12* (adjusted *p* value: Benjamini–Hochberg correction). **d** Western blot analyses of TYW3, ELP1 and ELP3 in OPCs, oligodendrocytes (OLs) and astrocytes (Astro) (OLs and Astro differentiated from OPCs). The numbers represent two biological replicates. Βeta-actin is used as a loading control. The signals from the Western blots were quantified and expressed relative to the levels in OPCs.

Collectively our data suggest that both yW and mcm^5^s^2^U levels are significantly decreased in oligodendrocytes. To investigate why this hypomodified tRNA state exists, we analyzed expression of ACL modifying enzymes from published RNA-Seq data from OPCs, newly formed oligodendrocytes (NFO), myelinating oligodendrocytes (MO) and astrocytes isolated from mouse cortex, as well as whole cortex (WC) (herein and Zhang et al., 2014) (Fig. 4a). Strikingly, several genes required for yW synthesis (such as *Tyw3*) are markedly reduced in MO relative to OPCs (Fig. 4a, b). Moreover, transcripts such as *Elp1*, *Elp3*, and *Elp6*, required for mcm^5^U/mcm^5^s^2^U modification, are also reduced in MO relatively to OPCs (Fig. 4a, b). Statistical analysis with the differential RNA-Seq expression analysis tool *limma* reveals the expression of most of these transcripts (except *Ftsj1* and *Trmt12*) is significantly different between OPCs and MOs (limma-trend *p* value < 0.05)^74^.

We further validated a decrease in the expression level of several transcripts required for Phe-GAA and Lys-UUU ACL modifications from RNA-Seq data from our OPC and oligodendrocyte cell model (Fig. 4c) (DESeq2 analysis, adjusted *p* value < 0.05 for *Thada*, *Elp1*, *Elp3*, *Elp6* and *Kti12*). Importantly, a decrease in the protein levels of TYW3, ELP1 and ELP3 in oligodendrocytes compared to OPCs or astrocytes was also confirmed by Western Blot analysis (Fig. 4d).

These data demonstrate that several of the modifying enzymes for the ACL of Phe-GAA, ACL of Lys-UUU and mcm^5^s^2^U-containing tRNAs are less expressed in oligodendrocytes compared to other cell types, possibly altering their function.

### The cognate codons for Phe-GAA and Lys-UUU tRNAs have altered impact on mRNA decoding and stability in oligodendrocytes

The impact of mcm^5^s^2^U on the translation of AG or AA-ending codons is not completely understood in all organisms. Nonetheless, loss of mcm^5^s^2^U leads to decreased translation of transcripts enriched in the AAA codon^75,76^. Similarly, hypomodification at both G_34_ and G_37_ of Phe-GAA tRNA specifically decreased the translation efficiency of genes enriched in the UUU codon but not the UUC codon in human cells^77^.

**Fig. 5:**
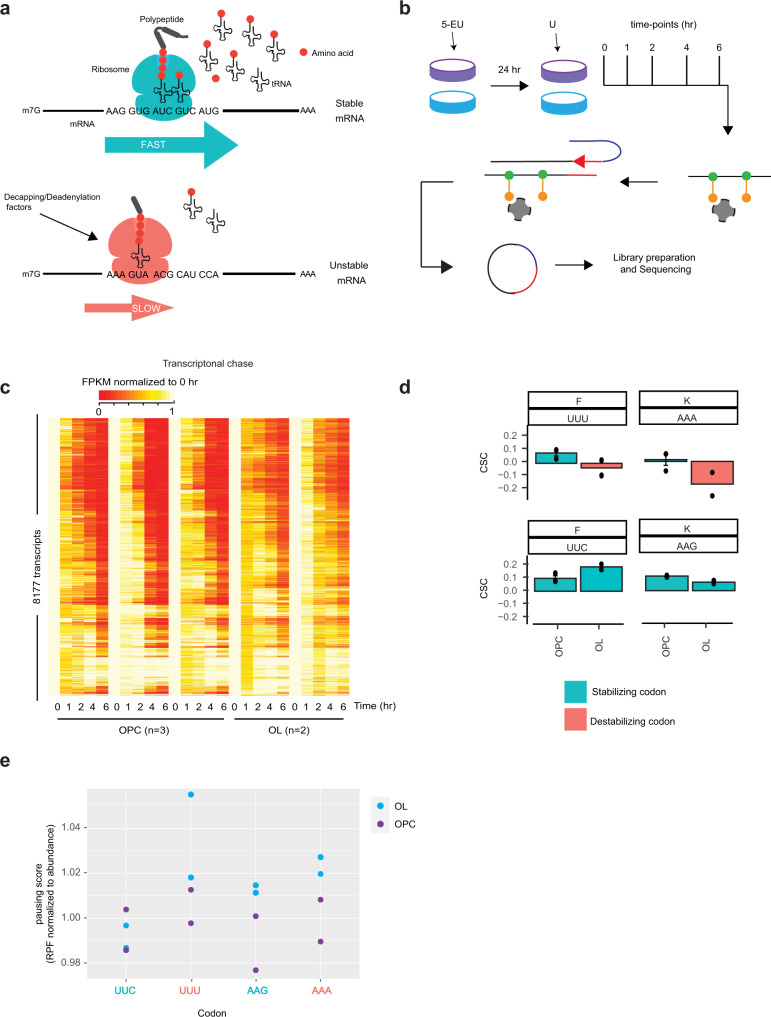
The cognate codons for Phe-GAA and Lys-UUU tRNAs have altered impact on mRNA decoding and mRNA stability in oligodendrocytes. **a** Ribosome translocation on each codon depends on the availability of functional tRNAs, which affects translation efficiency and Codon Optimality-Mediated mRNA Degradation. **b** Outline of Decay-Seq performed in OPCs and oligodendrocytes. 5-EU was added to cells then replaced by U and RNA was collected at various time-points. 5-EU (green circles)-labelled RNAs were coupled to biotin (orange circles) and specifically pulled down with streptavidin beads (gray). After adapter ligation (red line) and reverse transcription (red arrow) on beads, cDNA was eluted, circularized and amplified before sequencing. **c** Heatmap of the FPKM of each transcript at every time-point after removal of 5-EU, normalized to 0 h, in OPCs and oligodendrocytes replicates. *n* = number of biologically independent experiments. **d** CSC values for the synonymous codons of phenylalanine and lysine in OPCs and oligodendrocytes. Represented are the values of three biological replicates in OPCs and two biological replicates in oligodendrocytes (dots), with the mean plotted as a bar (mean ± SEM for OPCs). **e** Ribosome pause scores for the Phe and Lys codons in OPCs (purple dots) and oligodendrocytes (blue dots). The codons with optimal CSC value in oligodendrocytes are labelled in tile color on the *x*-axis, non-optimal codons in pink. Represented are the pause score values of two biological replicates.

Translation decoding efficiency and mRNA stability are intimately connected through CO-MD^6,10,11^. Since CO-MD is a function of each tRNA’s decoding potential, oligodendrocytes may alter CO-MD concomitantly to their altered tRNA landscape (Fig. 5a). To test this hypothesis, we determined global mRNA decay rates in OPCs and oligodendrocytes by combining metabolic labelling, Click-IT chemistry and deep RNA sequencing^5^ (Fig. 5b). In brief, cells were incubated in the presence of 5-ethynyluridine (5-EU) for 24 h. Following, 5-EU was removed from the media and replaced by uridine. Time points were then collected during the ensuing chase. Critically, a spike-in 5-EU labeled control was added to each time point prior to library preparation, allowing for robust quantitation. Using this approach, we robustly determined the half-life of about 8000 transcripts common between OPCs and oligodendrocytes (Fig. 5c).

We previously established the Codon Stabilization Coefficient (CSC) as a means to empirically determine how each of the 61 codons contribute to overall transcript half-lives^6^. The CSC metric has been used to successfully determine the general contribution of codons to mRNA stability in budding and fission yeast, zebrafish, drosophila, mice, CHO cells, and humans^4,5,8,78^. The CSC for each codon is an empirical metric rooted in decoding efficiency. Negative CSC values represent codons that cause significant ribosome pauses that can create a ribosomal conformation recognized by the mRNA deadenylase/decapping complex, stimulating mRNA decay (Fig. 5a)^9,79^. Optimal codons with positive CSC evade recognition by the deadenylase/decapping complex, thereby stabilizing the mRNA. It is important to point out that other mRNA features contribute to transcript stability, but CSC is a reliable metric that can predict a large amount of the variance in transcript half-lives^80^ via changes in decoding potential. We calculated the CSC for every codon in OPCs and oligodendrocytes. Both cell types show codons preferentially enriched in stable or unstable transcripts, confirming the correlation between codon content and mRNA decay in mammalian cells (Supplementary Fig. 6a). In the rest of this study, we will define optimal/non-optimal codons as codons with positive/negative CSC values respectively.

In theory, lower levels of Phe-GAA and hypomodification of the Phe and Lys tRNAs in oligodendrocytes could manifest in change in how their cognate codons elicit CO-MD. Accordingly, the Phe-encoding UUU and Lys-encoding AAA codons switch in optimality between OPCs and oligodendrocytes. In OPCs, both UUU and AAA codons are rather optimal (i.e., transcript stabilizing), while in oligodendrocytes, both UUU and AAA are non-optimal/ transcript destabilizing (Fig. 5d). Furthermore, there is a clear flip in optimality in oligodendrocytes for their respective synonymous codons UUC and AAG while they remain optimal in OPCs (Fig. 5d). Both UUU and UUC codons are decoded by the single Phe-GAA tRNA. Moreover, Lys-UUU tRNA decodes both AAA and AAG codon albeit AAG to a lesser extent due to the presence of Lys-CUU tRNA in cells. Interestingly, replacing Phe UUC by UUU codons and Lys AAG by AAA codons in an eGFP reporter (85% optimality in oligodendrocytes) in oligodendrocytes results in a 50% decrease in the transcript levels of the reporter (Supplementary Fig. 6b). Furthermore, switching all the possible codons of the eGFP sequence to their synonymous non-optimal codons (final optimality 13%) reduced the transcript levels by more than 95% in oligodendrocytes.

Interestingly, the codons GAA and GAG, decoded by the tRNA Glu-UUC that showed the highest difference between OPC and oligodendrocytes by APM PAGE (Supplementary Fig. 5d), showed a similar pattern as Lys codons, with a flip from non-optimal for GAA to optimal for GAG that is specific to oligodendrocytes (Supplementary Fig. 6c). Although CAA was less optimal than CAG in oligodendrocytes, all the codons decoded by Gln-UUG and Arg-UCU were non-optimal in both OPCs and oligodendrocytes (Supplementary Fig. 6c).

Taken together, it is possible that the distinctions in CSC values observed in oligodendrocytes vs. OPCs are manifestations, at least in part, of the alterations in the tRNA landscape of oligodendrocytes.

Importantly, CO-MD is elicited by ribosome hesitations in response to limited functional tRNA pools. To evaluate ribosome pausing over UUU and/or AAA codons in oligodendrocytes, we performed ribosome profiling. We used micrococcal nuclease (MNase) rather than RNase I to generate the ribosome-protected fragments, as has been done previously, to preserve monosome integrity^81^ (Supplementary Fig 7a). Footprints of about 30–31 nucleotides were obtained, that mapped predominantly to the coding regions of transcripts (Supplementary Fig 7c–e). Because of reads homogeneity and sequence specificity, it is challenging to assess the P- and A-site with MNase-produced footprints. Nonetheless, by estimating P-sites from the 3′end of reads, we observed 3-nucleotide periodicity in the CDS (although it has been suggested to come from the sequence specificity of the MNase^82^) (Supplementary Fig 7d), and a majority of the reads are in frame (Supplementary Fig 7e). We first looked at ribosome footprints density over mRNA abundance as a proxy for translational efficiency (Ribosome Density, RD). Translation is correlated with mRNA stability, as ribosome density increases with increasing half-life in both OPCs and oligodendrocytes (Supplementary Fig. 8a). To estimate ribosome pausing, and due to the MNase limitations as described above, we quantified the average ribosome footprint density in a restrictive window around each codon normalized to the coverage on the whole CDS, as in Darnell et al^81^. This gave us the overall difference in ribosome density at codons without the need for frame information. Strikingly, in oligodendrocytes, non-optimal codons have a higher ribosome occupancy score than their optimal synonymous codon (Supplementary Fig. 8b), suggesting ribosome slow down or stalling at these codons. Importantly, the codons UUU, AAA and AAG have a higher pause score in oligodendrocytes than in OPCs, and the difference UUU/UUC and AAA/AAG is higher in oligodendrocytes than in OPCs (Fig. 5e). This suggests that Phe-GAA and Lys-UUU tRNAs, which are less modified in the ACL in oligodendrocytes, lead to extended ribosome pausing on the UUU and Lys codons.

Consistently, the difference in pausing score and CSC between the two cell types shows an opposite relationship, with the difference oligodendrocytes to OPCs for UUU, AAA and AAG being positive for ribosome pausing but negative for CSC (Supplementary Fig. 8c). Importantly, although the changes in pause score and CSC between OPCs and oligodendrocytes are not restricted to Lys and Phe codons, these codons represent some of the highest difference values, confirming their importance in CO-MD of the differentiated oligodendrocytes (Supplementary Fig. 8c).

### mRNAs with a low UUU and AAA codon bias are stabilized in oligodendrocytes and are enriched in plasma membrane components

**Fig. 6:**
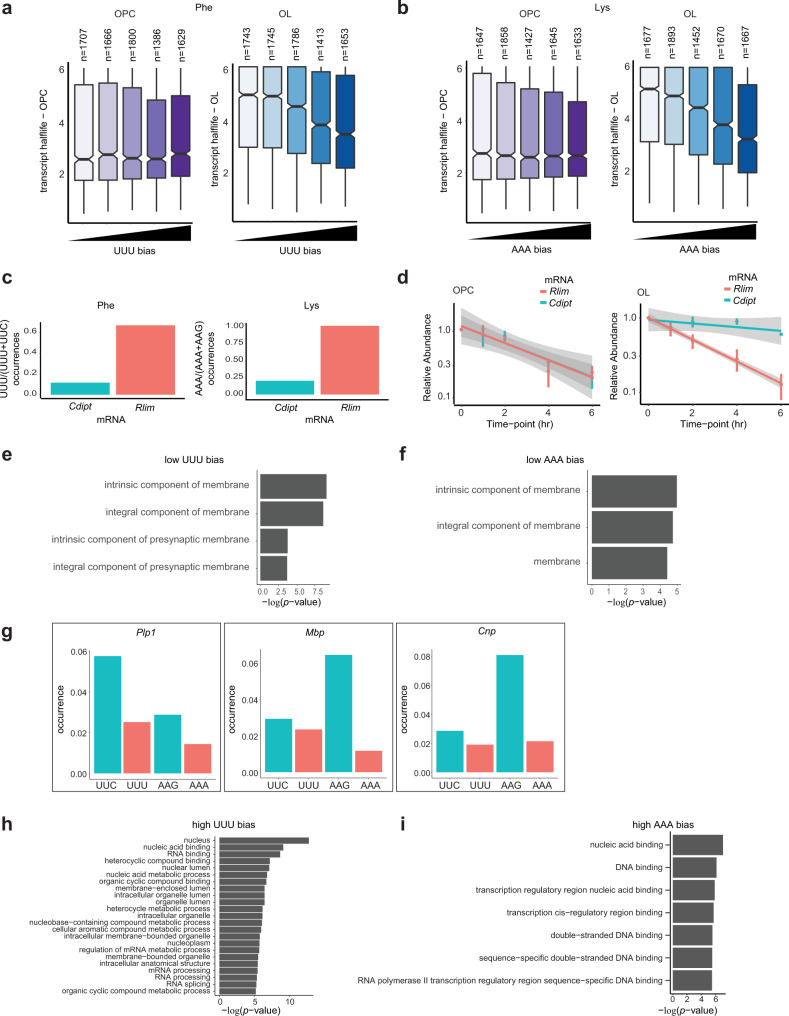
mRNAs with a low UUU and AAA codon bias are stabilized in oligodendrocytes and are enriched in plasma membrane components. **a–b** Boxplots of transcript half-lives binned by increasing occurrence of UUU over Phe codons (**a**) and occurrence of AAA over Lys codons (**b**), in OPCs (purple) and oligodendrocytes (OL, blue). Boxplots display the median with hinges at the 25th and 75th percentiles and whiskers extending 1.5 times the interquartile range. Number of transcripts in bin is indicated above each boxplot. Kruskal–Wallis test *p* value = 0.71 (OPC) and 2.2 × 10^−16^ (OL) in (**a**), 0.68 (OPC) and <2.2 × 10^−16^ (OL) in **b**. **c** Example of transcripts with a low occurrence of UUU and AAA (*Cdipt*) or a high UUU and AAA codon bias (*Rlim*). **d** Decay of the transcripts from (**c**) in OPCs and oligodendrocytes. Plotted are the relative FPKM over time (mean of 3 and 2 biological replicates for OPCs and oligodendrocytes, respectively), with the linear regression in gray shade. **e–f** GO term analysis of the transcripts with a low UUU bias over Phe codons (Bins of lowest values from Fig. 6a) (**e**) and a low AAA bias over Lys codons (Bins of lowest values from Fig. 6b) (**f**). Represented are the GO classes that are over-represented compared to the whole list of transcripts expressed in oligodendrocytes (Panther, Fisher exact test with Benjamini–Hochberg false discovery rate correction (FDR), adjusted *p* value < 10^−3^). **g** Occurrence of the Phe (UUC and UUU) and Lys (AAG and AAA) codons in the oligodendrocytes markers *Plp1* (*proteolipid protein 1*), *Mbp* (*myelin basic protein*) and *Cnp* (*2’,3’-cyclic nucleotide 3’ phosphodiesterase*) (canonical isoform sequence). **h–i** GO term analysis of the transcripts with a high UUU bias over Phe codons (Bins of highest values from Fig. 6a) (**h**) and a high AAA bias over Lys codons (Bins of highest values from Fig. 6b) (**i**). Represented are the GO classes that are over-represented compared to the whole list of transcripts expressed in oligodendrocytes (Panther, Fisher exact test with Benjamini–Hochberg FDR correction, adjusted *p* value < 10^−5^).

Having observed distinct flip in codon optimality and ribosome pausing for UUU and AAA codons in oligodendrocytes vs. OPCs, we next evaluated their influence on CO-MD. To evaluate UUU codon usage on CO-MD, we calculated the occurrence of the codon UUU normalized to UUU and UUC (codon bias) in each transcript. This value was then binned into increasing ratios of UUU bias and compared to mRNAs half-life. Importantly, while the mean of half-lives doesn’t change significantly between the different bins in OPCs, there is a marked decrease in mRNA stability as UUU occurrence increases in oligodendrocytes (Fig. 6a, OPC *p* = 0.71, oligodendrocytes *p* < 2.2 × 10^−16^, Kruskal–Wallis test). A similar correlation is observed for the lysine AAA (Fig. 6b, OPC *p* = 0.68, oligodendrocytes *p* < 2.2 × 10^−16^, Kruskal–Wallis test). Shown in Fig. 6c–d are specific examples. For instance, the *Cdipt* (CDP-diacylglycerol—inositol 3-phosphatidyltransferase) transcript is enriched in UUC vs. UUU codons and in AAG vs. AAA codons (Fig. 6c). Conversely, the *Rlim* (ring finger protein, LIM domain interacting) transcript is highly enriched in UUU and AAA. While *Rlim* and *Cdipt* share similar half-lives in OPCs, in oligodendrocytes *Cdipt* is more stable than *Rlim* (Fig. 6d). Together, these data support the hypothesis that enrichment of UUU or AAA codons (vs. their synonymous counterpart) enhances CO-MD specifically in oligodendrocytes.

More specifically, our data indicate that genes containing a high percentage of either UUU or AAA codons are going to be significantly downregulated in oligodendrocytes. We would predict, therefore, that genes required for oligodendrocyte function most likely have evolved to limit UUU and AAA codon usage. To test this hypothesis, we looked at GO term over-representation in the first and last bins of codon bias from Fig. 6a–b. The transcripts with a low ratio of UUU (normalized to Phe codons, ratio 0-0.286, *n* = 1743) or AAA (normalized to Lys codons, ratio 0-0.226, *n* = 1677) are enriched in ‘components of membranes’ (Fig. 6e–f). This suggests that transcripts which are particularly important for the biology of oligodendrocytes membranes and are long-lived in oligodendrocytes favor the usage of the optimal Phe and Lys codons. Consistently, mature CNS oligodendrocytes express a high amount of a few proteins such as PLP1, MBP or CNP (2′,3′-cyclic nucleotide 3′-phosphodiesterase), which are essential components of the myelin^83^. As suggested above, their mRNAs are characterized by a lower occurrence of UUU and AAA compared to their optimal counterpart (Fig. 6g).

Conversely, the transcripts with the highest ratio of UUU and AAA (0.615-1 for Phe, *n* = 1653, and 0.521-1 for Lys, *n* = 1667) are enriched in classes such as ‘nucleic acid binding’, ‘RNA processing’ and ‘transcription’ (Fig. 6h, i). Oligodendrocytes are highly differentiated cells expressing select transcripts once mature. Genes that are the most affected by tRNA hypomodification and downregulated in oligodendrocytes may underlie a decrease in global transcription and functions associated to cell proliferation in these cells.

Taken together, our collective findings suggest that the hypomodified tRNA environment of oligodendrocytes presents a translational challenged environment that can limit gene expression at the level of mRNA stability and protein expression. Moreover, the transcriptome of oligodendrocytes has evolved to circumvent these challenges through selective codon usage away from affected tRNAs.

## Discussion

Our study provides the description of tRNA biology and mRNA stability in murine OPCs and oligodendrocytes. We demonstrated that at least two tRNAs, Phe-GAA and Lys-UUU, are differently modified in oligodendrocytes compared to their precursor cells OPCs. These hypomodified tRNAs may be the result of the reduced expression of modification enzymes in oligodendrocytes. Moreover, we demonstrate a possible biological consequence to the oligodendrocyte-specific hypomodified tRNA state in that CO-MD is altered in a concomitant pattern. Moreover, we observe increased ribosome pausing over UUU and AAA codons in oligodendrocytes vs. OPCs. Interestingly, genes required for oligodendrocyte function are biased away from affected codon usage, implicating that these cells are translationally challenged by the tRNA hypomodification landscape. Lastly, this observed tRNA phenotype is specific to oligodendrocytes and not shared by astrocytes; another type of glial cells with a different function in the CNS.

Transfer RNA modification is critical to the decoding potential within a cell. Modifications in the ACL of Phe-GAA and Lys-UUU tRNAs, both at position 34 and 37, are important to maintain a correct reading frame on Phe and Lys codons^84–87^. In yeast, mutation in the wybutosine pathway results in ribosome pausing on Phe codons^88^. Furthermore, yeast and nematodes lacking mcm^5^s^2^U tRNA modification, exhibit ribosome pausing at AAA and CAA codons^88–91^.

Critically a hypomodified tRNA state is known to create cellular stress. A common feature in *S. cerevisiae* and *C. elegans* deficient for mcm^5^s^2^U_34_, is impairment of protein homeostasis, characterized by an aggregation of endogenous proteins and an increase of the ubiquitin-proteasome system genes. Moreover, the conditional deletion of *Elp3* in cortical neuron progenitors in mouse, which led to ribosomal pausing on AAA and AGA codons, triggered the Unfolded Protein Response, leading to neurogenesis defects^71^. It has also been shown that reduced expression of ELP3 in NSC34 cells is correlated with an increased abundance of insoluble mutant SOD1 responsible for Amyotrophic Lateral Sclerosis^92^. Together, these data argue that appropriate tRNA-modification status is required for appropriate cellular homeostasis.

Despite the observed hypomodified tRNA state that naturally exists in oligodendrocytes, we did not observe signatures of cellular stress hallmarked by nonnatural perturbation of tRNA processing. Nonetheless, we do observe in oligodendrocytes ribosome pausing and a decrease in mRNA stability for transcripts enriched in UUU and AAA. So why does a hypomodified tRNA landscape exist in oligodendrocytes?

It is important to consider that oligodendrocytes produce an enormous amount of myelin-related proteins such as PLP1, characterized by folding of its transmembrane domains that would support its structural function in the myelin membrane^83,93^. Surprisingly, full *PLP1* gene deletions are associated with the mildest form of the hypomyelinating leukodystrophy PMD, Pelizaeus-Merzbacher disease, while rare aneuploidy events, supernumerary copies, nonsense, missense, and partial frameshift or in-frame deletions, present with severe symptoms, suggesting that over-production and production of aberrant proteins is the most severe trigger of myelin dysfunction in PMD^52,94^. Further, it has been suggested that non-optimal codons, which lead to ribosome slowing during elongation, locally influence pausing and protein folding^1,2,95–98^. It is tempting to speculate that hypomodification of tRNAs in oligodendrocytes is relevant to their function, leading to different transit times of the ribosomes on specific codons, necessary for protein folding. Indeed, the frequency of non-optimal codons in transcripts like *Plp1* is low but not zero (Fig. 6c), suggesting their importance throughout the sequence. In the case of a transmembrane protein like PLP1, it may be important to both optimize translation and co-translational folding. However, in the context of mutations that affect tRNA charging or protein trafficking, the exacerbation of this phenomenon may lead to protein aggregation and disruption of the myelin or death of the oligodendrocytes.

Interestingly, we noticed an overall increase in mRNA stability in oligodendrocytes compared to OPCs (Fig. 5c and Fig. 6a, b), suggesting additional factors regulating mRNA stability, such as RNA binding proteins. For example, quaking (QKI) is a protein that is highly upregulated in MO, and involved in the stabilization and transport of mRNAs that are targeted to the myelin compartment^99^. Especially in mature oligodendrocytes expressing a define population of myelin transcripts at very high levels, such factors may interplay with codon effects to regulate codon optimality-mediated mRNA decay. And while there may be other factors explaining the difference in stability between OPCs and oligodendrocytes, our data suggest that a change in some tRNA levels and modifications influence CO-MD in the differentiated oligodendrocytes. Finally, as suggested by our results with Glu-UUC tRNA and the number of codons affecting CO-MD differently in oligodendrocytes and OPCs, more tRNAs may differ between these cells and would need further investigation.

As mentioned, myelin disorders associated with oligodendrocytes and hypomyelination often result when the ribosome/tRNA axis is impaired. Examples include AIMP1/p43 (aminoacyl tRNA synthetase-interacting multifunctional protein 1), a noncatalytic component of the mammalian multi-tRNA synthetase complex that consists of 9 different ARSs^100^. Loss-of-function mutations in AIMP1 lead to hypomyelinating leukodystrophy-3 disease (HLD3). Likewise, mutations in subunits of POLR3, that synthetizes tRNAs, underlie an important group of myelin defects. In addition, tRNA modification factors have been associated to intellectual disability in humans^101,102^. Given our observations, it is important to consider that the naturally hypomodified tRNA landscape present in oligodendrocytes could predispose these cells to disease when further insult is made to the translational apparatus. We anticipate therefore, that it is the unique tRNA/mRNA landscape that exists naturally in oligodendrocytes that hypersensitizes these cells toward leukoencephalopathies when translation is further impaired.

## Methods

### Pluripotent stem cell-derived OPC culture

Mouse protocols were approved by Case Western Reserve University School of Medicine’s Institutional Animal Care and Use Committee. Mouse (*Mus musculus*) OPCs were generated from epiblast stem cells (EpiSCs) as in ref. 50. In brief, EpiSCs were isolated from 129 S/SvEv male embryos (E3.5; The Jackson Laboratory) and pushed to form neural rosettes^51^. Neural rosettes were then passaged into nunclon plates coated with poly-L-ornithine (PO) and laminin, in OPC growth media consisting of DMEM/F12 supplemented with N2 Max (R&D Systems, AR009), B27 (Thermo Fisher, 12587010), 20 ng/mL bFGF (R&D Systems, 23-3FB-01M), and 20 ng/mL PDGFA (R&D Systems, 221-AA). Media was changed every 48 h and cultures were maintained in 37 °C with 5% CO_2_.

### Mouse OPC differentiation to oligodendrocytes and astrocytes

For OPC differentiation to oligodendrocytes, OPCs were grown through two passages in OPC growth media without bFGF. They were then seeded onto dishes coated with PO and laminin, at a density of 5 million cells on 10 cm dishes, 12.5 million cells on 15 cm dishes, or 1 million/well of six-well plates. They were differentiated in oligodendrocyte differentiation media that consisted of DMEM/F12 supplemented with N2 Max, B27, 100 ng/mL noggin (R&D, 3344NG050), 100 ng/mL IGF-1 (R&D, 291G1200), 10 uM cyclic AMP (Sigma, D0260-100MG), 10 ng/mL NT3 (R&D, 267N3025) and 40 ng/mL T3 (thyroid hormone, Sigma, T-6397). Cells were analyzed after 68–72 h.

For astrocyte generation, 4 million OPCs were plated on 10 cm dishes or 10 million on 15 cm dishes containing astrocyte differentiation media, which consisted of a 1:1 (v/v) mixture of neurobasal media and high glucose DMEM supplemented with sodium pyruvate, glutamax, N2 Max, and N-acetyl-cysteine and with growth factors including 20 ng/mL bFGF, 5 ng/mL Hb-EGF (R&D, 259-HE-050), 10 ng/mL CNTF (R&D, 557-NT-010), and 10 ng/mL BMP4 (R&D, 314-BP-050), for 3 days^50^.

### Immunocytochemistry

Staining and imaging were performed as in ref. 50. For O1 antigen live staining, antibodies were diluted in N2B27 base media supplemented with 10% Donkey Serum (v/v) (Jackson ImmunoResearch, 017-000-121) and added to cells for 18 min at 37 °C. Cells were then fixed in cold 4% PFA for 18 min at room temperature, washed with PBS, and permeabilized and blocked in blocking solution (0.1% Triton X-100 in PBS supplemented with 10% normal donkey serum (v/v)) for 30 min at room temperature. Primary antibodies were incubated overnight at 4 °C in blocking solution. The primary antibodies anti-MBP (1:100, Abcam, ab7349), anti-O1 (1:50, CCF Hybridoma Core), anti-O4 (1:100, CCF Hybridoma Core), anti-OLIG2 (1.2 mg/mL, Proteintech, 13999-1-AP), anti-GFAP (1:5000, Dako, Z033401-2) and anti-PLP1 (1:1000, Lerner Research Institute Hybridoma Core) were used. The next day, cells were rinsed with PBS and incubated in blocking solution for 1 h with the appropriate secondary antibody conjugated to an Alexa Fluor (4 μg/mL, Thermo Fisher) along with the nuclear stain DAPI (Sigma, 1 μg/mL).

For quantitation in Fig. 1b, plates were imaged using the Operetta High Content imaging and analysis system (PerkinElmer). Images were analyzed with PerkinElmer Harmony and Columbus software. In brief, each DAPI positive nucleus of live cell was expanded by 50% to determine potential intersection with staining of GFAP, MBP, PLP1 or O1 in a separate channel. Percentages were then calculated by dividing the number of each of these markers positive cells by the total number of DAPI positive cells per image.

### RNA extraction

RNA was extracted with Trizol (Thermo Fisher Scientific, 15596018) following the manufacturer’s protocol. Media was removed from cells grown into 10 cm dishes, and 2 mL of Trizol were added directly to the plates. The cells were homogenized by pipetting up and down and incubating in Trizol for 5 min. 200 μL of chloroform were added to each 1 mL of Trizol, the samples were vortexed briefly, incubated for 3 min at room temperature then centrifuged at 4 °C and 12,000 × *g* for 15 min. The aqueous phase was then precipitated with an equal volume of isopropanol for 10 min at room temperature and centrifuged at 4 °C for 10 min. The RNA was washed twice with 75% ethanol and resuspended in water except for tRNA analyses.

### RT-PCR

Reverse transcription was performed with 1.5 μg of RNA extracted with Trizol and resuspended in water, with Superscript III (18080-044, Thermo Fisher Scientific) and random primers. The RT was omitted from the reaction for the negative controls. A tenth of the cDNA was used for PCR reactions using Phusion polymerase (M0530L, New England Biolabs) as per the manufacturer’s protocol, and the primers listed in Supplementary Table 1, for 24 cycles of amplification. The amplicons were run on 2% agarose gels and visualized with ethidium bromide staining and a GelDoc imaging system.

### tRNA sequencing by QuantM-seq

QuantM-seq was performed as in ref. 13. In brief, total Trizol isolated RNA resuspended at 1 µg/µL was deacylated at 37 °C for 45 min in 20 mM Tris-HCl pH 9. For each library preparation, 1 µg of deacylated total RNA was annealed to a double-stranded adapter, obtained by annealing of a 3′ single-stranded adapter and a 5′ single-stranded adapter mix, with RNA ligase 2 (0.5 U/µL) at 37 °C for 1 h then 4 °C for 1 h. After ethanol precipitation and resuspension in 10 µL of water, reverse transcription was performed with 10 pmol of RT primer using Superscript IV (Thermo Fisher Scientific, 18090010) at 55 °C for 1 h (the same RT batch was used for all the samples). RNA was hydrolyzed with 0.1 N NaOH at 98 °C for 20 min and the cDNA was precipitated with ethanol. The cDNA was then separated using 7 M urea 6% denaturing polyacrylamide gels, stained with 1× SYBR gold in 1× TBE for 15 min, and extracted overnight in DNA elution buffer (300 mM NaCl, 10 mM Tris-HCl pH 8, 1 mM EDTA). Following isopropanol precipitation, the cDNA was circularized with CircLigase at 60 °C for 1 h, precipitated, and amplified with the NEBnext Ultra II Q5 next-generation master mix (M0541S, NEB) (Supplementary Table 1). Amplified libraries were purified from 2% agarose gels stained with ethidium bromide and extracted using the Qiaquick gel extraction kit (Qiagen). Sequencing was performed as single-end reads for 110 cycles on a NextSeq 550 (v2.5).

### QuantM-seq analyses

QuantM-seq data was processed as in Pinkard et al, 2020. 5′ adapter and 3′ CCA and adapter sequences were removed from reads using cutadapt v.2.8 (-g TCCAACTGGATACTGGN and -a CCAGTATCCAGTTGGAATT, respectively). Example of bash command line is as follows:

cutadapt—cut 2 -o temp.fastq.gz $x

cutadapt -g TCCAACTGGATACTGGN -e 0.2 -o temp2.fastq.gz temp.fastq.gz

cutadapt -a CCAGTATCCAGTTGGAATT -e 0.2 -o ${x%%.fastq.gz}.fastq.trimmed.gz temp2.fastq.gz

Reads were then mapped with bowtie2 v.2.42^103^ to a mouse tRNA reference generated from GtRNAdb mm10 high-confidence mature tRNA fasta file^104^ (or GtRNAdb S288c high-confidence mature tRNA set for yeast). To generate this reference, tRNAs were collapsed into single genes for tRNAs with multiple identical genes. Very sensitive local alignment mode was used as follows:

bowtie2—quiet—min-score G,1,8—local -D 20 -R 3 -N 1 -L 10 -i S,1,0.5 -p 3 -x $tRNA_reference.fa -U ${x%%.fastq.gz}.fastq.trimmed.gz -S ${x%%.fastq.gz}.sam

The sam files were converted to bam, and read count tables for each isodecoder were produced with Rsubread package’s featureCounts function in R^105^. Reads were summed to generate anticodon-level tables, and both raw reads or RPM tables were obtained for tRNAs grouped by isodecoders or anticodons. Differential tRNA expression analysis was performed with DESeq2 in R using default settings and *p* value adjustment (Benjamini–Hochberg correction)^106^.

Variants in tRNA sequencing reads were computed for every tRNA in each of the cell types using a custom python script, that summed four types of variants at each position: mutation, insertion, or deletion were inferred by using the CIGAR string and MD tags from the bam files; and sites of RT stalling or fall-off were tabulated from 5′ ends of reads internal to tRNA.

All the scripts used (alignment, python and R scripts) are provided in “Supplementary software” of Pinkard et al^13^. and in this paper Supplementary files (Code).

The read density per nucleotide at each position of the tRNAs were computed from the bam files with samtools depth^107^. The density at each position was averaged between the different biological replicates and expressed as a ratio OPC/oligodendrocyte. Downstream data visualization and plotting were performed with R v4.1.1 (R Core Team 2021, https://www.R-project.org/) and RStudio v1.4.1717 (RStudio Team 2021, http://www.rstudio.com/), using ggplot2^108^ and gplots (heatmap.2)^109^.

### tRNA treatments before electrophoresis

For tRNA Northern Blots, the pellet of RNA extracted with Trizol was resuspended in 10 mM sodium acetate buffered with acetic acid at pH 4.5, 1 mM EDTA. The RNA was kept on ice at all times to preserve aminoacylation of the tRNAs, except when submitted to further treatments as follows^110^. 2.5–5 μg of total RNA was used for each individual treatment. For complete deacylation of the tRNAs, the RNA was resuspended in water and Tris-HCl pH 9 was added to a final concentration of 200 mM. The samples were incubated at 37 °C for 40 min. For HCl induced depurination of wybutosine, the RNA was resuspended in water and HCl added to a final concentration of 1.2 mM (pH 2.9) and incubated at 37 °C for 2 h 15 min^68,69^. For Aniline induced chain scission, the RNA was first treated with HCl as above (this step was not performed for control with aniline only), quenched with KOH 0.93 mM, then incubated with an equal volume of 0.5 M aniline (Millipore Sigma, 242284-100 ML) pH 4.5 for 20 min at 60 °C^70^. Following the different treatments as well as for the untreated controls, the samples were then precipitated by adding 1 μL glycoblue, 4 μL NaCl 5 M, 4 μL buffered acetate 1 M and water to a final volume of 200 μL, then 600 uL of 100% ethanol. After precipitation at −20 °C from 1 h to overnight, the samples were centrifuged at 12,000 *g* at 4 °C for 20 min, washed twice with 75% ethanol, and resuspended in 10 mM sodium acetate pH 5.2.

### Acid urea polyacrylamide gel electrophoresis and Northern blot

We followed the protocol from Köhrer and RajBhandary^54^ to analyze tRNAs by acid urea PAGE. An equal volume of acid urea sample buffer (0.1 M NaOAc pH 5.2, 8 M urea, 0.05% bromophenol blue, 0.05% xylene cyanol) was added to 2 μg of the RNA samples resuspended in sodium acetate as described above. The samples were kept cold and ran on acid urea gels (6.5% polyacrylamide 19:1, 0.1 M NaOAc pH 5.2, 8 M urea; 0.4 mm × 20 cm × 30 cm), in 0.1 M NaOAc pH 5.2, at 280 V for 14 to 17 h, at 4 °C. The samples were then transferred to Hybond-N membranes (Fisher, RPN303N) in 1X transfer buffer (40 mM Tris-HCl pH 8, 2 mM Na_2_EDTA) at 15 V for 4.5 h at 4 °C. The RNA was crosslinked to the membrane twice at 140 mJ/cm2, then washed for 1 h at 65 °C in 0.1 X SSC/0.1% SDS. The blots were pre-hybridized either in 6X SSC, 10X Denhardt’s, 0.1% SDS at 42 °C for short oligomer probes (∼20 nt), or in 2X SSC, 10X Denhardt’s, 0.1% SDS at 60 °C for whole tRNA probes (∼73 nt). They were incubated in the same buffer/temperature overnight with probes that were end-labelled with ɣ−32P-ATP using T4 Polynucleotide Kinase (Supplementary Table 1). Short probes were washed with 6X SSC, 0.1% SDS twice at room temperature for 15 min, then once at 50 °C for 20 min. Full-length probes were washed twice at room temperature for 15 min with 2X SSC, 0.1% SDS, then once at 65 °C for 45 min in 0.5X SSC, 0.1% SDS. Blots were exposed on a storage phosphor screen for 1 h to overnight. Signal was read using an Amersham Typhoon 5 laser-scanner platform (Cytiva). Quantitation of phosphorimager signal was performed using ImageQuant (Molecular Dynamics; version 5.2) and Image Lab (Biorad) softwares. Further data visualization and plotting were performed in R using ggplot2.

### Yeast strains, growth conditions and RNA extraction

The genotypes of the *Saccharomyces cerevisiae* strains used in this study are listed in Supplementary Table 1. Strains are based on BY4741. Cells were grown in Yeast extract-Peptone-Dextrose media at 24 °C. They were collected at mid-log phase (3 × 10^7^ cells. mL^−1^). Cell pellets were vortexed in Trizol reagent (1 mL per 50 mL yeast culture) with glass beads, for 5 min, then incubated at room temperature for 5 min. They were vortexed again for 5 min after addition of 200 uL chloroform, then processed the same as described above for mammalian cells.

### LC-MS/MS quantitative analysis

Quantification of mcm^5^U, mcm^5^s^2^U and m^1^A was performed by the RNA Epitranscriptomics & Proteomics Resource at the University at Albany. Small RNA was purified using the RNA Clean and concentrator Kit (R1015, Zymo research), following the manufacturer’s protocol for purification of small and large RNAs into separate fractions. 500 ng were adjusted with 10 μL water followed by digestion to nucleosides using the Nucleoside Digestion Mix (NEB, M0649S) for 1 h at 37 °C. The resulting nucleoside mixtures were then reconstituted to 100 μL using 0.01% formic acid. LC-MS/MS analysis was performed with a Waters I-Class UPLC conFig.d with triple quadrupole mass spectrometer Waters Xevo TQ-S equipped with step-way technology and an ESI-source maintained at 500 °C, capillary voltage of 0.95 kV and extraction cone of 18 V. Modified nucleoside standards were used to obtain calibration curves: a method to extract peak areas from raw data to allow quantification was developed using a combination of instrument manufactures software suites: MassLynx 4.1 and TargetLynx (Waters). This allowed extraction of information to produce calibration curves and quantify the concentration of each nucleoside. The Waters software program was applied to find optimal collision energy parameters for the signature daughter ions. The concentration of each nucleoside were used to further normalize the levels of mcm^5^U and mcm^5^s^2^U to the levels of m^1^A for each sample, and averaged between two biological replicates of OPCs and two of oligodendrocytes (using the R package Rmisc). Further data visualization and plotting were performed in R using ggplot2.

### APM polyacrylamide gel electrophoresis

RNA resuspended in water was diluted in formamide loading dye and run on gels 6.5% polyacrylamide 19:1, 1X TBE, 8 M urea (0.4 mm × 20 cm × 30 cm) containing 50 ug/mL APM ([(*N*-acryloylamino)phenyl]mercuric chloride, Toronto Research Chemicals, A191380), at 200 V for 14 h in 1X TBE. The samples were then transferred and the blots probed as described above for acid-urea PAGE.

### Data processing of the cortex-purified cells RNA-Seq

FPKM values from^111^ were downloaded from the Gene Expression Omnibus (GEO) repository GSE52564. They were processed using heatmap.2 from the gplots package and ggplot2 in R. Differential expression was assessed with the *limma*-trend approach (linear modelling and eBayes statistics) of the Bioconductor *limma* package, that can be used with CPM values^74^.

### Western blots analysis

OPCs were seeded into 10 cm dishes at 2 million cells per dish for OPCs, 5 million cells per dish in oligodendrocyte media for oligodendrocytes, or 4 million cells per dish in astrocyte media for astrocytes. After 3 days, media was removed and cells were lysed on the plate by adding 400 uL of lysis buffer (50 mM HEPES, pH 7.5, 150 mM KCl, 2 mM EDTA, 1 mM NaF, 0.5% v/v NP40, 0.5 mM DTT, 1X Halt protease inhibitor cocktail (Thermo Fisher scientific, 78430)) per plate, on ice, and scraping the cells off. After transfer to a microfuge tube and incubation on ice for 20 min, the lysate was triturated 12 times through a 25 G needle then cleared at 16,000 × g for 10 min at 4 °C. Protein concentrations were measured by Bradford assay. 20 ug of total protein was run on a 10% polyacrylamide-SDS gel and transferred to PVDF membrane. The blots were blocked with 5% milk in TBS-Tween, and incubated overnight at 4 °C in TBS-Tween + 5% milk with the following antibodies: anti-TYW3 (1:1,000, MyBioSource, MBS150652), anti-ELP1 (1:250, Millipore Sigma, SAB2701068), anti-ELP3 (1:2,000, Abcam, ab190907) or anti-beta-actin (1:5,000, Abcam, ab6276). After three washes in TBS-Tween, they were incubated for 2 h at room temperature with secondary antibodies (anti-Mouse IgG,1:5,000, Abcam, ab216772, or anti-Rabbit IgG, 1:5,000, Abcam, ab216777) in TBS-Tween + 5% milk, washed three times, and the fluorescence signal was read using an Amersham Typhoon 5 laser-scanner platform (Cytiva). Quantitation of the signal was performed with Image Lab (Biorad) software, and the result expressed as a normalization to beta-actin signal, for two biological replicates of each cell type. Further data visualization and plotting were performed using ggplot2 in R^108^.

### Global half-life analysis by 5-EU-Seq

RNA decay analyses were performed in OPCs an oligodendrocytes by an integrated approach combining metabolic labeling with 5-ethynyluridine (5-EU) (Thermo Fisher Scientific, Click-iT nascent RNA capture kit, C10365) and next-generation sequencing library preparation (^112^, see below), as in ref. 5. OPCs were seeded into six 10 cm dishes at 2 million cells each for OPCs, or into five 10 cm dishes at 5 million cells each in oligodendrocyte differentiation media for oligodendrocytes. Cells were pulse-labelled with 0.2 mM of 5-EU after 40 h. After 24 h of incorporation (64 h after seeding), one plate for each cell type was collected as the 0 h time-point, as well as an extra plate for OPCs to use as a control without biotinylation. For the other plates, 5-EU was chased by replacing the media and adding 5 mM of uridine. Cells were collected in 2 mL Trizol at the following time-points: 1 h, 2 h, 4 h and 6 h of chase. RNA was extracted following the Trizol extraction protocol and resuspended in water. 4 ng of in vitro-labelled spike-in was added to 8 μg of each RNA sample, and the samples were treated with 2U of Turbo DNAse (Thermo Fisher Scientific, AM2238) for 30 min at 37 °C. The spike-in mix consisted of partial RNA sequences of *B*.*subtilis* LYSa and firefly luciferase transcripts, of which the gene sequences were cloned into pBluescript SK + plasmid (pJC879 and pJC880, respectively) and transcribed using T7 RNA polymerase (HiScribe kit, NEB E2040S) and 2 mM of 5-EUTP (Abcam, ab146744). After DNAse treatment, NaOAc pH 5.2 was added to a final concentration of 0.3 M and RNA isolated with phenol-chloroform. RNA was precipitated with sodium acetate and 100% ethanol at −20 °C, then spun down at 16,000 × *g* for 30 min at 4 °C, washed twice with 75% ethanol and resuspended in water. Samples were depleted of ribosomal RNAs (rRNA) using Illumina Ribo-Zero Gold rRNA removal kit (MRZG12324), then purified using the RNA Clean and concentrator Kit (R1015, Zymo research). RNA was fragmented by alkaline treatment at 95 °C for 20 min (NaCO3 pH9.2 50 mM final, EDTA 1 mM final). The reaction was stopped on ice by addition of 0.3 M NaOAc pH 5.2, 2 μL glycoblue and 500 μL water. RNA was precipitated with isopropanol, spun at 16,000 × g for 30 min at 4 °C, washed with 75% ethanol and resuspended in 15.75 μL of water. Biotinylation of the 5-EU labelled RNA was performed following the Click-iT nascent RNA capture kit protocol, using 0.5 mM biotin azide (biotin azide was omitted in the control sample), and precipitated at −80 °C overnight. The samples were then purified on Novex denaturing polyacrylamide gels (TBE-urea 15% polyacrylamide, Thermo Fisher Scientific) ran at 200 V, excising RNA between 20 and 70 nucleotides (RNA markers of 26, 34, and 70 nt were used as size control). After extraction from the gel with 500 μL of RNA gel extraction buffer (300 mM NaOAc pH5.2, 1 mM EDTA, 0.25% (w/v) SDS) on a rotator at room temperature overnight, samples were spun through a microfuge spin filter (VWR 29442-752), precipitated with isopropanol, and resuspended in 10 μL of 10 mM Tris-HCL pH 8. Pull-down of the biotinylated RNA was performed following the Click-iT nascent RNA capture kit protocol, using 50 μL of C1 myOne streptavidin beads (Thermo Fisher Scientific) per reaction in a total volume of 300 μL, for 30 min at room temperature, and washed 8 times with 500 μL wash buffer (Click-iT kit, C10365). The beads were then resuspended in 10 μL of wash buffer from the kit and the RNA was dephosphorylated on the beads by adding 10 μL of 10 mM Tris-HCL pH 8 and 20 μL of water, denaturing at 80 °C for 90 s then incubating for 1 h at 37 °C in a thermomixer at 900 rpm, with 5 μL of 10 X PNK reaction buffer, 1 μL SUPERase-In RNase inhibitor (Thermo Fisher Scientific, AM2694) and 1 μL of T4 polynucleotide kinase (NEB, M0201S). The PNK was inhibited by incubating at 70 °C for 10 min, and the sample washed once with 500 μL Wash buffer 2 from the Click-iT kit before resuspending in 9 μL of 10 mM Tris-HCl pH8. A preadenylylated linker (1 μL of 0.5 μg/μL) (Universal miRNA Cloning Linker, NEB, S1315S) was then ligated after a brief denaturation of 90 s at 80 °C, with T4 truncated RNA ligase 2 (T4 Rnl2(tr), M0242L, NEB), for 2 h at 24 °C then overnight at 16 °C, shaking at 900 rpm to avoid the beads from pelleting down. The next day, the samples were washed with 500 μL wash buffer 1 then 500 μL wash buffer 2 and resuspended in 10 μL of 10 mM Tris-HCl pH8. Reverse transcription was performed on the beads with 2 μL of 1.25 μM of reverse transcription primer oJC3453^112^ and Superscript III, incubating at 50 °C at 900 rpm for 1 h. The cDNA was then eluted from the beads by heating at 90 °C for 8 min, mixing at 1200 rpm, and immediately collected from the beads. After isopropanol precipitation, the cDNAs were purified on a 15% denaturing polyacrylamide gel as described above, excising the product above the unextended reverse transcription primer. After extraction from the gel overnight (400 μL of DNA gel extraction buffer: 300 mM NaCl, 10 mM Tris-HCl pH8, 1 mM EDTA) and isopropanol precipitation, the cDNAs were circularized with CircLigase (Epicenter, CL4115K) for 1 h at 60 °C. A tenth of the circularized cDNA was used for each 20 μL PCR amplification reaction (0.2 μL of Phusion polymerase (NEB, M0530L), 0.2 mM dNTPs and 0.5 μM forward and reverse library primers (different indexing reverse primers to multiplex samples, see Supplementary Table 1). After checking the libraries obtained at 12, 14, 16 cycles (1 min initial denaturation at 98 °C, then cycles of 20 s at 98 °C, 20 s at 65 °C and 20 s at 72 °C), the rest of the cDNA was used in 7 individual PCR reactions of 14 or 16 cycles, and separated by 8% non-denaturing PAGE, then excised from the gel (avoiding any lower product band derived from unextended reverse transcription primer). The libraries corresponding to the same sample were pooled together in a total of 15 μL of 10 mM Tris-HCl pH 8, and sequenced on an Illumina HiSeq 2500 using single read 76 cycles runs.

### Reporter assay

The EF.CMV.RFP construct, a dual EF1a/CMV promoter plasmid expressing RFP under the CMV promoter, was used to make dual GFP/RFP reporters. EF.CMV.RFP was a gift from Linzhao Cheng obtained from Addgene (Addgene plasmid # 17619; http://n2t.net/addgene:17619; RRID:Addgene_17619)^113^. This plasmid was linearized with EcoRV. Gene blocks (IDT) of eGFP sequence (oJC4794), eGFP with synonymous substitutions of Lys and Phe codons (oJC4795) and eGFP with synonymous substitutions to all possible oligodendrocyte non-optimal codons (oJC4803) sequences were designed with overhangs for insertion after EF1a promoter at EcoRV restriction site in EF.CMV.RFP. These were inserted into EF.CMV.RFP by Gibson assembly, using 0.05 pmol of vector and 0.1 pmol of each insert oJC4794, oJC4795 and oJC4803, to give plasmids pJC1191, pJC1192 and pJC1196, respectively (Supplementary Table 1). 5 μg of each plasmid were electroporated into 5 million OPCs, which were then plated into six-well plates at 1 million cells/plate, and grown in oligodendrocyte differentiation media. GFP and RFP fluorescence were checked by microscopy and cells collected into Trizol after 3 days. RNA was extracted as described above, DNase treated, and 0.5 μg was reverse transcribed with Superscript III. The cDNA was diluted 50 times and 8 μL was used for every 20 μL reaction with 200 nM of each primer and 1X TB Green Advantage qPCR premix (639676, Takara Bio). Amplification signal was acquired with a StepOne real-time PCR system instrument (Applied Biosciences). For each primer pair (RFP and 5′UTR of eGFP), a standard curve was obtained with 4 samples of a tenfold serial dilution ranging from 1/20 to 1/20,000 of a control cDNA, and used to get the absolute values of each test sample. Relative expression levels between the different GFP reporters were inferred from the ratio of absolute values first normalized to the values of the internal control RFP. Each reaction was performed in technical duplicates or triplicates (and negative control included RFP-only expressing plasmid).

### Ribosome profiling and RNA-Seq experiments

Ribosome profiling and parallel RNA-Seq were performed similarly to^112^. OPCs were seeded into six 15 cm dishes at 5 million cells each for OPCs, or into six 15 cm dishes at 12.5 million cells each in oligodendrocyte differentiation media for oligodendrocytes. After 3 days, the media was removed and the cells flash-frozen over a thin layer of liquid nitrogen. The cells were scraped off on ice in 2X lysis buffer (20 mM Tris-HCl pH7.5, 10 mM MgCl2, 200 mM KCl, 2% Triton X-100, 2 mM DTT, 100 ug/mL emetine (Abcam, ab141478), 1000 U/mL RNAsin (Promega, N2111)), pooling the different plates’ lysates from the same cell type. The lysates were sheared ten times with a 22-gauge needle then ten times with a 25-gauge needle, then cleared from nuclei by spinning at 20,000 × g for 10 min at 4 °C with a TLA120.2 rotor. For total RNA-Seq, an aliquot of this input lysate was directly precipitated without nuclease digestion. Different nucleases have been used to generate ribosome-protected fragments^81,114^. Treatments with RNase I, even for a very short period of time, led to an important loss of the monosomes peak, presumably due to rRNA degradation, while MNase treatment preserved the monosome integrity (Supplementary Fig. 7a). To generate the footprints samples, 3 OD_260_ were thus treated with 360 units of MNase (Roche/Sigma, 10107921001) with 5 mM CaCl2 on a nutator, at room temperature for 30 min, then quenched with 10 mM EGTA and 5 uL SUPERase-In. The resulting lysates were loaded onto 15-45% (w/w) sucrose density gradients generated by a Biocomp Gradient Master in gradient buffer (in 50 mM Tris-Acetate pH 7, 50 mM NH_4_Cl, 12 mM MgCl_2_, 1 mM DTT), and centrifuged in SW41Ti rotor for 2 h and 26 min at 41,000 rpm (288,000 × g) and 4 °C. Fractionation was performed with a Brandel Fractionation System and an Isco UA-6 ultraviolet detector. The monosome fractions were precipitated overnight at −80 °C using 2 volumes 95% ethanol, and both monosome and total input samples were pelleted at 16,000 × g for 30 min at 4 °C and washed with 75% ethanol. RNA was isolated by two phenol chloroform extractions and precipitated with one-tenth volume of 7.5 M ammonium acetate and 2 volumes 95% ethanol. RNA pellets were recovered by centrifugation at 16,000 x *g* for 30 min. Total RNA samples for RNA-Seq were treated with 2U of DNAse I at 37 °C for 30 min, then phenol chloroform extracted and precipitated. 5 μg of DNase treated total RNA or footprint RNA were depleted of rRNAs, then purified using the RNA Clean and concentrator Kit. Total RNA samples were fragmented by alkaline treatment to obtain RNA sizes similar to the footprints samples, at 95 °C for 35 min, and the reaction was stopped on ice by addition of 0.3 M NaOAc pH 5.2, 2 μL glycoblue and 500 μL water before precipitation with isopropanol. The next steps were similar to the decay RNA library preparation. The samples were purified by TBE-urea 15% PAGE and RNA excised between ~26 and 40 nucleotides (RNA oligomers of 26 and 34 nt were used as size markers). After extraction from the gel, RNA was precipitated with isopropanol and resuspended in 10 mM Tris-HCL pH 8. After dephosphorylation of the RNA with T4 polynucleotide kinase and precipitation, 0.5 μg of preadenylylated linker was ligated with T4 truncated RNA ligase 2. The ligated RNAs were purified by TBE-urea 15% PAGE and excised from the gel as above. Reverse transcription was performed with reverse transcription primer oJC3453 and Superscript III. After RNA hydrolysis with 0.1 N NaOH for 20’ at 98 °C, the cDNAs were precipitated and gel purified. They were circularized with CircLigase, and PCR amplified using Phusion polymerase and the forward and reverse library primers selected for each sample (see Supplementary Table 1). The libraries from the 12 cycles PCR reactions were separated and excised from 8% non-denaturing PAGE, then sequenced on an Illumina HiSeq 2500 using single read 50 cycles runs.

### 5-EU-seq and ribosome profiling reads processing

All library QC, multiplexing, and sequencing was carried out by the Genomics Core Facility of the CWRU School of Medicine’s Genetics and Genome Sciences Department. The raw reads quality was assessed with FastQC (http://www.bioinformatics.babraham.ac.uk/projects/fastqc/). Reads were trimmed off the adapter with fastx_clipper (FASTX-Toolkit, http://hannonlab.cshl.edu/fastx_toolkit/) with the options -a CTGTAGGCACCATCAAT -l 18 -n -c -v. Reads were mapped to GRCm38 (mm10) genome using hisat2 v2.1.0^115^ and default parameters with—dta option, then converted into bam format and indexed using samtools v.1.7–2. Sequence annotation gff3 files were generated for the mouse canonical transcripts and coding sequence (CDS) from Gencode vM9 (GRCm38) using an R script from^81^ (https://github.com/rasilab/adarnell_2018). These files included only coding transcripts to filter out rRNA reads. For decay and ribosome density analyses, FPKM were tabulated using stringtie v.1.3.5 and ballgown^116^, annotating reads to transcripts for RNA-Seq and to CDS for ribosome footprints.

### mRNA half-lives and codon to stability correlation coefficient calculation

The raw FPKM numbers at each time-point were normalized to the relative number of reads aligning to the spike-ins (average of reads aligning to Luc and LYSa normalized to the number of total reads). Transcripts with <1 FPKM at time-point 0 and with 0 FPKM at time-point 1 and 2 h were filtered out. Spike-in-normalized FPKM values for each timepoint were further normalized to the 0 h value, and half-lives were estimated by fitting a least absolute deviations regression model to an exponential decay equation as in ref. 6, using solver in excel. Estimated half-lives longer than 6 h were set up at ≥6 h as the experiment didn’t include further time-points. Further data visualization and plotting were performed using ggplot2 in R^108^.

The CDSs of the canonical mouse transcripts were filtered to keep sequences starting with “ATG”, and half-life datasets were filtered to obtain half-lives > 0. Codon occurrences for each sequence were obtained using the oligonucleotideFrequency function in the Biostrings R package^117^ with width = 3, step = 3, as.prob = TRUE to normalize to transcript length. CSCs were calculated as the Pearson correlation coefficient between transcript half-life and codon frequency for all 61 non-stop codons using the cor.test function in the R stats package with method = “pearson” (Supplementary Software).

### Ribosome profiling quality controls and pausing scores

Most quality controls (read counts mapping to rRNA, read length distribution, read density around start and stop codons) were performed and plotted in R as in ref. 81 (https://github.com/rasilab/adarnell_2018). The fraction of reads mapping to rRNA was inferred using bowtie2 and a fasta file of mouse rRNA sequences (mm10) downloaded from NCBI. Footprints lengths were calculated from the bam files using the function readGAlignments in GenomicAlignments package^118^. Read density around the start and stop codons were inferred using the function mapToTranscripts from the R GenomicFeatures package^118^ and our annotation file for mouse canonical transcripts (see above). To get the fraction of reads mapping to CDS or UTRs, we used the Rsubread package’s featureCounts function^105^ and normalized the coverage on each feature by the feature length. Finally, the read density over each coding frame was calculated and plotted after estimation of the P-site offset from the 3′ end of reads using the R package ribosomeProfilingQC^119^.

Pause scores were calculated as in ref. 81 (https://github.com/rasilab/adarnell_2018) with some minor adaptations. In brief, footprints were trimmed (9 nucleotides from 5′ and 12 nucleotides from 3′) to smooth ribosome density profiles and the reads coverage estimated at each position over canonical CDS. This was normalized to the mean read count for each CDS, filtering out CDS with read densities lower than 1 read per codon. Codons sequences were extracted for each CDS and the normalized read density was computed for each codon in a 50 nucleotides window on either side of all occurrences of that codon. The sum of this 100 nucleotide ribosome density values was then computed for each codon. The values were normalized to the same coverage over each codon window calculated for the total RNA-seq reads processed the same way, to account for position biases in library amplification. Pause scores for biological replicates (two OPCs, two oligodendrocytes) were averaged using the R package Rmisc.

### Gene ontology over-representation analyses

Lists of genes identifiers from the bins of lowest UUU and AAA codon bias were uploaded into Panther Classification system (http://pantherdb.org/)^120,121^. A statistical overrepresentation test was performed over the reference list of all the genes expressed in oligodendrocytes in the decay RNA-Seq, using Fisher’s exact test and False Discovery Rate correction, and the Gene Ontology cellular component annotation dataset^122^.

### Statistical analyses and reproducibility

Statistical tests, replicate descriptions and *p* values are specified in Fig. legends. Data are typically graphed as replicate data points or/and mean ± standard error of the mean (SEM) as detailed in the Fig. legend. The function summarySE of the R package Rmisc was used to calculate the mean and SEM from biological replicates data.

Three biological replicates of OPCs and two biological replicates of oligodendrocytes (differentiated from two of the OPCs replicates), and two biological replicates of WT and *tyw1∆* yeast, were used for tRNA sequencing. Two OPCs and oligodendrocytes replicates were used for the LC-MS/MS analyses. For the large-scale mRNA half-life analysis, three biological replicates of OPCs and two biological replicates of oligodendrocytes (that match two of the OPC replicates) were used. Two OPC replicates and the corresponding differentiated oligodendrocytes generated the ribosome footprints and paralleled RNA-seq samples. DESeq2 analyses performed for the differential expression of modification enzymes in our OPCs and differentiated oligodendrocytes included the RNA-Seq data that matches the ribosome footprints as well as the 0 h time-point from the decay experiment, hence a final pool of five biological replicates for OPCs and four biological replicates for oligodendrocytes.

The RT-PCR from Fig. 1c was repeated three times independently with similar results. The quantified Phe-GAA and Lys-UUU Northern blot analyses from Fig. 2h–i were repeated four times independently with similar results, and Fig. 3a, b were reproduced similarly on three different PAGE using three biological replicates. The HCl experiment in Fig. 3c, e is from two biological replicates of OPCs, oligodendrocytes, and astrocytes. The quantitation of Supplementary Fig. 2 was obtained from independent PAGE of biological replicates, with the exact n indicated in the Fig. The Northern blots in Supplementary Fig. 5b and c were repeated twice independently with similar results. Supplementary Fig. 5d was reproduced independently twice for Glu, Gln and Arg tRNAs.

All statistical tools and softwares are publicly available and listed in the Methods. The R packages dplyr (Kruskal–Wallis)^123^ and ggpubr^124^ were used to assess significance unless it was evaluated as part of an analysis tool (DEseq2, DEXseq, *limma* and Panther). A *p* value < 0.05 was considered significant unless otherwise noted.

### Reporting summary

Further information on research design is available in the Nature Research Reporting Summary linked to this article.

## Supplementary information

Supplementary Information

Description of Additional Supplementary Files

Supplementary Software 1

Reporting Summary

## Data Availability

All datasets generated in this study (QuantM-seq, Decay-Seq, Ribosome profiling) have been deposited in Gene Expression Omnibus (https://www.ncbi.nlm.nih.gov/geo/) under the SuperSeries accession code GSE182811. These include raw Fastq files as well as processed data. The expression dataset from myelinating oligodendrocytes, newly formed oligodendrocytes, whole cortex, OPCs and astrocytes are available in the GEO datatbase under accession code GSE52564. Other datasets are publicly available: GtRNAdb high confidence list of tRNAs for mouse (GRCm38) (http://gtrnadb.ucsc.edu/genomes/eukaryota/Mmusc10/Mmusc10-gene-list.html) and yeast (S288c) (http://gtrnadb.ucsc.edu/genomes/eukaryota/Scere3/Scere3-gene-list.html), hisat2 genome index (GRCm38 genome_tran, https://cloud.biohpc.swmed.edu/index.php/s/grcm38_tran/download), gff3 files for mouse canonical transcripts and coding sequence from Gencode vM9 (https://www.gencodegenes.org/mouse/release_M9.html), and mouse ribosomal RNA sequences from NCBI (https://www.ncbi.nlm.nih.gov/nuccore/). Source data are provided with this paper.

## Source data

Source data

## Code availability

All code and softwares are previously published and publicly available. QuantM-seq analysis code is accessible in Pinkard et al^13^. as Supplementary software. mRNA decay analyses are described in Methods as well as in referenced papers^5,6^. Ribosome profiling analyses tools and code are described in Methods and referenced^81,119^ (https://github.com/rasilab/adarnell_2018). Data processing and visualization tools are all available as R packages and described in Methods^124^. Code files describing QuantM-seq, Decay-seq and ribosome profiling processing and plotting are provided as Supplementary Software 1.
