# Supplementary material for: Oligodendrocyte differentiation alters tRNA modifications and codon optimality-mediated mRNA decay
Source: Nat Commun. 2022 Aug 25;13:5003. doi: 10.1038/s41467-022-32766-3 (PMC9411196; doi:10.1038/s41467-022-32766-3)
Supplement: Supplementary file 1 — Supplementary Information [file 41467_2022_32766_MOESM1_ESM.pdf]

# Oligodendrocyte differentiation alters tRNA modifications and codon optimality-mediated mRNA decay

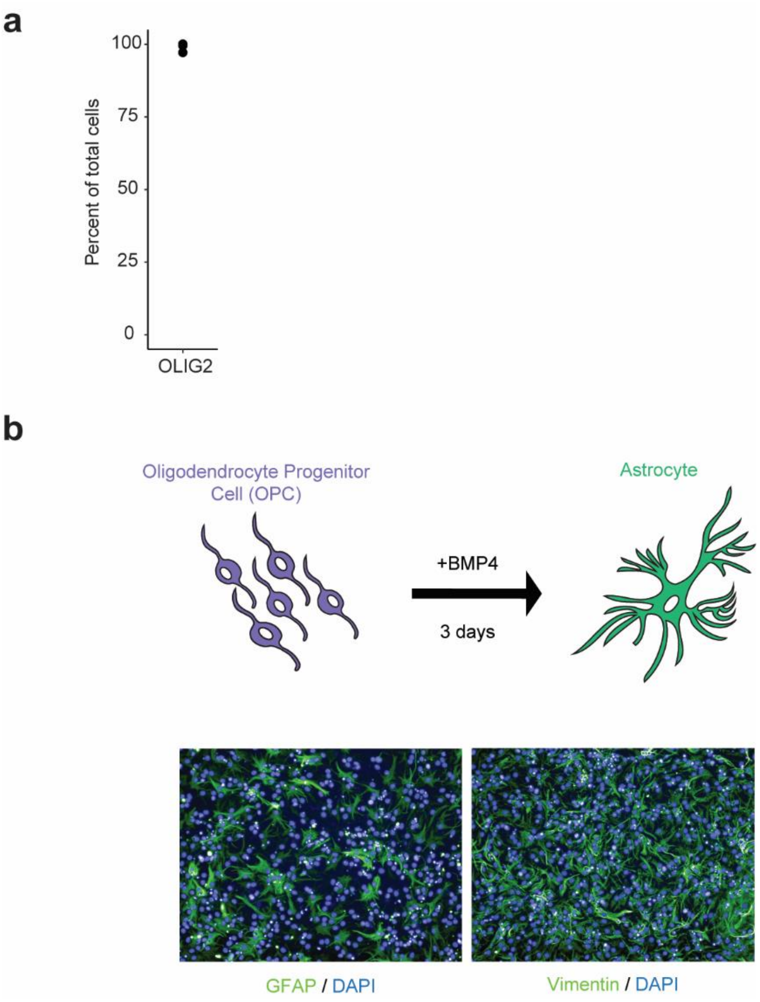


**Supplementary Fig. 1 - Related to Fig. 1. Characterization of the cell culture models**

**a** Percent of cells derived from mouse pluripotent stem cells (epiblast) that express the oligodendrocyte lineage marker OLIG2 (n=3 separate wells).

**b** Model of differentiation of OPCs to astrocytes. At 3 days after addition of Bone Morphogenetic Protein 4 (BMP4), the markers of mature astrocytes GFAP and Vimentin are detected by immunocytochemistry.

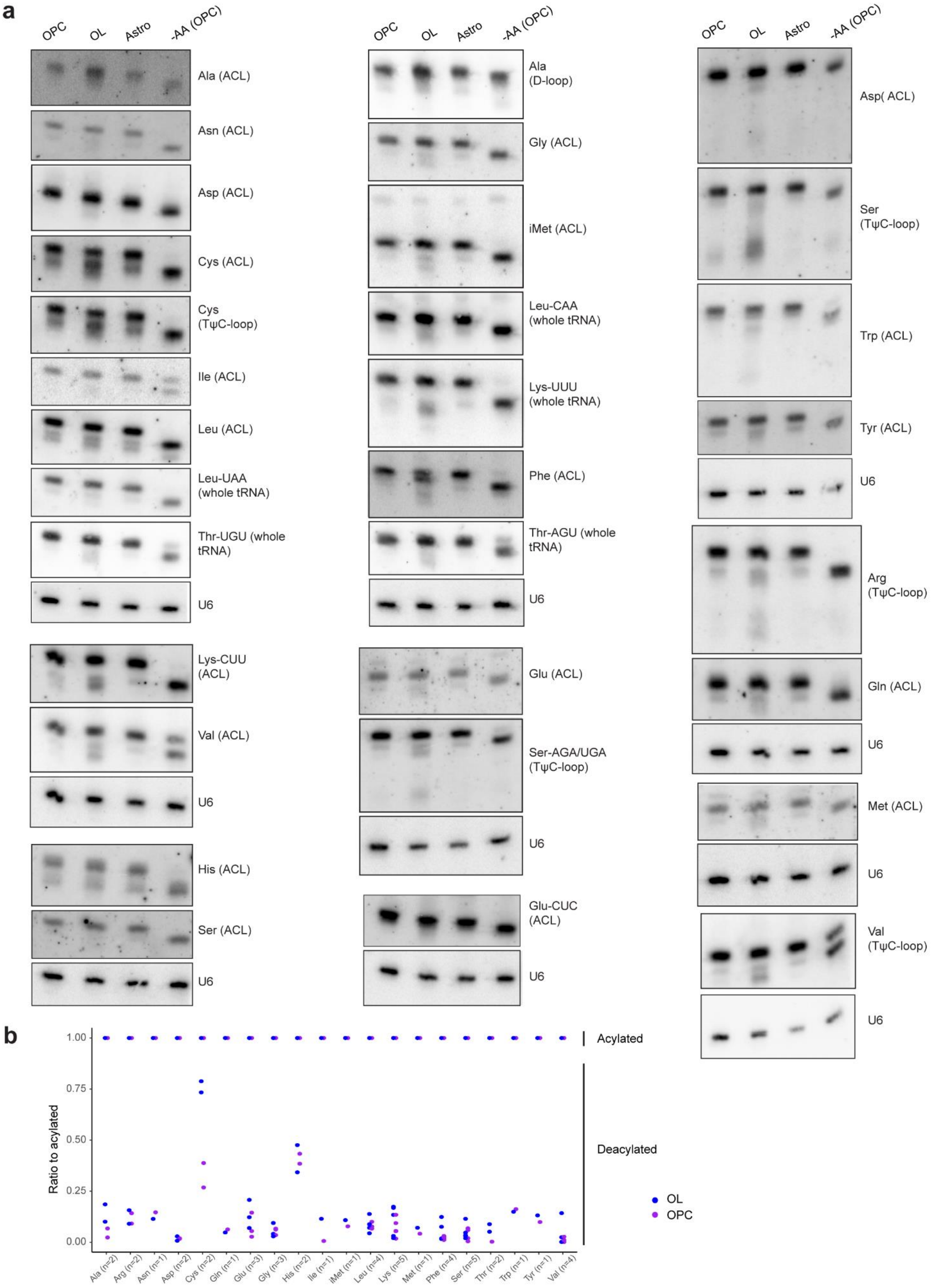
a
OPC
OL
Astro
-AA (OPC)
Ala (ACL)
Asn (ACL)
Asp (ACL)
Cys (ACL)
Cys (TψC-loop)
Ile (ACL)
Leu (ACL)
Leu-UAA (whole tRNA)
Thr-UGU (whole tRNA)
U6
Lys-CUU (ACL)
Val (ACL)
U6
His (ACL)
Ser (ACL)
U6
Ala (D-loop)
Gly (ACL)
iMet (ACL)
Leu-CAA (whole tRNA)
Lys-UUU (whole tRNA)
Phe (ACL)
Thr-AGU (whole tRNA)
U6
Glu (ACL)
Ser-AGA/UGA (TψC-loop)
U6
Glu-CUC (ACL)
U6
Asp( ACL)
Ser (TψC-loop)
Trp (ACL)
Tyr (ACL)
U6
Arg (TψC-loop)
Gln (ACL)
U6
Met (ACL)
U6
Val (TψC-loop)
U6
b
Ratio to acylated
1.00
0.75
0.50
0.25
0.00
Acylated
Deacylated
OL
OPC
Ala (n=2)
Arg (n=2)
Asn (n=1)
Asp (n=2)
Cys (n=2)
Gln (n=1)
Glu (n=3)
Gly (n=3)
His (n=2)
Ile (n=1)
iMet (n=1)
Leu (n=4)
Lys (n=5)
Met (n=1)
Phe (n=4)
Ser (n=5)
Thr (n=2)
Trp (n=1)
Tyr (n=1)
Val (n=4)

**Supplementary Fig. 2 - Related to Fig. 1. tRNA charging status between OPCs and oligodendrocytes**

**a** Acid-urea PAGE and Northern Blot analysis of tRNAs in OPCs, oligodendrocytes and astrocytes. tRNAs were detected by radiolabelled probes complementary to sequences in the anticodon loop (ACL), D-loop, TψC-loop or the whole tRNA sequence. A control of deacylation (OPC tRNA submitted to alkaline hydrolysis for 40 min) is included (-AA, - Amino Acid). U6 snRNA is probed as a loading control.

**b** Ratio of deacylated tRNA over acylated tRNA in OPCs and oligodendrocytes (OL), as quantified from Northern blot signal (n= number of biologically independent experiments).

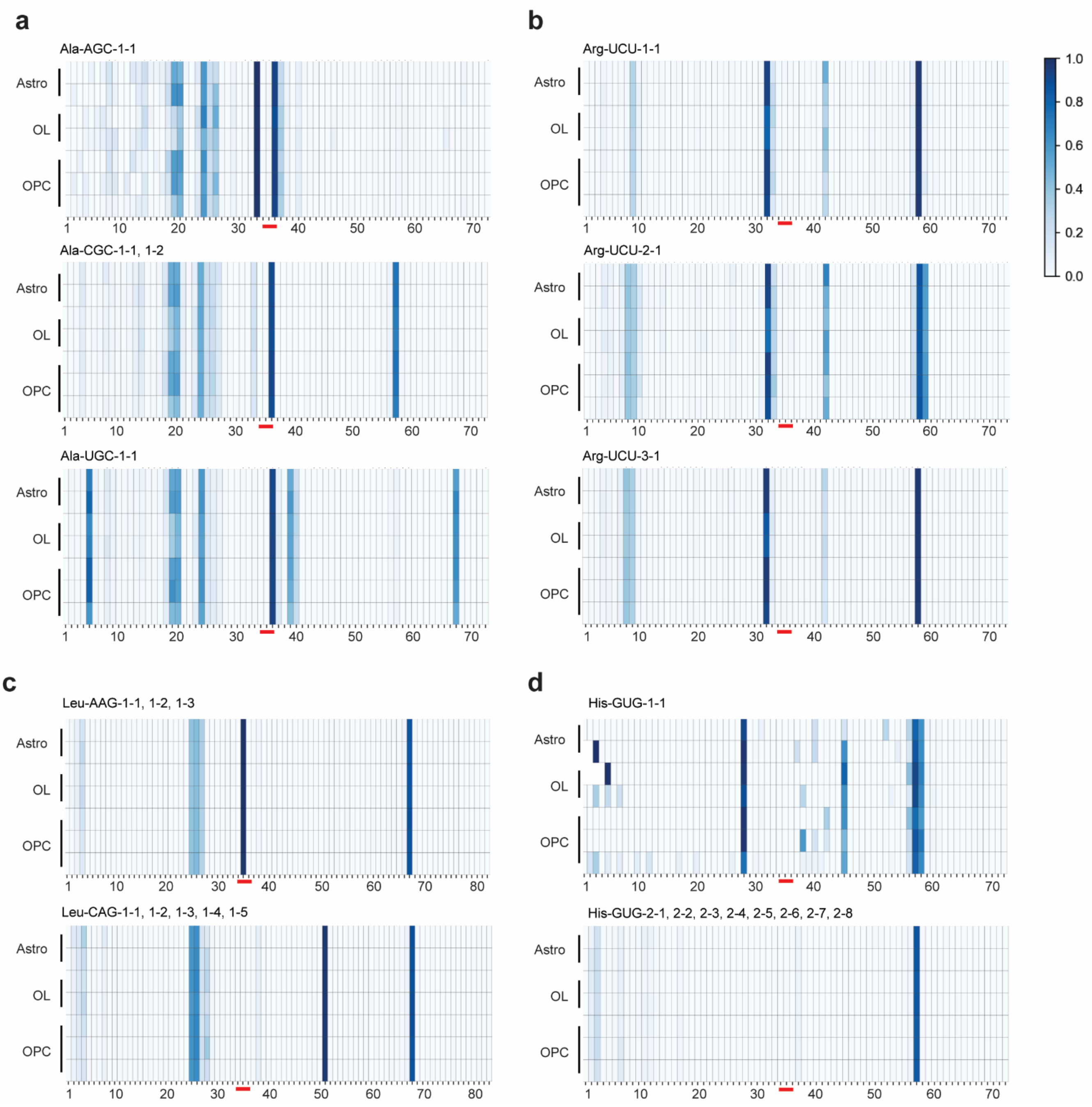


**Supplementary Fig. 3 - Related to Fig. 2. tRNA sequencing distinctions between OPCs and oligodendrocytes**

Heatmaps for different Ala tRNAs (**a**), Arg-UCU isodecoders (**b**), Leu tRNAs (**c**) and His-GUG isodecoders (**d**). Each represents sequencing variant fractions at each tRNA position (*x*-axis) across OPCs (3 biological replicates), oligodendrocytes (OL, 2 biological replicates) and astrocytes (Astro, 2 biological replicates) (*y*-axis). The numbers below the plot indicate

nucleotide position, the red line shows the anticodon position (nucleotides 34-36). The color bar indicates the color scale of the frequency of variants.

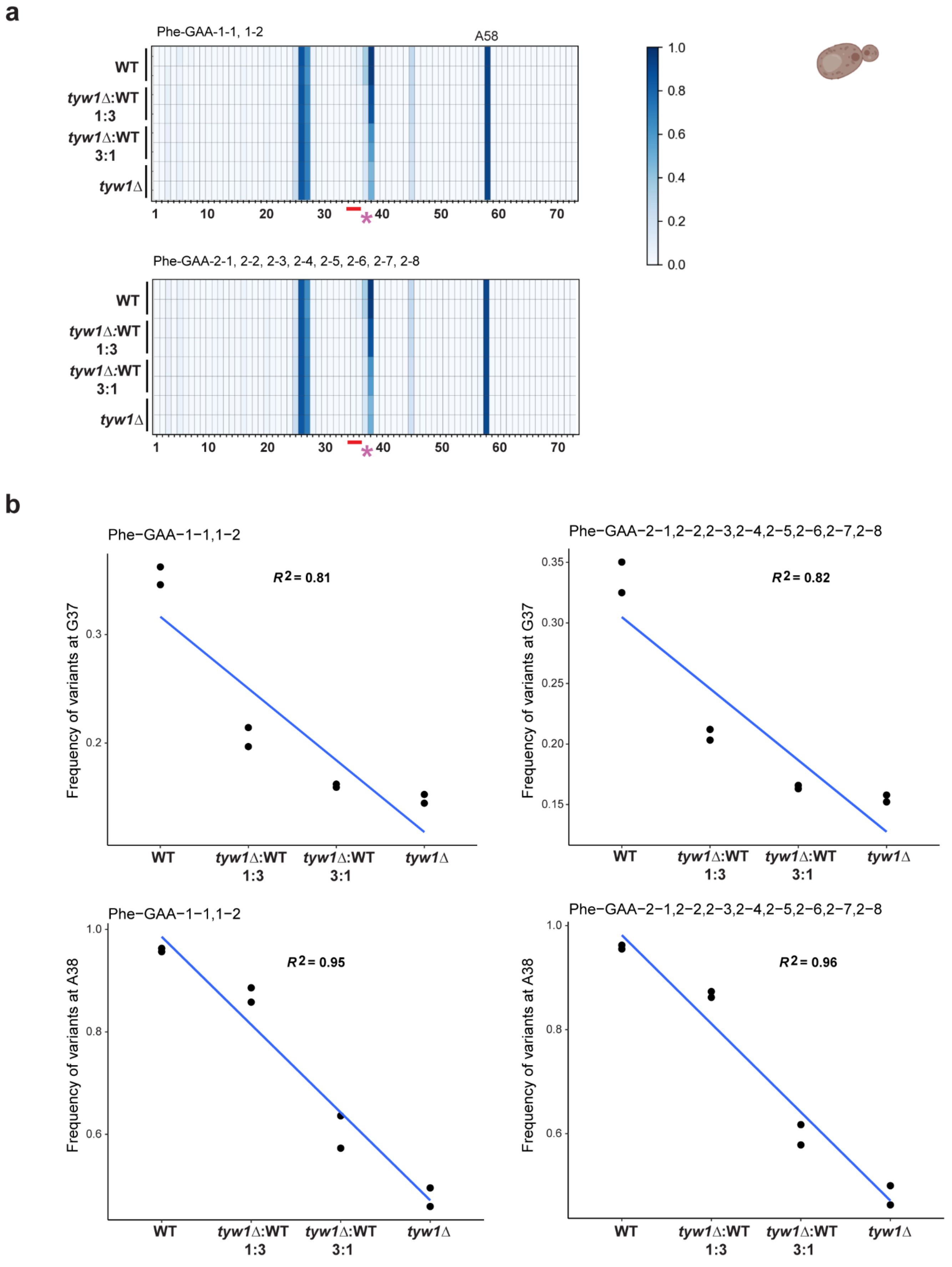
a
Phe-GAA-1-1, 1-2
A58
WT
tyw1Δ:WT 1:3
tyw1Δ:WT 3:1
tyw1Δ
1 10 20 30 40 50 60 70
1.0
0.8
0.6
0.4
0.2
0.0
Phe-GAA-2-1, 2-2, 2-3, 2-4, 2-5, 2-6, 2-7, 2-8
WT
tyw1Δ:WT 1:3
tyw1Δ:WT 3:1
tyw1Δ
1 10 20 30 40 50 60 70
b
Phe−GAA−1−1,1−2
Frequency of variants at G37
R2 = 0.81
0.3
0.2
WT
tyw1Δ:WT 1:3
tyw1Δ:WT 3:1
tyw1Δ
Phe−GAA−2−1,2−2,2−3,2−4,2−5,2−6,2−7,2−8
Frequency of variants at G37
R2 = 0.82
0.35
0.30
0.25
0.20
0.15
WT
tyw1Δ:WT 1:3
tyw1Δ:WT 3:1
tyw1Δ
Phe−GAA−1−1,1−2
Frequency of variants at A38
R2 = 0.95
1.0
0.8
0.6
WT
tyw1Δ:WT 1:3
tyw1Δ:WT 3:1
tyw1Δ
Phe−GAA−2−1,2−2,2−3,2−4,2−5,2−6,2−7,2−8
Frequency of variants at A38
R2 = 0.96
1.0
0.8
0.6
WT
tyw1Δ:WT 1:3
tyw1Δ:WT 3:1
tyw1Δ

**Supplementary Fig. 4 - Related to Fig. 2. QuantM-seq to estimate differences in tRNA modifications**

**a** Heatmaps for the different isodecoders of yeast Phe-GAA tRNA, representing sequencing variant fractions at each tRNA position (*x*-axis) across WT yeast, *TYW1* deletion (*tyw1*Δ*,* lacking wybutosine at G37*)* and mixes of *tyw1*Δ and WT RNA (ratio 1:3 and 3:1) (each has 2 biological replicates) (*y*-axis). The numbers below the plots indicate nucleotide position, the red line shows the anticodon position (nucleotides 34-36), and the asterisk where the variance to the reference is observed (nucleotide 37-38). The color bar indicates the color scale of the variant frequency.

**b** Dot plot of the fraction of variants at position G37 and A38 of Phe-GAA tRNA (isodecoder 1 family, top, and isodecoder 2 family, bottom), in WT yeast, *TYW1* deletion, and mixes of *tyw1*Δ and WT RNA (ratio 1:3 and 3:1) (each has 2 biological replicates). Blue line: linear regression model.

*Budding yeast illustration created with BioRender.com.*

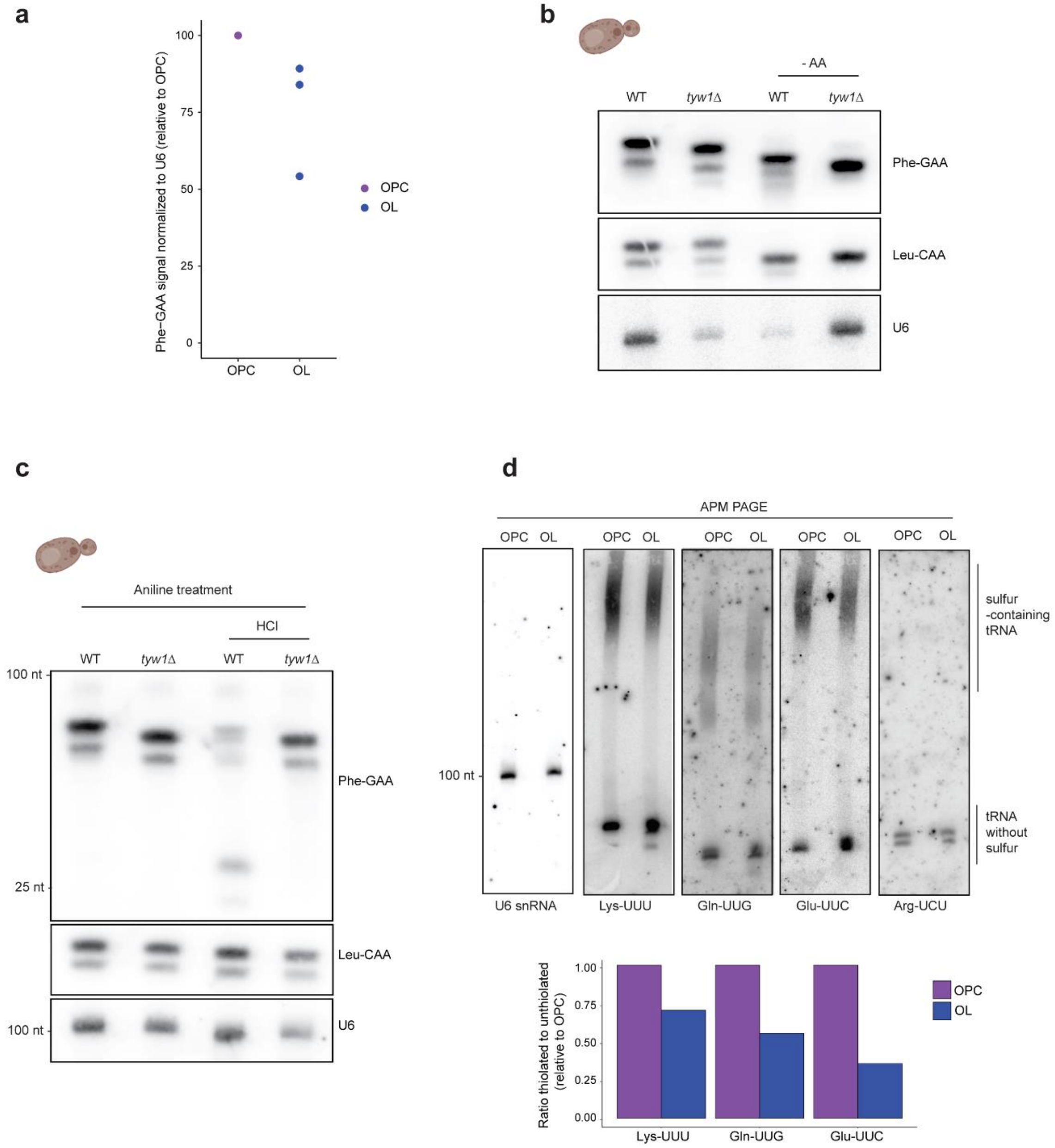


**Supplementary Fig. 5 - Related to Fig. 3. Investigation of tRNA modifications by Northern blot**

**a** Quantitation of Phe-GAA tRNA signal from 3 different Northern blots, normalized to the signal of the snRNA U6 used as a loading control, and expressed relative to the signal in OPCs.

**b** Acid-urea PAGE and Northern blot analysis of WT and *TYW1* deletion yeast RNA, with or without alkaline hydrolysis (-AA, deacylation). Phe-GAA and Leu-CAA were detected with a full-length tRNA complementary probe. U6 snRNA is used as a loading control.
**c** Acid-urea PAGE and Northern blot analysis of WT and *TYW1* deletion yeast RNA, submitted to aniline treatment with or without acidic hydrolysis (HCl). Phe-GAA and Leu-CAA were detected with a full-length tRNA complementary probe. U6 snRNA is used as a loading control. nt: nucleotides, molecular weight estimated from bromophenol blue and xylene cyanol signals.

**d** APM/PAGE and Northern blot analysis of OPCs and oligodendrocytes (OL). Lys-UUU tRNA, Gln-UUG and Glu-UUC were detected with a full-length tRNA complementary probe, and Arg-UCU with a probe complementary to the TψC-loop. The bar chart is the quantitation of the top band (tRNA with $s^2$ modification) to the lower band (without $s^2$ modification) for Lys-UUU, Gln-UUG and Glu-UUC tRNAs in both cell types, relative to OPCs. U6 was used as a loading control as well as a control of RNA without $s^2$ modification. nt: nucleotides, molecular weight estimated from xylene cyanol dye signal.

*Budding yeast illustration created with BioRender.com.*

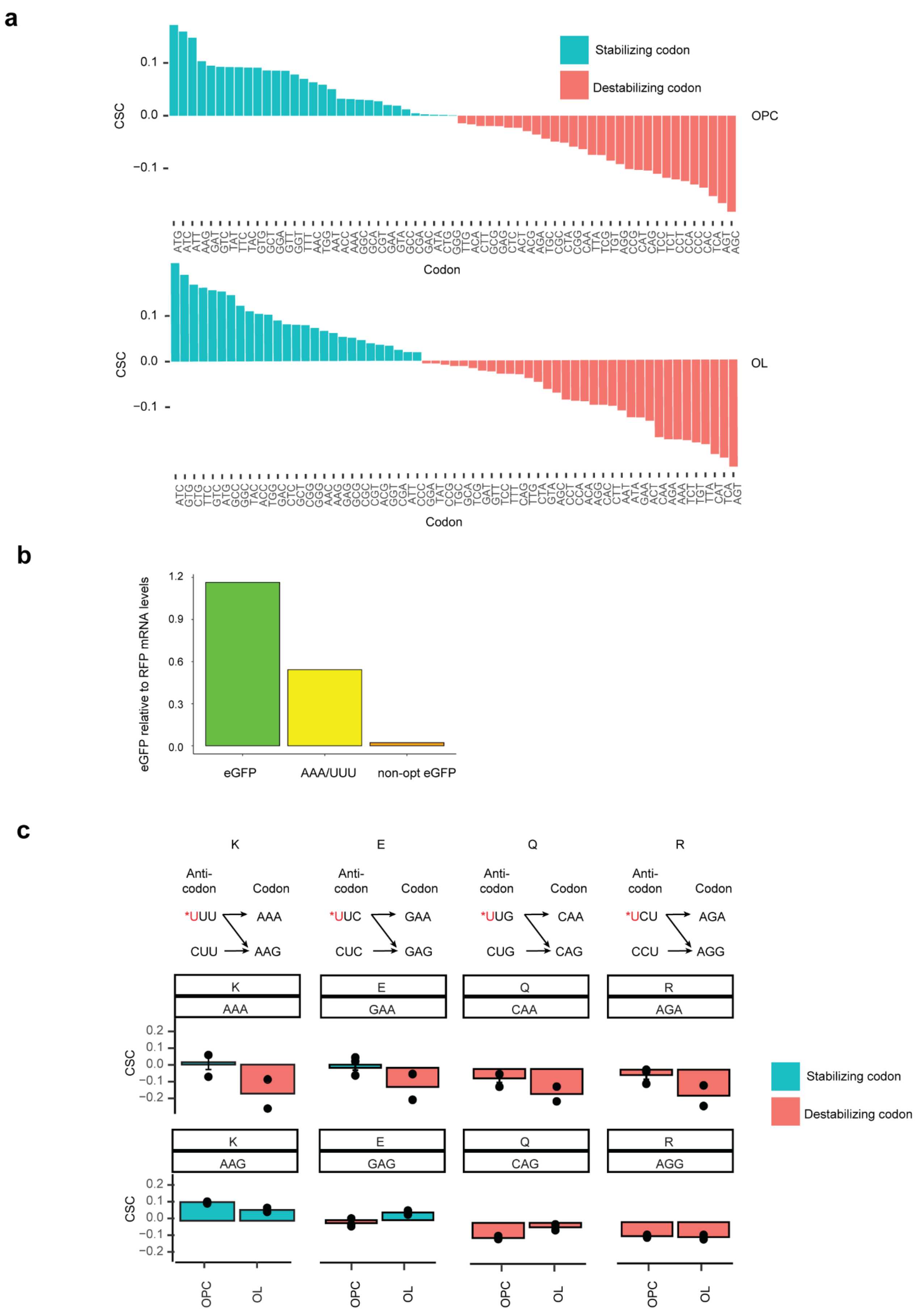
a
CSC
0.1
0.0
−0.1
Stabilizing codon
Destabilizing codon
OPC
Codon
OL
b
eGFP relative to RFP mRNA levels
1.2
0.9
0.6
0.3
0.0
eGFP
AAA/UUU
non-opt eGFP
c
K
E
Q
R
Anti-codon
Codon
*UUU AAA
CUU AAG
*UUC GAA
CUC GAG
*UUG CAA
CUG CAG
*UCU AGA
CCU AGG
K AAA
E GAA
Q CAA
R AGA
K AAG
E GAG
Q CAG
R AGG
0.2
0.1
0.0
−0.1
−0.2
OPC
OL

**Supplementary Fig. 6 - Related to Fig. 5. Effect of codon identity on mRNA stability in oligodendrocytes**

**a** Codon Stabilization Coefficient (CSC) plotted for each codon in OPCs and oligodendrocytes. Tile color represents codons enriched in stable transcripts, and pink represents codons enriched in unstable transcripts.

**b** Expression levels of eGFP mRNA relative to RFP mRNA (RT-qPCR), in oligodendrocytes differentiated from OPCs after electroporation of the following reporters: eGFP: optimal reporter (normal eGFP sequence); AAA/UUU: optimal eGFP with AAG and UUC codons replaced by AAA and UUU, respectively; non-opt eGFP: non-optimal reporter with all optimal codons replaced by non-optimal.

**c** CSC values for the synonymous codons of lysine, glutamate, glutamine and the 2 arginine codons that are decoded by a tRNA bearing a $mcm^5s^2$ modification on $U_{34}$ in the anticodon, in OPCs and oligodendrocytes. Represented are the values of 3 biological replicates in OPCs and 2 biological replicates in oligodendrocytes (dots), with the mean superposed as a bar (mean +/- SEM for OPCs). Stabilizing codon: codon with a positive CSC value; destabilizing codon: codon with a negative CSC value. Above is a schematic of the tRNA anticodon that can decode each codon, the asterisk and red letter represent $mcm^5s^2U_{34}$.

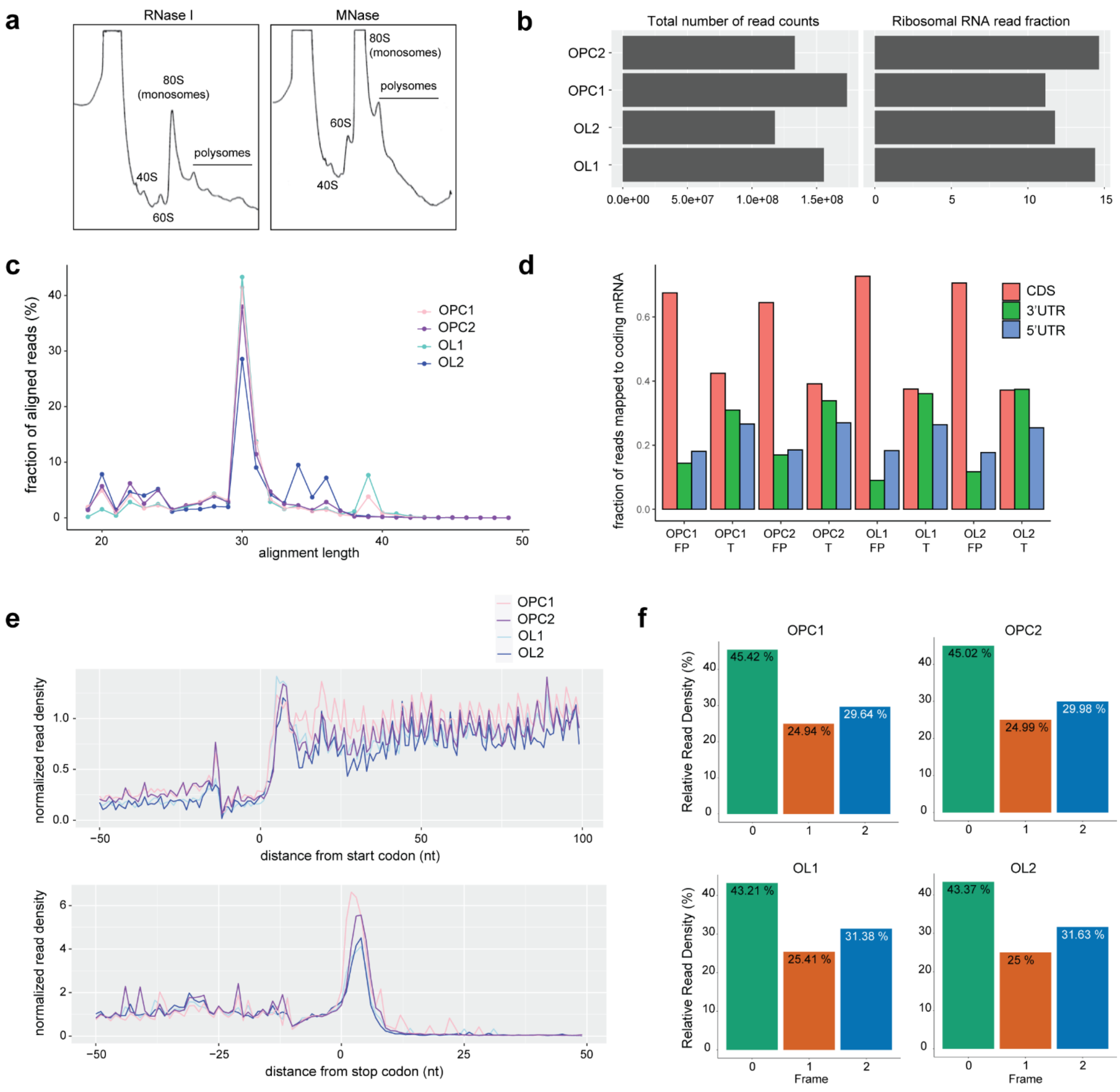


**Supplementary Fig. 7 - Related to Fig. 5. Ribosome profiling quality controls**

**a** Polyribosome traces from extracts of OPCs treated with RNase I or MNase (micrococcal nuclease).

**b** Total number of reads and fraction of reads mapping to ribosomal RNA in ribosome footprint samples from OPCs and oligodendrocytes (OL) replicates. 1 and 2 are the replicate number.

**c** Read length distribution of the ribosome footprints from the two replicates of OPCs and oligodendrocytes (OL).
**d** Fraction of reads mapping to coding sequences (CDS) and UTRs in each replicate of OPCs and oligodendrocytes (OL) footprints samples (FP) or total input samples not treated with nuclease (T). The coverage on each feature (CDS, UTR) was normalized to the length of this feature.
**e** Genome-wide read density profiles around annotated start (top) and stop codons (bottom) for each replicate of OPCs and oligodendrocytes footprints. nt: nucleotide.
**f** Percentage of reads in each reading frame from the ribosome footprint samples of each replicate of OPCs and oligodendrocytes (ribosomeProfilingQC Bioconductor package).

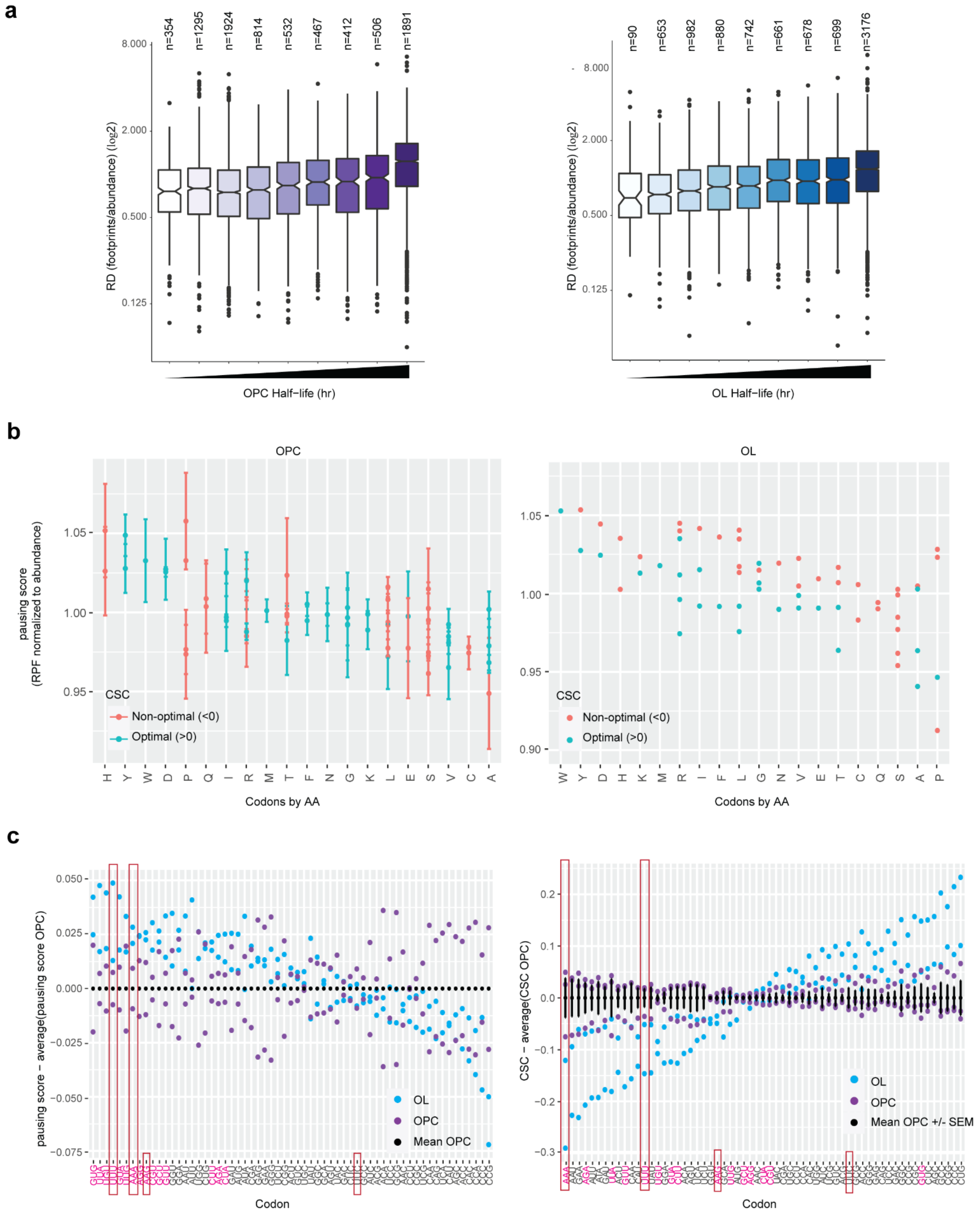

a
n=354
n=1295
n=1924
n=814
n=532
n=467
n=412
n=506
n=1891
8.000
2.000
0.500
0.125
RD (footprints/abundance) (log2)
OPC Half−life (hr)
n=90
n=653
n=982
n=880
n=742
n=661
n=678
n=699
n=3176
8.000
2.000
0.500
0.125
RD (footprints/abundance) (log2)
OL Half−life (hr)
b
OPC
pausing score
(RPF normalized to abundance)
1.05
1.00
0.95
CSC
Non-optimal (<0)
Optimal (>0)
H Y W D P Q I R M T F N G K L E S V C A
Codons by AA
OL
1.05
1.00
0.95
0.90
CSC
Non-optimal (<0)
Optimal (>0)
W Y D H K M R I F L G N V E T C Q S A P
Codons by AA
c
0.050
0.025
0.000
−0.025
−0.050
−0.075
pausing score − average(pausing score OPC)
OL
OPC
Mean OPC
Codon
0.2
0.1
0.0
−0.1
−0.2
−0.3
CSC − average(CSC OPC)
OL
OPC
Mean OPC +/- SEM
Codon

**Supplementary Fig. 8 - Related to Fig. 5. Ribosome density and mRNA stability in oligodendrocytes**

**a** Boxplot of ribosome density, RD (footprints FPKM normalized to total RNA seq FPKM) binned by increasing half-life, in OPCs (left) and oligodendrocytes (OL) (right). Number of transcripts in bin is indicated above each boxplot. Boxplots display the median with hinges at the 25$^{th}$ and 75$^{th}$ percentiles and whiskers extending 1.5 times the interquartile range. Both in OPCs and oligodendrocytes, $p < 2.2 \times 10^{-16}$, Kruskal-Wallis test.

**b** Ribosome pause scores for each codon in OPCs and oligodendrocytes, represented by amino acid (x-axis). The codons with optimal CSC value are represented by tile dots, non-optimal codons by pink dots. Represented are the means of two biological replicates +/- SEM.

**c** Pausing score and CSC differences between OPCs and oligodendrocytes, represented as the difference between each value and the average value in OPCs. N= 2 independent experiments for OPCs and oligodendrocytes for the pause scores, and n= 3 biological replicates for OPCs and 2 for oligodendrocytes for the CSC values. In black are the mean values for OPCs pausing scores and mean +/- SEM for OPCs CSC values. The codons in pink are the ones for which the differences in pause score from oligodendrocytes to OPCs have the highest positive values. Phenylalanine and lysine codons are highlighted by red rectangles. Oligodendrocytes values are in blue, OPCs in purple.

**Supplementary Table 1. List of oligonucleotides, gene blocks, plasmids and yeast strains used in this paper.**

| **Oligonucleotides and gene blocks** | | | |
|---|---|---|---|
| **Sequence** | **Description** | **Name** | **Source** |
| 5'-TGTGCTGTTCTCACACAGAGG-3' | *Cspg4* PCR Forward | oJC3322 | This paper |
| 5'-AGGTGGTGAGGACAGTAGGAG-3' | *Cspg4* PCR Reverse | oJC3323 | This paper |
| 5'-TGGCACTGTTCTGTGGATGT-3' | *Plp1/DM20* PCR Forward | oJC3330 | This paper |
| 5'-GACAGAAGGTTGGAGCCACA-3' | *Plp1/DM20* PCR Reverse | oJC3331 | This paper |
| 5'-GGGCCAAAGCCTCAAGGA-3' | *Gfap* PCR Forward | oJC4498 | This paper |
| 5'-AGAAAGTCTGTACAGGAATGGTGATG-3' | *Gfap* PCR Reverse | oJC4499 | This paper |
| 5'-TTCCAACTGGATACTGrGrA-3' | QuantM-seq 5' adapter | oJC4190 | Pinkard, 2020 |
| 5'-TTCCAACTGGATACTGrGrU-3' | QuantM-seq 5' adapter | oJC4191 | Pinkard, 2020 |
| 5'-TTCCAACTGGATACTGrGrC-3' | QuantM-seq 5' adapter | oJC4192 | Pinkard, 2020 |
| 5'-TTCCAACTGGATACTGrGrG-3' | QuantM-seq 5' adapter | oJC4193 | Pinkard, 2020 |
| /5'Phos/GTATCCAGTT-GGAATTCTCGGGTGCCAAGG/3ddC/ | QuantM-seq 3' adapter | oJC3385 | Pinkard, 2020 |
| /5'Phos/NNGATCGTCGGACTG-TAGAACTCTGAACGTGT/iSp18/CCTTGG-CACCCGAGAATTCCAACTGGATAC-3' | QuantM-seq RT primer | oJC4291 | Pinkard, 2020 |
| 5'-AATGATACGGCGACCACCGA-GATCTACACGTTCAGAGTTCTACAG-TCCGA-3' | QuantM-seq PCR primer | oJC4033 | Pinkard, 2020 |
| 5'-CAAGCAGAAGACGGCATACGA-GAT[BARCODE]GTGACTGGAGTTCCTT-GGCACCCGAGAATTCCA-3' | QuantM-seq PCR bar-coded primer (Illumina compatible barcodes) | | Pinkard, 2020 |
| 5'-CCTTTAGATCTTCAGTCTAACGC-3' | Northern probe Phe-GAA ACL | oJC4189 | This paper |
| 5'-CGCCCGAACAGGGACTTGAACCCTG-GACCCTCAGATTAAAAGTCTGATGCTC-TACCGACTGAGCTATCCGGGC-3' | Northern probe Lys-UUU FL | oJC4370 | This paper |
| 5'-TGCGTGTCATCCTTGCGCAG-3' | Northern probe U6 | oJC4339 | This paper |
| 5'-TGCCGAAACCCGGGATCGAAC-CAGGGACCTTTAGATCTTCAGTCTAAC-GCTCTCCCAACTGAGCTATTTCGGC-3' | Northern probe Phe-GAA FL | oJC4378 | This paper |
| 5'-GGAGACTGCGACCTGAACGCAG-3' | Northern probe Leu-CAG ACL | oJC4188 | This paper |

| | | | |
|---|---|---|---|
| 5’-CTCCCAACTGAGCTATTTCGG-3’ | Northern probe Phe-GAA D-loop | oJC4668 | This paper |
| 5’-AAACCCGGGATCGAACCAGG-3’ | Northern probe Phe-GAA TψC-loop | oJC4667 | This paper |
| 5’-CTACCGACTGAGCTATCCGG-3’ | Northern probe Lys-UUU D-loop | oJC4673 | This paper |
| 5’-GAACAGGGACTTGAACCCTG-3’ | Northern probe Lys-UUU TψC-loop | oJC4672 | This paper |
| 5’-CATGCAAAGCATGCGCTCTAC-3’ | Northern probe Ala ACL | oJC4335 | This paper |
| 5’-TTCGGTTAACAGCCGAACGC-3’ | Northern probe Asn-GUU ACL | oJC4277 | This paper |
| 5’-CGCGTGACAGGCGGGGATA-3’ | Northern probe Asp-GUC ACL | oJC4274 | This paper |
| 5’-CCTCTTGATCTGCAGTCAAA-3’ | Northern probe Cys-GCA ACL | oJC4119 | This paper |
| 5’-TGGCGTTATTAGCACCACGC-3’ | Northern probe Ile-AAU ACL | oJC4336 | This paper |
| 5’-TACCAGGAGTGGGGTTCGAACCCAC-GCGGACATATGTCCATT-GGATCTTAAGTCCAAC-GCCTTAACCACTCGGC-3’ | Northern probe Leu-UAA FL | oJC4384 | This paper |
| 5’-AGGCTCTACTGGGATTCGAACCCAG-GATCTCCTGTTTACAAGA-CAGGCGCTTTAAC-CAACTAAGCCATAGAGCC-3’ | Northern probe Thr-UGU FL | oJC4382 | This paper |
| 5’-CCTGAGATTAAGAGTCTCATGC-3’ | Northern probe Lys-CUU ACL | oJC4331 | This paper |
| 5’-ACCTTTCGCGTGTGAGGCGA-3’ | Northern probe Val-CAC/AAC ACL | oJC4332 | This paper |
| 5’-TGCTGCGGCCACAACGCAGAGTA-3’ | Northern probe His-GUG ACL | oJC4224 | This paper |
| 5’-CAATGGATTAGCAGTCCATCG-3’ | Northern probe Ser-GCU ACL | oJC4273 | This paper |
| 5’-CTACCACTGAGCTACATCCC-3’ | Northern probe Ala D-loop | oJC4674 | This paper |
| 5’-CCGCGTGGCAGGCGAGAAT-3’ | Northern probe Gly-GCC ACL | oJC4122 | This paper |
| 5’-CTCTGGGTTATGGGCCCAGCACGC-3’ | Northern probe iMet ACL | oJC3761 | This paper |
| 5’-TGTCAGAAGTGGGATTCGAACCCAC-GCCTCCATTGGAGACCAGAACTTGAG-TCTGGCGCCTTAGACCACTCGGC-3’ | Northern probe Leu-CAA FL | oJC4386 | This paper |
| 5’-AGGCCTCGCTGGGATTCGAACCCAG-GATCTCCTGTTTACTAGA-CAGGCGCTTTAAC-CAGCTAAGCCACGAAGCC-3’ | Northern probe Thr-AGU FL | oJC4385 | This paper |
| 5’-CGCCTGGGTGAAAACCAGGA-3’ | Northern probe Glu-UUC ACL | oJC4187 | This paper |
| 5’-TAGTCGGCAGGATTCGAACCTG-3’ | Northern probe Ser-AGA/UGA TψC-loop | oJC4222 | This paper |

| | | | |
|---|---|---|---|
| 5’-GGCGGTGAGAGCGCCGAAT-3’ | Northern probe Glu-CUC ACL | oJC4186 | This paper |
| 5’-TCCCCGTCGGGGAATCGAAC-3’ | Northern probe Asp-GUC TψC-loop | oJC4275 | This paper |
| 5’-ACGAGGATGGGATTCGAACCCA-3’ | Northern probe Ser-GCU TψC-loop | oJc4223 | This paper |
| 5’-ACCCCGACGTGATTTGAACAC-3’ | Northern probe Trp TψC-loop | oJC4327 | This paper |
| 5’-CTACAGTCCTCCGCTCTACC-3’ | Northern probe Tyr-GUA ACL | oJC4276 | This paper |
| 5’-AGCCAGCCAGGAGTCGAACCT-3’ | Northern probe Arg TψC-loop | oJC4329 | This paper |
| 5’-GCTGGATTCAGAGTCCAGAG-3’ | Northern probe Gln-CUG/UUG ACL | oJC4337 | This paper |
| 5’-CTTCAGATTATGAGACTGACGCGC-3’ | Northern probe Met ACL | oJC3762 | This paper |
| 5’-GTTTCCGCCCGGTTTCGAAC-3’ | Northern probe Val-CAC/AAC TψC-loop | oJC4333 | This paper |
| 5′-(Phos)-AGATCGGAAGAGCGTCGTG-TAGGGAAAGAGTG-TAGATCTCGGTGGTCGC-(SpC18)-CAC-TCA-(SpC18)-TTCAGAC-GTGTGCTCTTCCGATCTATTGATGGTGCC TACAG-3′ | Reverse transcription primer for decay-seq and ribo-seq libraries | oJC3453 | Ingolia, 2012 |
| 5′-AUGUACACGGAGUCGACCCAACGCGA-(Phos)-3′ | 26 nt RNA size marker for decay-seq and ribo-seq libraries | oKB687 | Ingolia, 2012 |
| 5′-AUGUACACGGAGUCGAGCUCAACCCG-CAACGCGA-(Phos)-3′ | 34 nt RNA size marker for decay-seq and ribo-seq libraries | oKB688 | Ingolia, 2012 |
| 5’-AUGUACACGGAGUCGAGCUCAACCCG-CAACGCGAACCGAUCCUAGAGGUCUAG-CAAGAUCGGCCAUGAAU-(Phos)-3′ | 70 nt RNA size marker for decay-seq libraries | oJC3131 | Forrest, 2020 |
| 5′-AATGATACGGCGACCACCGA-GATCTACAC-3′ | Forwarsd PCR primer for decay-seq and ribo-seq libraries | oJC4547 | Ingolia, 2012 |
| 5′-CAAGCAGAAGACGGCATACGAGAT-TGGTCA-GTGACTGGAGTTCAGAC-GTGTGCTCTTCCG-3’ | Reverse PCR primer for decay-seq and ribo-seq libraries | oKB690 | Forrest, 2020 |
| 5′-CAAGCAGAAGACGGCATACGAGAT-CACTGT-GTGACTGGAGTTCAGAC-GTGTGCTCTTCCG-3’ | Reverse PCR primer for decay-seq and ribo-seq libraries | oKB691 | Forrest, 2020 |
| 5′-CAAGCAGAAGACGGCATACGAGAT-ATTGGC-GTGACTGGAGTTCAGAC-GTGTGCTCTTCCG-3’ | Reverse PCR primer for decay-seq and ribo-seq libraries | oKB692 | Forrest, 2020 |
| 5′-CAAGCAGAAGACGGCATACGAGAT-TCAAGT-GTGACTGGAGTTCAGAC-GTGTGCTCTTCCG-3’ | Reverse PCR primer for decay-seq and ribo-seq libraries | oKB693 | Forrest, 2020 |
| 5′-CAAGCAGAAGACGGCATACGAGAT-CTGATC-GTGACTGGAGTTCAGAC-GTGTGCTCTTCCG-3’ | Reverse PCR primer for decay-seq and ribo-seq libraries | oKB694 | Forrest, 2020 |

| | | | |
|---|---|---|---|
| 5′-CAAGCAGAAGACGGCATACGAGAT-TACAAG-GTGACTGGAGTTCAGAC-GTGTGCTCTTCCG-3’ | Reverse PCR primer for decay-seq and ribo-seq libraries | oKB695 | Forrest, 2020 |
| 5′-CAAGCAGAAGACGGCATACGAGAT-CGTGAT-GTGACTGGAGTTCAGAC-GTGTGCTCTTCCG-3’ | Reverse PCR primer for decay-seq and ribo-seq libraries | oJC2796 | Forrest, 2020 |
| 5′-CAAGCAGAAGACGGCATACGAGAT-ACATCG-GTGACTGGAGTTCAGAC-GTGTGCTCTTCCG-3’ | Reverse PCR primer for decay-seq and ribo-seq libraries | oJC2797 | Forrest, 2020 |
| 5’-ccggaattcAGAGGGAATGACATT-GTTCTTACACGGCACAAGCAGA-CAAAATCAACATGGTCATTTAGAAATCGGAGGTGTGGATGCTCTCTATTTAGCGGA-GAAATATGGTACACCTCTTTAC-GTATATGATGTGGCTTTAATACGTGAGCGTGCTAAAAGCTTTAA-GCAGGCGTTTATTTCTGCAGGGCTGAAA-GCACAGGTGGCAT-ATGCGAGCAAAGCATTCTCATCAGTCGCAATGATTCAGCTCGCTGAGGAA-GAGGGACTTTCTTTAGATGTCGTATCCG-GAGAGAGCTATA-TACGGCTGTTGCAGCAGGCTTTCCGGCAGAACGCATCCACTTTCATGGAAACAATAA-GAGCAGGGAAGAACTGCG-GATGGCGCTTGAG-CACCGCATCGGCTGCATTGTGGTGGATAATTTCTATGAAATCGTCGCTTCTTGAA-GACCTATGTAAAGAAACGGGTCAC-TCCATCGATGTTCTTCTTCGGATactagtctag-3’ | Spike-in for decay-seq (gBlock, portion of LYSa gene from B. subtilis (nt 355-834) cloned into pBluescript SK(+)) | oJC3075 | Forrest, 2020 |

| | | | |
|---|---|---|---|
| 5’-ccggaattcACGGTGGAGGTCTATATAA-GCAGAGCTCTCTGGCTAACTAGA-GAACCCAC-TGCTTACTGGCTTATCGAAATTAATACGACTCACTATAGGGAGACCCAAGCTTGG-CATTCCGGTACTGTTGGTAAAGCCAC-CATGGAAGAC-GCCAAAAACATAAAGAAAGGCCCGGCGCCATTCTATCCGCTGGAAGATGGAAC-CGCTGGAGAGCAACTGCATAAGGC-TATGAAGAGATAC-GCCCTGGTTCCTGGAACAATTGCTTTTACAGATGCACATATCGAGGTGGACATCAC-TTACGCTGAG-TACTTCGAAATGTCCGTTCGGTTGG-CAGAAGCTATGAAACGATATGGGCTGAATACAAATCACAGAATCGTCGTATGCAG-TGAAAACTCTCTTCAATTCTTTATGCCGGTGTTGGGCGCGTTATTTATCGGAGTT-GCAGTTGCGCCCGCGAAC-GACATTTATAATGAAC-GTGAATTGCTCAACAGTATGGGCATTTCGCAGCCTAC-CGTGGTGTTCGTTTCCAAAAAGGGGTT-GCAAAAAATTTTGAACGTGCAAAAAAA-GCTCCCAATCATCCAAAAAATTATTATCATGGATTCTAAAACGGATTACCAGGGAT-TTCAGTCGATGTACAC-GTTCGTCACATCTCATCTAC-CTCCCGGTTTTAATGAATACGATTTTGTGCCAGAGTCCTTCGATAGGGACAAGA-CAATTGCACTGATCATGAACTCCTCTG-GATCTACTGGTCTGCCTAAAGGTGTCGCTCTGCCTCATAGAACTGCCTGCGTGAGAT-TCTCGCATGCCAGAGATCCTATTTTTGG-CAATCAAATCATTCCGGATACTGCGATTTTAAGTGTTGTTCCATTCCATCACGGTTTT-GGAATGTTTACTACACTCGGATATTTGA-TATGTGGATTTCGAGTCGTCTTAATGTATAGATTTGAAGAAGAGCTGTTTCTGAG-GAGCCTTCAGGATTACAAGATTCactag-tctag-3’ | Spike-in for decay-seq (gBlock, portion of firefly luciferase (nt 18 to 997) cloned into pBluescript SK(+)) | oJC3076 | Forrest, 2020 |
| 5’-gctggctaggtaagcttgatatcgccaccAtggtgag-caagggcgaggagctgttcac-cggggtggtgcccatcctggtcgagctggacggcgacgtaaac-ggccacaagttcagcgtgtccggcgagggcgagggcgatgccacctacggcaagctgaccctgaagttcatctgcac-caccggcaa-gctgcccgtgccctggcccaccctcgtgaccaccctgacctacggcgtgcagtgcttcagccgctaccccgaccacatgaa-gcagcac-gacttcttcaagtccgccatgcccgaaggctacgtccagga | gBlock with optimal eGFP to insert into EF.CMV.RFP | oJC4794 | This paper |

| | | | |
|---|---|---|---|
| gcgcaccatcttcttcaaggacgacggcaactacaa-<br>gacccgcgccgaggtgaagttcgagggcgacaccctggtg<br>aaccgcatcgagctgaagggcatcgacttcaaggaggac-<br>ggcaacatcctggggcacaa-<br>gctggagtacaactacaacagccacaacgtctatatcatgg<br>ccgacaagcagaagaacggcatcaaggtgaacttcaa-<br>gatccgccacaacatcgaggacgg-<br>cagcgtgcagctcgccgaccactaccagcagaacacccc<br>catcggcgacggccccgtgctgctgcccgacaaccactac-<br>ctgagcacccag-<br>tccgccctgagcaaagaccccaacgagaagcgcgatcac<br>atggtcctgctggagttcgtgaccgccgccgggatcac-<br>tctcggcatggacgagctg-<br>tacaaGtaaagcggccgcactcctcaggtgcaggctgcct<br>atcagaaggtggtggctggtgtggccaatgccctggctcac<br>aaataccactgagatctttttccctctgccaaaaattatggg-<br>gacatcatgaagccccctt-<br>gagcatctgacttctggctaataaaggaaatttattttcattgca<br>atagtgtgttggaattttttgtgtctctcactcggaaggacat-<br>atgggagatatcgaattcctgcagcccta-3’ | | | |
| 5’-gctggctaggtaagcttgatatcgccaccAtggtgag-<br>taaaggagaagaacttttcacag-<br>gagttgttccaatattagttgaacttgatggagatgtaaatggt<br>cacaaatttagcg-<br>tatctggtgaaggtgaaggtgatgcaacttatggaaaactta<br>cacttaaatttatatgtactacaggtaaacttccagtacctt-<br>ggcctacattagttacta-<br>cattaacgtatggagtacaatgcttttcaagatatccagatcat<br>atgaaacaacacgat-<br>ttctttaaatcagcgatgcctgaaggttatgtacaggaac-<br>gaacaa-<br>tattctttaaagatgatggaaattataaaactagggcggaagt<br>taaatttgaaggtgatacacttgtaaa-<br>taggattgaacttaaaggaattgattttaaagaa-<br>gatggaaatatattaggacacaaacttgaatataactataatt<br>ctcacaatgtatatattatggcaga-<br>taaacagaaaaatggaattaaagttaattttaaaa-<br>taaggcataatattgaagatggatcagttcaacttgcggatca<br>ttatcaacaaaatactcctataggtgatggaccag-<br>tattacttccagataatcactatttaa-<br>gcacacagagtgcgcttagcaaagatcctaatgaaaaaag<br>ggatcacatggtattacttgaatttgtaacagcggcaggaa-<br>taacattag-<br>gaatggatgaactttataaAtaaagcggccgcactcctcag<br>gtgcaggctgcc-<br>tatcagaaggtggtggctggtgtggccaatgccctggctcac<br>aaataccactgagatctttttccctctgccaaaaattatggg-<br>gacatcatgaagccccctt-<br>gagcatctgacttctggctaataaaggaaatttattttcattgca<br>atagtgtgttggaattttttgtgtctctcactcggaaggacat-<br>atgggagatatcgaattcctgcagcccta-3’ | gBlock with non-optimal GFP to insert into EF.CMV.RFP | oJC4795 | This paper |

| | | | |
|---|---|---|---|
| 5’-gctggctaggtaagcttgatatcgccaccAtggtgag-caaaggcgaggagctgtttac-cggggtggtgcccatcctggtcgagctggacggcgacgtaaac-ggccacaaatttagcgtgtccggcgagggcgagggcgatgccacctacggcaaactgaccctgaaatttatctgcaccac-cgg-caaactgcccgtgccctggcccaccctcgtgaccaccctgacctacggcgtgcagtgctttagccgctaccccgac-cacatgaaacagcac-gactttttaaatccgccatgcccgaaggctacgtccaggagcgcaccatctttttaaagacgacggcaac-tacaaaacccgcgccgaggtgaaattt-gagggcgacaccctggtgaaccgcatcgagctgaaaggcatcgactttaaagaggacggcaacatcctgggg-cacaaactggagtacaac-tacaacagccacaacgtctatatcatggccgacaaacagaaaaacgg-catcaaagtgaactttaaaatccgccacaacatcgaggac-ggcagcgtg-cagctcgccgaccactaccagcagaacacccccatcggcgacggccccgtgctgctgcccgacaaccactacctgag-cacccagtccgccctgagcaaa-gaccccaacgagaaacgcgatcacatggtcctgctggagtttgtgaccgccgccgggatcactctcggcatggacgagctg-tacaaAtaaagcggccgcac-tcctcaggtgcaggctgcctatcagaaggtggtggctggtgtggccaatgccctggctcacaaataccactga-gatctttttccctctgccaaaaattatggg-gacatcatgaagccccttgagcatctgacttctggctaataaaggaaatttattttcattgcaatagtgtgttggaattttt-gtgtctctcactcg-gaaggacatatgggagatatcgaattcctgcagcccta-3’ | gBlock with eGFP in which Phe and Lys are substituted to non-optimal codons, to insert into EF.CMV.RFP | oJC4803 | This paper |
| 5’-TTGATCAACAATAGGGAATAACTG-TAACAGTAAGTAAATTCG-GATCCCCGGGTTAATTAA-3’ | Forward primer to delete *TYW1* using Longtine vectors | oJC5217 | This paper |
| 5’-ATATATACGTGA-TATTACTCTTAATATATACCAGTATCTT-GAATTCGAGCTCGTTTAAAC-3’ | Reverse primer to delete *TYW1* using Longtine vectors | oJC5218 | This paper |
| 5’-TGCGAATTCTGTGGATCGAACACAG-GaCCTCCAGATCTTCAG-TCTGGCGCTCTCCCAACTGAGCTAAATCCGC-3’ | Northern probe Phe-GAA FL for yeast | oJC5126 | This paper |
| 5’-TGGTTGCTAAGAGATTCGAACTCTT-GCATCTTACGATACCTGAGCTT-GAATCAGGCGCCTTa-gACCGCTCGGCCAAACAACC-3’ | Northern probe Leu-CAA FL for yeast | oJC5129 | This paper |
| 5’-TGCGTGTCATCCTTGCGCAG-3’ | Northern probe U6 for yeast | oJC4339 | This paper |
| 5’-TTCCCACACCGGGAG-TCGAACCCGGGCCGCCTGGGTGAAAAC-CAGGAATCCTaACCGCTA-GACCATGTGGGA-3’ | Northern probe Glu-UUC FL (mouse) | oJC4376 | This paper |

| | | | |
|---|---|---|---|
| 5'- AGGTCCCACCGAGATTTGAACTCG-GATCGCTGGATTCAAAGTCCAGAG-TGCTaACCATTACACCATGGGGCC-3' | Northern probe Gln-UUG FL (mouse) | oJC5573 | This paper |
| 5'-GCTTGGTACTAATACGACTCACTA-3' | qPCR primer eGFP Forward | oJC4812 | This paper |
| 5'-CACCATGGTGGCGATATCAAG-3' | qPCR primer eGFP Reverse | oJC4813 | This paper |
| 5'-CCACTACCTGGTGGAGTTCAAGT-3' | qPCR primer RFP Forward | oJC4810 | This paper |
| 5'-GCTTGGCGTCCACGTAGTAGTA-3' | qPCR primer RFP Reverse | oJC4811 | This paper |
| | | | |
| **Yeast strains** | | | |
| **Genotype** | **Name** | **Source** | |
| MATa, *ura3, leu2, his3, met15* | yJC151 | This paper | |
| MATa, *ura3, leu2, his3, met15, TYW1::HIS5* | yJC3063 | This paper | |
| | | | |
| **Plasmids** | | | |
| **Description** | **Name** | **Source** | |
| EF.CMV.RFP | EF.CMV.RFP | Addgene plasmid # 17619 | |
| EF.eGFP.CMV.RFP, EF.CMV.RFP with insertion of eGFP under EF1a promoter | pJC1191 | This paper | |
| EF.eGFP-KF.CMV.RFP, EF.eGFP.CMV.RFP with substitutions of AAG and TTC for AAA and TTT in the GFP sequence | pJC1196 | This paper | |
| EF.nonoptGFP.CMV.RFP, EF.eGFP.CMV.RFP with substitutions of all optimal to non-optimal codons in the GFP sequence | pJC1192 | This paper | |
