# Supplementary material for: Oligodendrocyte differentiation alters tRNA modifications and codon optimality-mediated mRNA decay
Source: Nat Commun. 2022 Aug 25;13:5003. doi: 10.1038/s41467-022-32766-3 (PMC9411196; doi:10.1038/s41467-022-32766-3)
Supplement: Supplementary file 2 — Description of Additional Supplementary Files [file 41467_2022_32766_MOESM2_ESM.pdf]

Description of Additional Supplementary Files

File Name: Supplementary Software 1
Description: Code files describing QuantM-seq, Decay-seq and ribosome profiling processing and plotting
