# Supplementary material for: Oligodendrocyte differentiation alters tRNA modifications and codon optimality-mediated mRNA decay
Source: Nat Commun. 2022 Aug 25;13:5003. doi: 10.1038/s41467-022-32766-3 (PMC9411196; doi:10.1038/s41467-022-32766-3)
Supplement: Supplementary file 3 — Supplementary Software 1 [file 41467_2022_32766_MOESM3_ESM.zip › Code/tRNA_core_yeast.nb.html]

tRNA-seq Analysis, 10/31/18, TJ Sweet


Code 

- Show All Code
- Hide All Code
- Download Rmd

# tRNA-seq Analysis, 10/31/18, TJ Sweet

#SETUP - MUST DO BEFORE RUNNING R CODE:

1:Genomics core provides FastQC reports, de-multiplexes, and removes
PhiX reads. Results in a .fastq file for each library. If not already
done, gzip these fastq files with gzip in linux.

2:Move .fastq.gz files, bowtie2 index, and
“tRNA\_seq\_processing\_script.txt” to the working directory.

3:Run “tRNA\_seq\_processing\_script.txt” in bash. Make sure to set
number of processors for bowtie2 to use (number of processors on system
- 1). This script iterates over every .fastq file and performs each of
these operations in order: A:Trim adapters and CCA with cutadapt B:Map
reads to hg38 tRNAs with bowtie2 in very-sensitive mode C:Capture
statistics for each step in “processing.log” and “mapping.log”
D:Converts .sam to .bam and removes .sam to save space.

4:Manually set working directory and sampleTable in “R SCRIPT SETUP”
code chunk below

#R SCRIPT SETUP


```
setwd("/Users/thomassweet/Desktop/Coller Lab/Projects/tRNAseq/031919_NextSeq_HeLa_yeast/Yeast/")

#Call in packages needed
library(data.table)
library(tidyr)
library(Rsubread)
library(ggplot2)
library(Biostrings)
library(stringr)
library(reshape2)
library(gplots)
library(plyr)
library(corrplot)
library(DESeq2)
library(dplyr)

#Call in list of data files
#bamFiles <- list.files(pattern="\\.bam$",full.names = F)
bamFiles <- dir(".", "bam$")
sampleTable <- data.frame(bamFiles=bamFiles,sample=c("Yeast_1_YAMAT","Yeast_2_YAMAT","Yeast_1_circ","Yeast_2_circ"))

sampleTable

#Force number printout instead of scientific notation
options("scipen"=100, "digits"=4)
```


#COUNT READS OVER tRNA GENES


```
#Make annotation files for counting. "tRNA_names_size.txt" is created by the script above from the bowtie2 index mapped to.
#Need columns "GeneID","Chr","Start","End","Strand"
tRNA.annot <- as.data.frame(fread("tRNA_names_sizes.txt"))
tRNA.annot <- data.frame(GeneID=sub(" .*","",tRNA.annot$V2),Chr=sub(" .*","",tRNA.annot$V2),Start=1,End=tRNA.annot$V3,Strand="+")                            
#Count reads over tRNA genes
tRNA.counts <- featureCounts(bamFiles,annot.ext = tRNA.annot,strandSpecific = 1,useMetaFeatures=F,minMQS=10)
```


#ALL TRNA GENES #CONVERT TO READS PER MILLION #MAKE SURE TO MANUALLY
CONVERT THE PATHS ON LINES 75 AND 76 TO WHATEVER YOUR FOLDER IS #OUTPUTS
GENE-LEVEL CSV FILE


```
#Call in reads and total library stats from tRNA.counts object above
tRNA.RPM <- as.data.frame(tRNA.counts$counts)
tRNA.stat <- as.data.frame(tRNA.counts$stat)
rownames(tRNA.stat) <- tRNA.stat[,1]
tRNA.stat <- tRNA.stat[,2:ncol(tRNA.stat)]

tRNA.RPM

#Change column names to simplify (featureCounts takes the whole path of bam file) 
colnames(tRNA.RPM) <- as.character(sampleTable$sample[match(as.character(sampleTable$bamFiles),gsub("X.Users.thomassweet.Desktop.Coller.Lab.Projects.tRNAseq.031919_NextSeq_HeLa_yeast.Yeast.","",colnames(tRNA.RPM)))])
colnames(tRNA.stat) <- as.character(sampleTable$sample[match(as.character(sampleTable$bamFiles),gsub("X.Users.thomassweet.Desktop.Coller.Lab.Projects.tRNAseq.031919_NextSeq_HeLa_yeast.Yeast.","",colnames(tRNA.stat)))])

#Convert to reads per million mapped reads or throw error if data don't line up
if(identical(colnames(tRNA.RPM),colnames(tRNA.stat))){

for(i in 1:ncol(tRNA.RPM)){
  tRNA.RPM[,i] <- (tRNA.RPM[,i] * 1000000) / (sum(tRNA.stat[1,i],tRNA.stat[3,i]))
}

} else {
  stop("tRNA.RPM and tRNA.stat dataframe columns are not in same order")
}

tRNA.RPM

tRNA.RPM.mito <- tRNA.RPM[(grep("tdb",rownames(tRNA.RPM))),]
tRNA.RPM.cyto <- tRNA.RPM[(grep("tdb",rownames(tRNA.RPM),invert=T)),]

tRNA.RPM.mito
tRNA.RPM.cyto

write.csv(tRNA.RPM.cyto,"Yeast_tRNA_gene_summary.csv")
```


#GENERATE PLOTS FOR ALL GENES #A LOT OF GENERIC PLOTS, NONE ARE SAVED
AS OUTPUT SO CHANGE CODE IF YOU WANT OUTPUT


```
#Correlation matrix between samples
#pdf("FILE_NAME")
#corrplot(cor(tRNA.RPM.mito), order = "hclust", tl.col = "black", tl.srt = 45)
#corrplot(cor(tRNA.RPM.cyto), order = "hclust", tl.col = "black", tl.srt = 45)
#dev.off

#Raw heatmaps over all genes
#pdf("FILE_NAME")
heatmap.2(sqrt(as.matrix(tRNA.RPM.cyto)),col="bluered",scale="none", trace="none",dendrogram="none", margin=c(7, 5),cexCol=0.9,cexRow=0.5,density.info="density",breaks = seq(0, 150, length.out = 20))
#dev.off

ggplot(data = melt(sqrt(tRNA.RPM.cyto)), aes(x=variable,y=value)) + geom_boxplot(aes(fill=variable)) + ylim(0,100)
ggplot(data = melt(sqrt(tRNA.RPM.cyto)), aes(x=value)) + geom_density(aes(colour=variable)) 
ggplot(data = melt(sqrt(tRNA.RPM.cyto)), aes(x=value)) + geom_density(aes(colour=variable)) + xlim(0,150)

heatmap.2(as.matrix(tRNA.RPM.cyto),col="bluered",scale="none", trace="none",dendrogram="none", margin=c(7, 5),cexCol=0.9,cexRow=0.5,density.info="density",breaks = seq(0, 10000, length.out = 20))
#dev.off

ggplot(data = melt(tRNA.RPM.cyto), aes(x=variable,y=value)) + geom_boxplot(aes(fill=variable)) + ylim(0,100)
ggplot(data = melt(tRNA.RPM.cyto), aes(x=value)) + geom_density(aes(colour=variable)) 
ggplot(data = melt(tRNA.RPM.cyto), aes(x=value)) + geom_density(aes(colour=variable)) + xlim(0,150)
```


#COLLAPSE ALL ISODECODERS INTO SINGLE ANTICODON COUNT


```
#get anticodons with string operations
anticodons <- gsub("^.*?-.*","",rownames(tRNA.RPM.cyto))
anticodons <- gsub("^([^-]*-[^-]*)-.*$","\\1",anticodons)
anticodon.counts <- as.data.frame(tRNA.RPM.cyto)
anticodon.counts$tRNA <- anticodons
anticodon.counts

#sum all 
anticodon.counts <- as.data.frame(setDT(anticodon.counts)[,lapply(.SD,sum),by=tRNA])
rownames(anticodon.counts) <- anticodon.counts$tRNA
anticodon.counts$tRNA <- NULL
anticodon.counts
```


#ANTICODON OPERATIONS #OUTPUTS ANTICODON-LEVEL CSV FILE


```
#Call in from previous code chunk
tRNA.RPM <- as.data.frame(anticodon.counts)

tRNA.RPM.mito <- tRNA.RPM[(grep("tdb",rownames(tRNA.RPM))),]

#Throw out mito tRNAs, ambiguous "Und-NNN" gene
tRNA.RPM.cyto <- tRNA.RPM[(grep("tdb",rownames(tRNA.RPM),invert=T)),]
tRNA.RPM.cyto <- tRNA.RPM[(grep("Und-NNN",rownames(tRNA.RPM),invert=T)),]

as.data.frame(tRNA.RPM.cyto)

write.csv(tRNA.RPM.cyto,"Yeast_tRNA_anticodon_summary.csv")
```


LS0tCnRpdGxlOiAidFJOQS1zZXEgQW5hbHlzaXMsIDEwLzMxLzE4LCBUSiBTd2VldCIKb3V0cHV0OiBodG1sX25vdGVib29rCi0tLQoKI1NFVFVQIC0gTVVTVCBETyBCRUZPUkUgUlVOTklORyBSIENPREU6IAoKMTpHZW5vbWljcyBjb3JlIHByb3ZpZGVzIEZhc3RRQyByZXBvcnRzLCBkZS1tdWx0aXBsZXhlcywgYW5kIHJlbW92ZXMgUGhpWCByZWFkcy4gUmVzdWx0cyBpbiBhIC5mYXN0cSBmaWxlIGZvciBlYWNoIGxpYnJhcnkuIElmIG5vdCBhbHJlYWR5IGRvbmUsIGd6aXAgdGhlc2UgZmFzdHEgZmlsZXMgd2l0aCBnemlwIGluIGxpbnV4LgoKMjpNb3ZlIC5mYXN0cS5neiBmaWxlcywgYm93dGllMiBpbmRleCwgYW5kICJ0Uk5BX3NlcV9wcm9jZXNzaW5nX3NjcmlwdC50eHQiIHRvIHRoZSB3b3JraW5nIGRpcmVjdG9yeS4gCgozOlJ1biAidFJOQV9zZXFfcHJvY2Vzc2luZ19zY3JpcHQudHh0IiBpbiBiYXNoLiBNYWtlIHN1cmUgdG8gc2V0IG51bWJlciBvZiBwcm9jZXNzb3JzIGZvciBib3d0aWUyIHRvIHVzZSAobnVtYmVyIG9mIHByb2Nlc3NvcnMgb24gc3lzdGVtIC0gMSkuIFRoaXMgc2NyaXB0IGl0ZXJhdGVzIG92ZXIgZXZlcnkgLmZhc3RxIGZpbGUgYW5kIHBlcmZvcm1zIGVhY2ggb2YgdGhlc2Ugb3BlcmF0aW9ucyBpbiBvcmRlcjoKICAgQTpUcmltIGFkYXB0ZXJzIGFuZCBDQ0Egd2l0aCBjdXRhZGFwdAogICBCOk1hcCByZWFkcyB0byBoZzM4IHRSTkFzIHdpdGggYm93dGllMiBpbiB2ZXJ5LXNlbnNpdGl2ZSBtb2RlCiAgIEM6Q2FwdHVyZSBzdGF0aXN0aWNzIGZvciBlYWNoIHN0ZXAgaW4gInByb2Nlc3NpbmcubG9nIiBhbmQgIm1hcHBpbmcubG9nIgogICBEOkNvbnZlcnRzIC5zYW0gdG8gLmJhbSBhbmQgcmVtb3ZlcyAuc2FtIHRvIHNhdmUgc3BhY2UuCiAgIAo0Ok1hbnVhbGx5IHNldCB3b3JraW5nIGRpcmVjdG9yeSBhbmQgc2FtcGxlVGFibGUgaW4gIlIgU0NSSVBUIFNFVFVQIiBjb2RlIGNodW5rIGJlbG93CgojUiBTQ1JJUFQgU0VUVVAKCmBgYHtyIHdhcm5pbmc9RkFMU0V9CnNldHdkKCIvVXNlcnMvdGhvbWFzc3dlZXQvRGVza3RvcC9Db2xsZXIgTGFiL1Byb2plY3RzL3RSTkFzZXEvMDMxOTE5X05leHRTZXFfSGVMYV95ZWFzdC9ZZWFzdC8iKQoKI0NhbGwgaW4gcGFja2FnZXMgbmVlZGVkCmxpYnJhcnkoZGF0YS50YWJsZSkKbGlicmFyeSh0aWR5cikKbGlicmFyeShSc3VicmVhZCkKbGlicmFyeShnZ3Bsb3QyKQpsaWJyYXJ5KEJpb3N0cmluZ3MpCmxpYnJhcnkoc3RyaW5ncikKbGlicmFyeShyZXNoYXBlMikKbGlicmFyeShncGxvdHMpCmxpYnJhcnkocGx5cikKbGlicmFyeShjb3JycGxvdCkKbGlicmFyeShERVNlcTIpCmxpYnJhcnkoZHBseXIpCgojQ2FsbCBpbiBsaXN0IG9mIGRhdGEgZmlsZXMKI2JhbUZpbGVzIDwtIGxpc3QuZmlsZXMocGF0dGVybj0iXFwuYmFtJCIsZnVsbC5uYW1lcyA9IEYpCmJhbUZpbGVzIDwtIGRpcigiLiIsICJiYW0kIikKc2FtcGxlVGFibGUgPC0gZGF0YS5mcmFtZShiYW1GaWxlcz1iYW1GaWxlcyxzYW1wbGU9YygiWWVhc3RfMV9ZQU1BVCIsIlllYXN0XzJfWUFNQVQiLCJZZWFzdF8xX2NpcmMiLCJZZWFzdF8yX2NpcmMiKSkKCnNhbXBsZVRhYmxlCgojRm9yY2UgbnVtYmVyIHByaW50b3V0IGluc3RlYWQgb2Ygc2NpZW50aWZpYyBub3RhdGlvbgpvcHRpb25zKCJzY2lwZW4iPTEwMCwgImRpZ2l0cyI9NCkKYGBgCgojQ09VTlQgUkVBRFMgT1ZFUiB0Uk5BIEdFTkVTCgpgYGB7cn0KI01ha2UgYW5ub3RhdGlvbiBmaWxlcyBmb3IgY291bnRpbmcuICJ0Uk5BX25hbWVzX3NpemUudHh0IiBpcyBjcmVhdGVkIGJ5IHRoZSBzY3JpcHQgYWJvdmUgZnJvbSB0aGUgYm93dGllMiBpbmRleCBtYXBwZWQgdG8uCiNOZWVkIGNvbHVtbnMgIkdlbmVJRCIsIkNociIsIlN0YXJ0IiwiRW5kIiwiU3RyYW5kIgp0Uk5BLmFubm90IDwtIGFzLmRhdGEuZnJhbWUoZnJlYWQoInRSTkFfbmFtZXNfc2l6ZXMudHh0IikpCnRSTkEuYW5ub3QgPC0gZGF0YS5mcmFtZShHZW5lSUQ9c3ViKCIgLioiLCIiLHRSTkEuYW5ub3QkVjIpLENocj1zdWIoIiAuKiIsIiIsdFJOQS5hbm5vdCRWMiksU3RhcnQ9MSxFbmQ9dFJOQS5hbm5vdCRWMyxTdHJhbmQ9IisiKSAgICAgICAgICAgICAgICAgICAgICAgICAgICAKI0NvdW50IHJlYWRzIG92ZXIgdFJOQSBnZW5lcwp0Uk5BLmNvdW50cyA8LSBmZWF0dXJlQ291bnRzKGJhbUZpbGVzLGFubm90LmV4dCA9IHRSTkEuYW5ub3Qsc3RyYW5kU3BlY2lmaWMgPSAxLHVzZU1ldGFGZWF0dXJlcz1GLG1pbk1RUz0xMCkKYGBgCgojQUxMIFRSTkEgR0VORVMKI0NPTlZFUlQgVE8gUkVBRFMgUEVSIE1JTExJT04KI01BS0UgU1VSRSBUTyBNQU5VQUxMWSBDT05WRVJUIFRIRSBQQVRIUyBPTiBMSU5FUyA3NSBBTkQgNzYgVE8gV0hBVEVWRVIgWU9VUiBGT0xERVIgSVMKI09VVFBVVFMgR0VORS1MRVZFTCBDU1YgRklMRQoKYGBge3J9CiNDYWxsIGluIHJlYWRzIGFuZCB0b3RhbCBsaWJyYXJ5IHN0YXRzIGZyb20gdFJOQS5jb3VudHMgb2JqZWN0IGFib3ZlCnRSTkEuUlBNIDwtIGFzLmRhdGEuZnJhbWUodFJOQS5jb3VudHMkY291bnRzKQp0Uk5BLnN0YXQgPC0gYXMuZGF0YS5mcmFtZSh0Uk5BLmNvdW50cyRzdGF0KQpyb3duYW1lcyh0Uk5BLnN0YXQpIDwtIHRSTkEuc3RhdFssMV0KdFJOQS5zdGF0IDwtIHRSTkEuc3RhdFssMjpuY29sKHRSTkEuc3RhdCldCgp0Uk5BLlJQTQoKI0NoYW5nZSBjb2x1bW4gbmFtZXMgdG8gc2ltcGxpZnkgKGZlYXR1cmVDb3VudHMgdGFrZXMgdGhlIHdob2xlIHBhdGggb2YgYmFtIGZpbGUpIApjb2xuYW1lcyh0Uk5BLlJQTSkgPC0gYXMuY2hhcmFjdGVyKHNhbXBsZVRhYmxlJHNhbXBsZVttYXRjaChhcy5jaGFyYWN0ZXIoc2FtcGxlVGFibGUkYmFtRmlsZXMpLGdzdWIoIlguVXNlcnMudGhvbWFzc3dlZXQuRGVza3RvcC5Db2xsZXIuTGFiLlByb2plY3RzLnRSTkFzZXEuMDMxOTE5X05leHRTZXFfSGVMYV95ZWFzdC5ZZWFzdC4iLCIiLGNvbG5hbWVzKHRSTkEuUlBNKSkpXSkKY29sbmFtZXModFJOQS5zdGF0KSA8LSBhcy5jaGFyYWN0ZXIoc2FtcGxlVGFibGUkc2FtcGxlW21hdGNoKGFzLmNoYXJhY3RlcihzYW1wbGVUYWJsZSRiYW1GaWxlcyksZ3N1YigiWC5Vc2Vycy50aG9tYXNzd2VldC5EZXNrdG9wLkNvbGxlci5MYWIuUHJvamVjdHMudFJOQXNlcS4wMzE5MTlfTmV4dFNlcV9IZUxhX3llYXN0LlllYXN0LiIsIiIsY29sbmFtZXModFJOQS5zdGF0KSkpXSkKCiNDb252ZXJ0IHRvIHJlYWRzIHBlciBtaWxsaW9uIG1hcHBlZCByZWFkcyBvciB0aHJvdyBlcnJvciBpZiBkYXRhIGRvbid0IGxpbmUgdXAKaWYoaWRlbnRpY2FsKGNvbG5hbWVzKHRSTkEuUlBNKSxjb2xuYW1lcyh0Uk5BLnN0YXQpKSl7Cgpmb3IoaSBpbiAxOm5jb2wodFJOQS5SUE0pKXsKICB0Uk5BLlJQTVssaV0gPC0gKHRSTkEuUlBNWyxpXSAqIDEwMDAwMDApIC8gKHN1bSh0Uk5BLnN0YXRbMSxpXSx0Uk5BLnN0YXRbMyxpXSkpCn0KCn0gZWxzZSB7CiAgc3RvcCgidFJOQS5SUE0gYW5kIHRSTkEuc3RhdCBkYXRhZnJhbWUgY29sdW1ucyBhcmUgbm90IGluIHNhbWUgb3JkZXIiKQp9Cgp0Uk5BLlJQTQoKdFJOQS5SUE0ubWl0byA8LSB0Uk5BLlJQTVsoZ3JlcCgidGRiIixyb3duYW1lcyh0Uk5BLlJQTSkpKSxdCnRSTkEuUlBNLmN5dG8gPC0gdFJOQS5SUE1bKGdyZXAoInRkYiIscm93bmFtZXModFJOQS5SUE0pLGludmVydD1UKSksXQoKdFJOQS5SUE0ubWl0bwp0Uk5BLlJQTS5jeXRvCgp3cml0ZS5jc3YodFJOQS5SUE0uY3l0bywiWWVhc3RfdFJOQV9nZW5lX3N1bW1hcnkuY3N2IikKCmBgYAojR0VORVJBVEUgUExPVFMgRk9SIEFMTCBHRU5FUwojQSBMT1QgT0YgR0VORVJJQyBQTE9UUywgTk9ORSBBUkUgU0FWRUQgQVMgT1VUUFVUIFNPIENIQU5HRSBDT0RFIElGIFlPVSBXQU5UIE9VVFBVVAoKYGBge3J9CiNDb3JyZWxhdGlvbiBtYXRyaXggYmV0d2VlbiBzYW1wbGVzCiNwZGYoIkZJTEVfTkFNRSIpCiNjb3JycGxvdChjb3IodFJOQS5SUE0ubWl0byksIG9yZGVyID0gImhjbHVzdCIsIHRsLmNvbCA9ICJibGFjayIsIHRsLnNydCA9IDQ1KQojY29ycnBsb3QoY29yKHRSTkEuUlBNLmN5dG8pLCBvcmRlciA9ICJoY2x1c3QiLCB0bC5jb2wgPSAiYmxhY2siLCB0bC5zcnQgPSA0NSkKI2Rldi5vZmYKCiNSYXcgaGVhdG1hcHMgb3ZlciBhbGwgZ2VuZXMKI3BkZigiRklMRV9OQU1FIikKaGVhdG1hcC4yKHNxcnQoYXMubWF0cml4KHRSTkEuUlBNLmN5dG8pKSxjb2w9ImJsdWVyZWQiLHNjYWxlPSJub25lIiwgdHJhY2U9Im5vbmUiLGRlbmRyb2dyYW09Im5vbmUiLCBtYXJnaW49Yyg3LCA1KSxjZXhDb2w9MC45LGNleFJvdz0wLjUsZGVuc2l0eS5pbmZvPSJkZW5zaXR5IixicmVha3MgPSBzZXEoMCwgMTUwLCBsZW5ndGgub3V0ID0gMjApKQojZGV2Lm9mZgoKZ2dwbG90KGRhdGEgPSBtZWx0KHNxcnQodFJOQS5SUE0uY3l0bykpLCBhZXMoeD12YXJpYWJsZSx5PXZhbHVlKSkgKyBnZW9tX2JveHBsb3QoYWVzKGZpbGw9dmFyaWFibGUpKSArIHlsaW0oMCwxMDApCmdncGxvdChkYXRhID0gbWVsdChzcXJ0KHRSTkEuUlBNLmN5dG8pKSwgYWVzKHg9dmFsdWUpKSArIGdlb21fZGVuc2l0eShhZXMoY29sb3VyPXZhcmlhYmxlKSkgCmdncGxvdChkYXRhID0gbWVsdChzcXJ0KHRSTkEuUlBNLmN5dG8pKSwgYWVzKHg9dmFsdWUpKSArIGdlb21fZGVuc2l0eShhZXMoY29sb3VyPXZhcmlhYmxlKSkgKyB4bGltKDAsMTUwKQoKaGVhdG1hcC4yKGFzLm1hdHJpeCh0Uk5BLlJQTS5jeXRvKSxjb2w9ImJsdWVyZWQiLHNjYWxlPSJub25lIiwgdHJhY2U9Im5vbmUiLGRlbmRyb2dyYW09Im5vbmUiLCBtYXJnaW49Yyg3LCA1KSxjZXhDb2w9MC45LGNleFJvdz0wLjUsZGVuc2l0eS5pbmZvPSJkZW5zaXR5IixicmVha3MgPSBzZXEoMCwgMTAwMDAsIGxlbmd0aC5vdXQgPSAyMCkpCiNkZXYub2ZmCgpnZ3Bsb3QoZGF0YSA9IG1lbHQodFJOQS5SUE0uY3l0byksIGFlcyh4PXZhcmlhYmxlLHk9dmFsdWUpKSArIGdlb21fYm94cGxvdChhZXMoZmlsbD12YXJpYWJsZSkpICsgeWxpbSgwLDEwMCkKZ2dwbG90KGRhdGEgPSBtZWx0KHRSTkEuUlBNLmN5dG8pLCBhZXMoeD12YWx1ZSkpICsgZ2VvbV9kZW5zaXR5KGFlcyhjb2xvdXI9dmFyaWFibGUpKSAKZ2dwbG90KGRhdGEgPSBtZWx0KHRSTkEuUlBNLmN5dG8pLCBhZXMoeD12YWx1ZSkpICsgZ2VvbV9kZW5zaXR5KGFlcyhjb2xvdXI9dmFyaWFibGUpKSArIHhsaW0oMCwxNTApCmBgYAoKCgojQ09MTEFQU0UgQUxMIElTT0RFQ09ERVJTIElOVE8gU0lOR0xFIEFOVElDT0RPTiBDT1VOVAoKYGBge3J9CiNnZXQgYW50aWNvZG9ucyB3aXRoIHN0cmluZyBvcGVyYXRpb25zCmFudGljb2RvbnMgPC0gZ3N1YigiXi4qPy0uKiIsIiIscm93bmFtZXModFJOQS5SUE0uY3l0bykpCmFudGljb2RvbnMgPC0gZ3N1YigiXihbXi1dKi1bXi1dKiktLiokIiwiXFwxIixhbnRpY29kb25zKQphbnRpY29kb24uY291bnRzIDwtIGFzLmRhdGEuZnJhbWUodFJOQS5SUE0uY3l0bykKYW50aWNvZG9uLmNvdW50cyR0Uk5BIDwtIGFudGljb2RvbnMKYW50aWNvZG9uLmNvdW50cwoKI3N1bSBhbGwgCmFudGljb2Rvbi5jb3VudHMgPC0gYXMuZGF0YS5mcmFtZShzZXREVChhbnRpY29kb24uY291bnRzKVssbGFwcGx5KC5TRCxzdW0pLGJ5PXRSTkFdKQpyb3duYW1lcyhhbnRpY29kb24uY291bnRzKSA8LSBhbnRpY29kb24uY291bnRzJHRSTkEKYW50aWNvZG9uLmNvdW50cyR0Uk5BIDwtIE5VTEwKYW50aWNvZG9uLmNvdW50cwpgYGAKCiNBTlRJQ09ET04gT1BFUkFUSU9OUwojT1VUUFVUUyBBTlRJQ09ET04tTEVWRUwgQ1NWIEZJTEUKCmBgYHtyfQoKI0NhbGwgaW4gZnJvbSBwcmV2aW91cyBjb2RlIGNodW5rCnRSTkEuUlBNIDwtIGFzLmRhdGEuZnJhbWUoYW50aWNvZG9uLmNvdW50cykKCnRSTkEuUlBNLm1pdG8gPC0gdFJOQS5SUE1bKGdyZXAoInRkYiIscm93bmFtZXModFJOQS5SUE0pKSksXQoKI1Rocm93IG91dCBtaXRvIHRSTkFzLCBhbWJpZ3VvdXMgIlVuZC1OTk4iIGdlbmUKdFJOQS5SUE0uY3l0byA8LSB0Uk5BLlJQTVsoZ3JlcCgidGRiIixyb3duYW1lcyh0Uk5BLlJQTSksaW52ZXJ0PVQpKSxdCnRSTkEuUlBNLmN5dG8gPC0gdFJOQS5SUE1bKGdyZXAoIlVuZC1OTk4iLHJvd25hbWVzKHRSTkEuUlBNKSxpbnZlcnQ9VCkpLF0KCmFzLmRhdGEuZnJhbWUodFJOQS5SUE0uY3l0bykKCndyaXRlLmNzdih0Uk5BLlJQTS5jeXRvLCJZZWFzdF90Uk5BX2FudGljb2Rvbl9zdW1tYXJ5LmNzdiIpCgoKYGBgCgoK
