# Supplementary material for: Oligodendrocyte differentiation alters tRNA modifications and codon optimality-mediated mRNA decay
Source: Nat Commun. 2022 Aug 25;13:5003. doi: 10.1038/s41467-022-32766-3 (PMC9411196; doi:10.1038/s41467-022-32766-3)
Supplement: Supplementary file 4 — Reporting Summary [file 41467_2022_32766_MOESM4_ESM.pdf]

nature portfolio

Corresponding author(s): Jeff Coller

Last updated by author(s): Aug 10, 2022

Reporting Summary

Nature Portfolio wishes to improve the reproducibility of the work that we publish. This form provides structure for consistency and transparency in reporting. For further information on Nature Portfolio policies, see our Editorial Policies and the Editorial Policy Checklist.

Statistics

For all statistical analyses, confirm that the following items are present in the figure legend, table legend, main text, or Methods section.

n/a	Confirmed	
☐	☒	The exact sample size (*n*) for each experimental group/condition, given as a discrete number and unit of measurement
☐	☒	A statement on whether measurements were taken from distinct samples or whether the same sample was measured repeatedly
☐	☒	The statistical test(s) used AND whether they are one- or two-sided<br>*Only common tests should be described solely by name; describe more complex techniques in the Methods section.*
☐	☒	A description of all covariates tested
☐	☒	A description of any assumptions or corrections, such as tests of normality and adjustment for multiple comparisons
☐	☒	A full description of the statistical parameters including central tendency (e.g. means) or other basic estimates (e.g. regression coefficient) AND variation (e.g. standard deviation) or associated estimates of uncertainty (e.g. confidence intervals)
☐	☒	For null hypothesis testing, the test statistic (e.g. *F*, *t*, *r*) with confidence intervals, effect sizes, degrees of freedom and *P* value noted<br>*Give P values as exact values whenever suitable.*
☒	☐	For Bayesian analysis, information on the choice of priors and Markov chain Monte Carlo settings
☒	☐	For hierarchical and complex designs, identification of the appropriate level for tests and full reporting of outcomes
☐	☒	Estimates of effect sizes (e.g. Cohen's *d*, Pearson's *r*), indicating how they were calculated

Our web collection on statistics for biologists contains articles on many of the points above.

Software and code

Policy information about availability of computer code

Data collection	Publicly available softwares: PerkinElmer Harmony and Columbus software, MassLynx v4.1 and TargetLynx (Waters), Gene Expression Omnibus (GEO), Panther, R v4.1.1 , RStudio v1.4.1717
Data analysis	Publicly available codes and softwares: cutadapt v2.8, bowtie2 v2.42, R v4.1.1 , R packages (Rsubread featureCounts, DESeq2, ggplot2, gplots, Rmisc, ballgown, Biostrings, dplyr, ggpubr, Bioconductor limma, Bioconductor GenomicAlignments and GenomicFeatures, Bioconductor ribosomeProfilingQC) and RStudio v1.4.1717 , ImageQuant, FastQC, fastx_clipper, hisat2 v2.1.0, stringtie v1.3.5, samtools v1.7-2, github.com/rasilab/adarnell_2018, and custom code for QuantM-seq is available in Pinkard et al, 2020. Analyses steps are also described in Supplementary Files.

For manuscripts utilizing custom algorithms or software that are central to the research but not yet described in published literature, software must be made available to editors and reviewers. We strongly encourage code deposition in a community repository (e.g. GitHub). See the Nature Portfolio guidelines for submitting code & software for further information.

Data

Policy information about availability of data

All manuscripts must include a data availability statement. This statement should provide the following information, where applicable:

- Accession codes, unique identifiers, or web links for publicly available datasets
- A description of any restrictions on data availability
- For clinical datasets or third party data, please ensure that the statement adheres to our policy

All datasets generated in this study (QuantM-seq, Decay-Seq, Ribosome profiling) have been deposited in Gene Expression Omnibus (https://www.ncbi.nlm.nih.gov/geo/) under the SuperSeries accession code GSE182811 (https://www.ncbi.nlm.nih.gov/geo/query/acc.cgi?acc=GSE182811). These include raw Fastq files as well as

processed data.
The expression dataset from myelinating oligodendrocytes, newly formed oligodendrocytes, whole cortex, OPCs and astrocytes are available in the GEO datatbase under accession code GSE52564 (https://www.ncbi.nlm.nih.gov/geo/query/acc.cgi?acc=GSE52564).
All uncropped and unprocessed Northern blots, Western blots and gels, as well as all the raw data points and mass spectrometry data, are provided in the Supplementary Information/Source Data file provided with this paper.
Other datasets are publicly available: GtRNAdb high confidence list of tRNAs for mouse (GRCm38) (http://gtrnadb.ucsc.edu/genomes/eukaryota/Mmusc10/Mmusc10-gene-list.html) and yeast (S288c) (http://gtrnadb.ucsc.edu/genomes/eukaryota/Scere3/Scere3-gene-list.html), hisat2 genome index (GRCm38 genome_tran, https://cloud.biohpc.swmed.edu/index.php/s/grcm38_tran/download), gff3 files for mouse canonical transcripts and coding sequence from Gencode vM9 (https://www.gencodegenes.org/mouse/release_M9.html), and mouse ribosomal RNA sequences from NCBI (https://www.ncbi.nlm.nih.gov/nuccore/).

Field-specific reporting

Please select the one below that is the best fit for your research. If you are not sure, read the appropriate sections before making your selection.

☒ Life sciences ☐ Behavioural & social sciences ☐ Ecological, evolutionary & environmental sciences

For a reference copy of the document with all sections, see nature.com/documents/nr-reporting-summary-flat.pdf

Life sciences study design

All studies must disclose on these points even when the disclosure is negative.

Sample size	We performed biological triplicates or duplicates for each analysis, which are accepted sample sizes in the field, with a high correlation between samples (See Statistical analysis in the Methods section).
Data exclusions	No data were excluded from the analyses.
Replication	All attempts at replication were successful. Three biological replicates of OPCs and two biological replicates of oligodendrocytes (differentiated from two of the OPCs replicates), and two biological replicates of WT and tyw1Δ yeast, were used for tRNA sequencing. Two OPCs and oligodendrocytes replicates were used for the LC-MS/MS analyses. For the large-scale mRNA half-life analysis, three biological replicates of OPCs and two biological replicates of oligodendrocytes (that match two of the OPC replicates) were used. Two OPC replicates and the corresponding differentiated oligodendrocytes generated the ribosome footprints and paralleled RNA-seq samples. DESeq2 analyses performed for the differential expression of modification enzymes in our OPCs and differentiated oligodendrocytes included the RNA-Seq data that matches the ribosome footprints as well as the 0 hr time-point from the decay experiment, hence a final pool of five biological replicates for OPCs and 4 biological replicates for oligodendrocytes.<br>The RT-PCR from Figure 1c was repeated three times independently with similar results. The quantified Phe-GAA and Lys-UUU Northern blot analyses from Fig. 2h-i were repeated four times independently with similar results, and Fig. 3a and 3b were reproduced similarly on 3 different PAGE using three biological replicates. The HCl experiment in Fig. 3c and 3e is from 2 biological replicates of OPCs, oligodendrocytes, and astrocytes. The quantitation of Supplementary Figure 2 was obtained from independent PAGE of biological replicates, with the exact n indicated in the figure. The Northern blots in Supplementary Figure 5b and c were repeated twice independently with similar results. Supplementary Figure 5d was reproduced independently twice for Glu, Gln and Arg tRNAs.
Randomization	Randomization is not relevant to this study (we needed to identify variations between cell types).
Blinding	Blinding is not relevant to this study as there was no allocation of participants into groups (we needed to identify variations between cell types).

Reporting for specific materials, systems and methods

We require information from authors about some types of materials, experimental systems and methods used in many studies. Here, indicate whether each material, system or method listed is relevant to your study. If you are not sure if a list item applies to your research, read the appropriate section before selecting a response.

Materials & experimental systems

n/a	Involved in the study	
☐	☒	Antibodies
☐	☒	Eukaryotic cell lines
☒	☐	Palaeontology and archaeology
☐	☒	Animals and other organisms
☒	☐	Human research participants
☒	☐	Clinical data
☒	☐	Dual use research of concern

Methods

n/a	Involved in the study	
☒	☐	ChIP-seq
☒	☐	Flow cytometry
☒	☐	MRI-based neuroimaging

Antibodies

Antibodies used	Antibodies: anti-MBP (1:100, Abcam, ab7349), anti-O1 (1:50, CCF Hybridoma Core), anti-O4 (1:100, CCF Hybridoma Core), anti-OLIG2 (1.2mg/mL, Proteintech, 12999-1-AP), anti-GFAP (1:5000, Dako, Z033401-2), anti-PLP1 (1:1000, Lerner Research Institute Hybridoma Core), Alexa Fluor secondary antibodies (4µg/mL, Thermo Fisher), anti-CNPase (Abcam, ab6319), anti-TYW3 (1:1,000, MyBioSource, MBS150652), anti-ELP1 (1:250, Millipore Sigma, SAB2701068), anti-ELP3 (1:2,000, Abcam, ab190907) and anti-β-actin (1:5,000, Abcam, ab6276), anti-Mouse IgG (1:5,000, Abcam, ab216772), anti-Rabbit IgG (1:5,000, Abcam, ab216777)
Validation	Anti-MBP: RRID:AB_305869, citations including Baudouin et al. (2021), Glia, 69(8), 1916-1931<br>Anti-O1: reference Allan et al. (2021), Cell Stem Cell 28, 257-272.e11<br>Anti-O4: reference Allan et al. (2021), Cell Stem Cell 28, 257-272.e11<br>Anti-GFAP: citations including Zu et al. (2021), Aging cell, 20(8):e13437<br>Anti-PLP1: reference Elitt et al. (2020), Nature, 585(7825):397-403<br>Anti-OLIG2: RRID:AB_2157541, citations including Shen et al. (2021), Cell reports, 34(10), 10883<br>Anti-CNPase: RRID:AB_2082593, citations including Brooks et al. (2021), Nat Commun 12(1):2184<br>Anti-ELP3: citations including Zhang et al. (2021), J Biol Chem 297:101044<br>Anti-β-actin: RRID:AB_2223210, citations including Itai et al. (2021), Hum Mutat 42:66-7<br>Anti-Mouse IgG: RRID:AB_2857338, citations including Castro-Zavala et al. (2021), Prog Neuropsychopharmacol Biol Psychiatry, 109:110262<br>Anti-Rabbit IgG: citations including Zhou et al. (2021), Mol Med Rep 24(1):503

Eukaryotic cell lines

Policy information about cell lines

Cell line source(s)	Cells were obtained from mouse epiblast as in Najm et al., Nature, 522, 216–220 (2015)
Authentication	The cells were validated in Najm et al., Nature, 8, 957–962 (2011) and following publications (such as in Allan et al., Cell Stem Cell 28, 257-272.e11 (2021))
Mycoplasma contamination	All cells tested negative for mycoplasma contamination.
Commonly misidentified lines (See ICLAC register)	N/A

Animals and other organisms

Policy information about studies involving animals; ARRIVE guidelines recommended for reporting animal research

Laboratory animals	Mus musculus, 129S/SvEv, male, embryos E3.5 (The Jackson Laboratory)
Wild animals	The study did not involve wild animals.
Field-collected samples	The study did not involve samples collected from the field.
Ethics oversight	Mouse protocols were approved by Case Western Reserve University School of Medicine's Institutional Animal Care and Use Committee (IACUC).

Note that full information on the approval of the study protocol must also be provided in the manuscript.
