# Supplementary material for: Oligodendrocyte differentiation alters tRNA modifications and codon optimality-mediated mRNA decay
Source: Nat Commun. 2022 Aug 25;13:5003. doi: 10.1038/s41467-022-32766-3 (PMC9411196; doi:10.1038/s41467-022-32766-3)
Supplement: Supplementary file 5 — Source data [file 41467_2022_32766_MOESM5_ESM.zip › Source data/Mass_spectrometry.pdf]

Dataset: L:\masslynx\2020DS.PRO\JH20210215R.qld

Last Altered: Monday, February 15, 2021 09:49:11 Eastern Standard Time
Printed: Monday, February 15, 2021 09:51:12 Eastern Standard Time

Method: L:\masslynx\2020DS.PRO\MethDB\JH20210215targted.mdb 15 Feb 2021 09:36:24
Calibration: 15 Feb 2021 09:49:11

Compound name: m1A

#	Name	Type	Std. Conc	RT	Area	IS Area	Response	Primar...	pg/ul	%Dev
1	JH20210214STD1	Standard	10.000	1.22	3712305.750	84017.414	44.185	bb	9.3	-7.3
2	JH20210214STD2	Standard	5.000	1.27	2580021.750	92868.008	27.782	bb	5.8	16.0
3	JH20210214STD3	Standard	2.500	1.26	1565074.625	104337.430	15.000	bb	3.1	23.8
4	JH20210214STD4	Standard	1.250	1.26	849298.563	120161.008	7.068	bb	1.4	13.2
5	JH20210214STD5	Standard	0.625	1.27	457789.313	121686.063	3.762	bb	0.7	14.4
6	JH20210214STD6	Standard	0.313	1.27	244860.906	131911.875	1.856	bb	0.3	-0.3
7	JH20210214STD7	Standard	0.156	1.27	124365.297	137325.281	0.906	bb	0.1	-29.4
8	JH20210214STD8	Standard	0.078	1.27	63590.074	142116.281	0.447	bbX	0.0	-83.0
9	JH20210214Bla...	Blank		1.27	1125.910	18.381	61.254	bb	12.9	
10	JH20210214S1_1	Analyte		1.27	14306415.0...	74478.555	192.088	bb	40.6	
11	JH20210214S1_2	Analyte		1.26	14606366.0...	75776.961	192.755	bb	40.7	
12	JH20210214S2_1	Analyte		1.27	13558216.0...	76060.313	178.256	bb	37.7	
13	JH20210214S2_2	Analyte		1.26	13762308.0...	78468.984	175.385	bb	37.0	
14	JH20210214S3_1	Analyte		1.27	10772274.0...	82339.227	130.828	bb	27.6	
15	JH20210214S3_2	Analyte		1.27	10794668.0...	82824.008	130.333	bb	27.5	
16	JH20210214S4_1	Analyte		1.27	11529576.0...	76992.242	149.750	bb	31.6	
17	JH20210214S4_2	Analyte		1.27	11628682.0...	77198.688	150.633	bb	31.8	
18	JH20210214blan...	Blank		1.37	120.237			bb		
19	JH20210214SS...	Standard	10.000	1.26	6205513.500	149045.063	41.635	bb	8.7	-12.7
20	JH20210214SS...	Standard	5.000	1.27	3622535.000	148403.234	24.410	bb	5.1	1.7
21	JH20210214SS...	Standard	2.500	1.27	1933929.250	144501.766	13.383	bb	2.8	10.1
22	JH20210214SS...	Standard	1.250	1.26	1008937.750	148859.203	6.778	bb	1.4	8.3
23	JH20210214SS...	Standard	0.625	1.27	514307.031	148371.969	3.466	bb	0.7	4.4
24	JH20210214SS...	Standard	0.313	1.27	256848.063	146512.922	1.753	bb	0.3	-7.3
25	JH20210214SS...	Standard	0.156	1.27	129464.305	149499.781	0.866	bb	0.1	-34.8
26	JH20210214SS...	Standard	0.078	1.27	67048.828	156950.625	0.427	bbX	0.0	-88.5
27	JH20210214Bla...	Blank		1.25	161.162	14.561	11.068	bb	2.3	

Compound name: m1A
Correlation coefficient: r = 0.990927, r^2 = 0.981936
Calibration curve: 4.72346 * x + 0.384862
Response type: Internal Std (Ref 5), Area * (IS Conc. / IS Area)
Curve type: Linear, Origin: Exclude, Weighting: 1/x, Axis trans: None

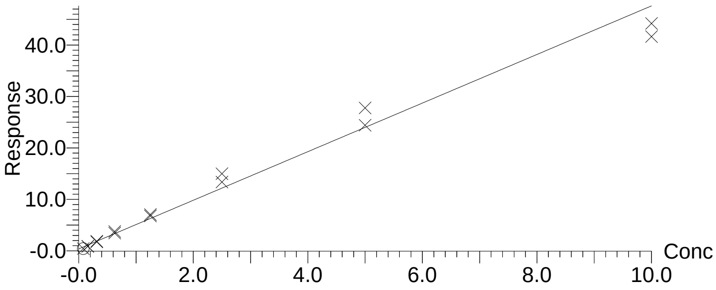

Dataset: L:\masslynx\2020DS.PRO\JH20210215R.qld

Last Altered: Monday, February 15, 2021 09:49:11 Eastern Standard Time
Printed: Monday, February 15, 2021 09:51:12 Eastern Standard Time

Compound name: Gm

#	Name	Type	Std. Conc	RT	Area	IS Area	Response	Primar...	pg/ul	%Dev
1	JH20210214STD1	Standard	10.000	4.13	601842.813	84017.414	7.163	bb	11.3	12.6
2	JH20210214STD2	Standard	5.000	4.15	285171.375	92868.008	3.071	bb	4.8	-3.3
3	JH20210214STD3	Standard	2.500	4.16	159300.500	104337.430	1.527	bb	2.4	-3.6
4	JH20210214STD4	Standard	1.250	4.14	86367.773	120161.008	0.719	bb	1.1	-8.7
5	JH20210214STD5	Standard	0.625	4.14	47057.184	121686.063	0.387	bb	0.6	-0.7
6	JH20210214STD6	Standard	0.313	4.14	25190.252	131911.875	0.191	bb	0.3	0.2
7	JH20210214STD7	Standard	0.156	4.14	12601.346	137325.281	0.092	bb	0.2	0.8
8	JH20210214STD8	Standard	0.078	4.14	6728.483	142116.281	0.047	bb	0.1	12.3
9	JH20210214Bla...	Blank		4.26	473.015	18.381	25.734	bb	40.4	
10	JH20210214S1_1	Analyte		4.14	457279.281	74478.555	6.140	bb	9.7	
11	JH20210214S1_2	Analyte		4.14	475174.000	75776.961	6.271	bb	9.9	
12	JH20210214S2_1	Analyte		4.14	527070.813	76060.313	6.930	bb	10.9	
13	JH20210214S2_2	Analyte		4.14	537294.688	78468.984	6.847	bb	10.8	
14	JH20210214S3_1	Analyte		4.14	386743.844	82339.227	4.697	bb	7.4	
15	JH20210214S3_2	Analyte		4.14	387657.000	82824.008	4.680	bb	7.4	
16	JH20210214S4_1	Analyte		4.14	428900.844	76992.242	5.571	bb	8.8	
17	JH20210214S4_2	Analyte		4.14	436872.125	77198.688	5.659	bb	8.9	
18	JH20210214blan...	Blank								
19	JH20210214SS...	Standard	10.000	4.14	889674.188	149045.063	5.969	bb	9.4	-6.2
20	JH20210214SS...	Standard	5.000	4.14	459387.375	148403.234	3.096	bb	4.9	-2.6
21	JH20210214SS...	Standard	2.500	4.14	222639.859	144501.766	1.541	bb	2.4	-2.7
22	JH20210214SS...	Standard	1.250	4.14	110730.813	148859.203	0.744	bb	1.2	-5.5
23	JH20210214SS...	Standard	0.625	4.14	55620.199	148371.969	0.375	bb	0.6	-3.7
24	JH20210214SS...	Standard	0.313	4.14	26854.059	146512.922	0.183	bb	0.3	-3.7
25	JH20210214SS...	Standard	0.156	4.14	14552.990	149499.781	0.097	bb	0.2	6.4
26	JH20210214SS...	Standard	0.078	4.14	7137.099	156950.625	0.045	bb	0.1	8.5
27	JH20210214Bla...	Blank				14.561				

Compound name: Gm
Correlation coefficient: r = 0.996757, r^2 = 0.993524
Calibration curve: 0.637066 * x + -0.0085284
Response type: Internal Std (Ref 5), Area * (IS Conc. / IS Area)
Curve type: Linear, Origin: Exclude, Weighting: 1/x, Axis trans: None

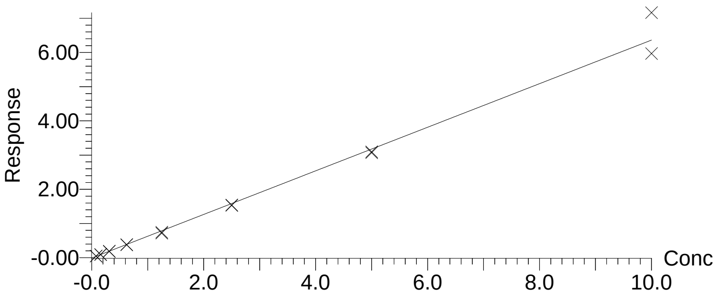

Dataset: L:\masslynx\2020DS.PRO\JH20210215R.qld

Last Altered: Monday, February 15, 2021 09:49:11 Eastern Standard Time
Printed: Monday, February 15, 2021 09:51:12 Eastern Standard Time

Compound name: mcm5U

#	Name	Type	Std. Conc	RT	Area	IS Area	Response	Primar...	pg/ul	%Dev
1	JH20210214STD1	Standard	10.000	4.22	176299.047	84017.414	2.098	bb	13.2	31.9
2	JH20210214STD2	Standard	5.000	4.26	75423.211	92868.008	0.812	bb	5.1	2.9
3	JH20210214STD3	Standard	2.500	4.27	40885.785	104337.430	0.392	bb	2.5	0.6
4	JH20210214STD4	Standard	1.250	4.24	21306.426	120161.008	0.177	bb	1.2	-6.2
5	JH20210214STD5	Standard	0.625	4.24	11329.688	121686.063	0.093	bb	0.6	3.3
6	JH20210214STD6	Standard	0.313	4.24	5996.057	131911.875	0.045	bb	0.3	11.2
7	JH20210214STD7	Standard	0.156	4.24	2731.084	137325.281	0.020	bb	0.2	20.0
8	JH20210214STD8	Standard	0.078	4.24	1357.850	142116.281	0.010	bbX	0.1	57.3
9	JH20210214Bla...	Blank		4.26	200.816	18.381	10.925	bb	68.4	
10	JH20210214S1_1	Analyte		4.24	17022.926	74478.555	0.229	bb	1.5	
11	JH20210214S1_2	Analyte		4.24	18473.699	75776.961	0.244	bb	1.6	
12	JH20210214S2_1	Analyte		4.24	18824.215	76060.313	0.247	bb	1.6	
13	JH20210214S2_2	Analyte		4.24	21260.393	78468.984	0.271	bb	1.8	
14	JH20210214S3_1	Analyte		4.24	8769.999	82339.227	0.107	bb	0.7	
15	JH20210214S3_2	Analyte		4.24	8361.593	82824.008	0.101	bb	0.7	
16	JH20210214S4_1	Analyte		4.24	9887.230	76992.242	0.128	bb	0.9	
17	JH20210214S4_2	Analyte		4.24	10270.523	77198.688	0.133	bb	0.9	
18	JH20210214blan...	Blank								
19	JH20210214SS...	Standard	10.000	4.24	193529.766	149045.063	1.298	bb	8.2	-18.2
20	JH20210214SS...	Standard	5.000	4.24	95218.109	148403.234	0.642	bb	4.1	-18.5
21	JH20210214SS...	Standard	2.500	4.24	46953.887	144501.766	0.325	bb	2.1	-16.2
22	JH20210214SS...	Standard	1.250	4.24	23743.340	148859.203	0.160	bb	1.1	-15.1
23	JH20210214SS...	Standard	0.625	4.24	12493.555	148371.969	0.084	bb	0.6	-5.6
24	JH20210214SS...	Standard	0.313	4.24	5775.967	146512.922	0.039	bb	0.3	-0.9
25	JH20210214SS...	Standard	0.156	4.24	2631.953	149499.781	0.018	bb	0.2	10.9
26	JH20210214SS...	Standard	0.078	4.24	1282.597	156950.625	0.008	bbX	0.1	46.3
27	JH20210214Bla...	Blank		4.24	105.661	14.561	7.256	bb	45.4	

Compound name: mcm5U
Correlation coefficient: r = 0.975383, r^2 = 0.951371
Calibration curve: 0.15988 * x + -0.0100966
Response type: Internal Std (Ref 5), Area * (IS Conc. / IS Area)
Curve type: Linear, Origin: Exclude, Weighting: 1/x, Axis trans: None

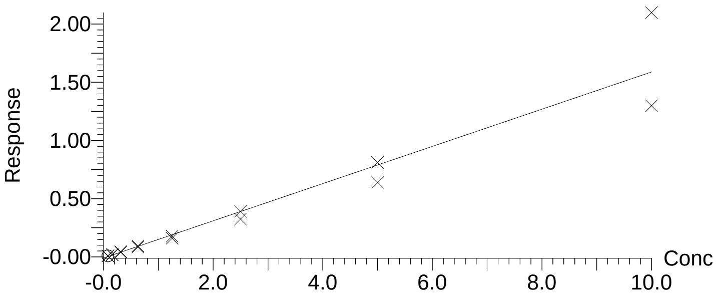

Dataset: L:\masslynx\2020DS.PRO\JH20210215R.qld

Last Altered: Monday, February 15, 2021 09:49:11 Eastern Standard Time
Printed: Monday, February 15, 2021 09:51:12 Eastern Standard Time

Compound name: mcm5s2U

#	Name	Type	Std. Conc	RT	Area	IS Area	Response	Primar...	pg/ul	%Dev
1	JH20210214STD1	Standard	10.000	4.90	129574.500	84017.414	1.542	bb	13.8	37.8
2	JH20210214STD2	Standard	5.000	4.96	39720.871	92868.008	0.428	bb	3.9	-22.5
3	JH20210214STD3	Standard	2.500	4.96	24946.402	104337.430	0.239	bb	2.2	-12.1
4	JH20210214STD4	Standard	1.250	4.93	15465.890	120161.008	0.129	bb	1.2	-2.7
5	JH20210214STD5	Standard	0.625	4.93	7704.117	121686.063	0.063	bb	0.6	1.6
6	JH20210214STD6	Standard	0.313	4.93	4011.439	131911.875	0.030	bb	0.3	9.6
7	JH20210214STD7	Standard	0.156	4.93	1978.575	137325.281	0.014	bb	0.2	28.1
8	JH20210214STD8	Standard	0.078	4.93	935.918	142116.281	0.007	bbX	0.1	67.1
9	JH20210214Bla...	Blank				18.381				
10	JH20210214S1_1	Analyte		4.93	56146.551	74478.555	0.754	bb	6.8	
11	JH20210214S1_2	Analyte		4.93	59781.984	75776.961	0.789	bb	7.1	
12	JH20210214S2_1	Analyte		4.93	61000.836	76060.313	0.802	bb	7.2	
13	JH20210214S2_2	Analyte		4.93	61476.793	78468.984	0.783	bb	7.0	
14	JH20210214S3_1	Analyte		4.93	27549.400	82339.227	0.335	bb	3.0	
15	JH20210214S3_2	Analyte		4.92	28886.730	82824.008	0.349	bb	3.2	
16	JH20210214S4_1	Analyte		4.93	29285.582	76992.242	0.380	MM	3.5	
17	JH20210214S4_2	Analyte		4.93	29896.760	77198.688	0.387	MM	3.5	
18	JH20210214blan...	Blank								
19	JH20210214SS...	Standard	10.000	4.93	146206.641	149045.063	0.981	bb	8.8	-12.1
20	JH20210214SS...	Standard	5.000	4.93	69395.617	148403.234	0.468	bb	4.2	-15.4
21	JH20210214SS...	Standard	2.500	4.93	35777.902	144501.766	0.248	bb	2.3	-9.1
22	JH20210214SS...	Standard	1.250	4.93	16956.041	148859.203	0.114	bb	1.1	-13.2
23	JH20210214SS...	Standard	0.625	4.93	8138.126	148371.969	0.055	bb	0.6	-10.5
24	JH20210214SS...	Standard	0.313	4.93	3918.624	146512.922	0.027	bb	0.3	-0.9
25	JH20210214SS...	Standard	0.156	4.93	1977.488	149499.781	0.013	bb	0.2	21.4
26	JH20210214SS...	Standard	0.078	4.93	983.679	156950.625	0.006	bbX	0.1	63.5
27	JH20210214Bla...	Blank				14.561				

Compound name: mcm5s2U
Correlation coefficient: r = 0.969289, r^2 = 0.939521
Calibration curve: 0.112484 * x + -0.00810238
Response type: Internal Std (Ref 5), Area * (IS Conc. / IS Area)
Curve type: Linear, Origin: Exclude, Weighting: 1/x, Axis trans: None

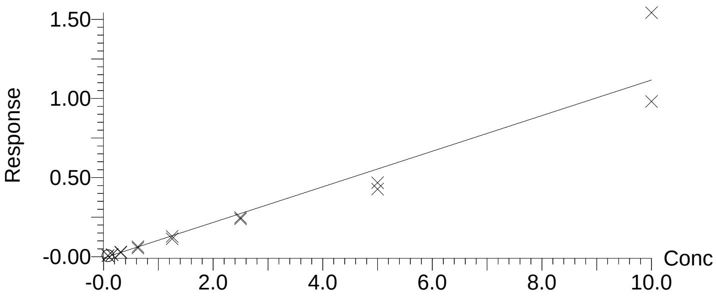

Dataset: L:\masslynx\2020DS.PRO\JH20210215R.qld

Last Altered: Monday, February 15, 2021 09:49:11 Eastern Standard Time
Printed: Monday, February 15, 2021 09:51:12 Eastern Standard Time

Compound name: G13C15N

#	Name	Type	Std. Conc	RT	Area	IS Area	Response	Primar...	Conc.	%Dev
1	JH20210214STD1	Standard	1.000	3.61	84017.414		84017.414	bbX	0.6	-38.3
2	JH20210214STD2	Standard	1.000	3.58	92868.008		92868.008	bb	0.7	-31.8
3	JH20210214STD3	Standard	1.000	3.58	104337.430		104337.430	bb	0.8	-23.4
4	JH20210214STD4	Standard	1.000	3.57	120161.008		120161.008	bb	0.9	-11.8
5	JH20210214STD5	Standard	1.000	3.58	121686.063		121686.063	bb	0.9	-10.6
6	JH20210214STD6	Standard	1.000	3.57	131911.875		131911.875	bb	1.0	-3.1
7	JH20210214STD7	Standard	1.000	3.58	137325.281		137325.281	bb	1.0	0.8
8	JH20210214STD8	Standard	1.000	3.58	142116.281		142116.281	bb	1.0	4.4
9	JH20210214Bla...	Blank	1.000	3.57	18.381		18.381	bb	0.0	-100.0
10	JH20210214S1_1	Analyte	1.000	3.58	74478.555		74478.555	bb	0.5	-45.3
11	JH20210214S1_2	Analyte	1.000	3.57	75776.961		75776.961	bb	0.6	-44.4
12	JH20210214S2_1	Analyte	1.000	3.57	76060.313		76060.313	bb	0.6	-44.1
13	JH20210214S2_2	Analyte	1.000	3.57	78468.984		78468.984	bb	0.6	-42.4
14	JH20210214S3_1	Analyte	1.000	3.57	82339.227		82339.227	bb	0.6	-39.5
15	JH20210214S3_2	Analyte	1.000	3.57	82824.008		82824.008	bb	0.6	-39.2
16	JH20210214S4_1	Analyte	1.000	3.57	76992.242		76992.242	bb	0.6	-43.5
17	JH20210214S4_2	Analyte	1.000	3.57	77198.688		77198.688	bb	0.6	-43.3
18	JH20210214blan...	Blank	1.000							
19	JH20210214SS...	Standard	1.000	3.57	149045.063		149045.063	bb	1.1	9.5
20	JH20210214SS...	Standard	1.000	3.57	148403.234		148403.234	bb	1.1	9.0
21	JH20210214SS...	Standard	1.000	3.57	144501.766		144501.766	bb	1.1	6.1
22	JH20210214SS...	Standard	1.000	3.57	148859.203		148859.203	bb	1.1	9.3
23	JH20210214SS...	Standard	1.000	3.58	148371.969		148371.969	bb	1.1	9.0
24	JH20210214SS...	Standard	1.000	3.57	146512.922		146512.922	bb	1.1	7.6
25	JH20210214SS...	Standard	1.000	3.57	149499.781		149499.781	bb	1.1	9.8
26	JH20210214SS...	Standard	1.000	3.58	156950.625		156950.625	bb	1.2	15.3
27	JH20210214Bla...	Blank	1.000	3.57	14.561		14.561	bb	0.0	-100.0

Compound name: G13C15N
Response Factor: 136170
RRF SD: 18555.9, % Relative SD: 13.627
Response type: External Std, Area
Curve type: RF

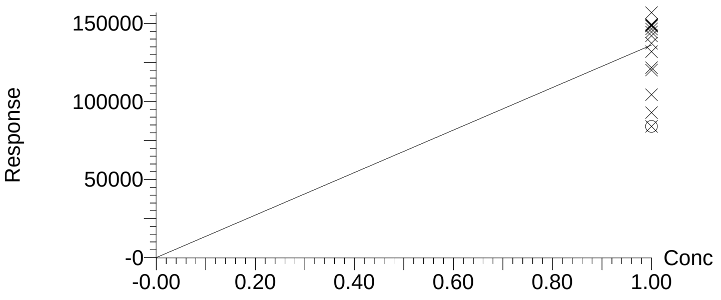

Dataset: L:\masslynx\2020DS.PRO\JH20210215R.qld

Last Altered: Monday, February 15, 2021 09:49:11 Eastern Standard Time
Printed: Monday, February 15, 2021 09:51:12 Eastern Standard Time

Method: L:\masslynx\2020DS.PRO\MethDB\JH20210215targted.mdb 15 Feb 2021 09:36:24
Calibration: 15 Feb 2021 09:49:11

Name: JH20210214STD1, Date: 14-Feb-2021, Time: 14:25:30, ID: STD1, Description: 10 pg/ul+ISGm/m1A/mcm5U/mcm5s2U

#	Name	Trace	RT	Area	IS Area	Response	Primar...	Conc.	%Dev
1	m1A	282 > 150	1.22	3712305.750	84017.414	44.185	bb	9.3	-7.3
2	Gm	298.075 > 152.088	4.13	601842.813	84017.414	7.163	bb	11.3	12.6
3	mcm5U	317.118 > 185.091	4.22	176299.047	84017.414	2.098	bb	13.2	31.9
4	mcm5s2U	333.032 > 201.004	4.90	129574.500	84017.414	1.542	bb	13.8	37.8
5	G13C15N	299.162 > 162.059	3.61	84017.414		84017.414	bbX	0.6	-38.3

Name: JH20210214STD2, Date: 14-Feb-2021, Time: 14:33:18, ID: STD2, Description: 5

#	Name	Trace	RT	Area	IS Area	Response	Primar...	Conc.	%Dev
1	m1A	282 > 150	1.27	2580021.750	92868.008	27.782	bb	5.8	16.0
2	Gm	298.075 > 152.088	4.15	285171.375	92868.008	3.071	bb	4.8	-3.3
3	mcm5U	317.118 > 185.091	4.26	75423.211	92868.008	0.812	bb	5.1	2.9
4	mcm5s2U	333.032 > 201.004	4.96	39720.871	92868.008	0.428	bb	3.9	-22.5
5	G13C15N	299.162 > 162.059	3.58	92868.008		92868.008	bb	0.7	-31.8

Name: JH20210214STD3, Date: 14-Feb-2021, Time: 14:41:07, ID: STD3, Description: 2.5

#	Name	Trace	RT	Area	IS Area	Response	Primar...	Conc.	%Dev
1	m1A	282 > 150	1.26	1565074.625	104337.430	15.000	bb	3.1	23.8
2	Gm	298.075 > 152.088	4.16	159300.500	104337.430	1.527	bb	2.4	-3.6
3	mcm5U	317.118 > 185.091	4.27	40885.785	104337.430	0.392	bb	2.5	0.6
4	mcm5s2U	333.032 > 201.004	4.96	24946.402	104337.430	0.239	bb	2.2	-12.1
5	G13C15N	299.162 > 162.059	3.58	104337.430		104337.430	bb	0.8	-23.4

Name: JH20210214STD4, Date: 14-Feb-2021, Time: 14:48:56, ID: STD4, Description: 1.25

#	Name	Trace	RT	Area	IS Area	Response	Primar...	Conc.	%Dev
1	m1A	282 > 150	1.26	849298.563	120161.008	7.068	bb	1.4	13.2
2	Gm	298.075 > 152.088	4.14	86367.773	120161.008	0.719	bb	1.1	-8.7
3	mcm5U	317.118 > 185.091	4.24	21306.426	120161.008	0.177	bb	1.2	-6.2
4	mcm5s2U	333.032 > 201.004	4.93	15465.890	120161.008	0.129	bb	1.2	-2.7
5	G13C15N	299.162 > 162.059	3.57	120161.008		120161.008	bb	0.9	-11.8

Name: JH20210214STD5, Date: 14-Feb-2021, Time: 14:56:45, ID: STD5, Description: 0.625

#	Name	Trace	RT	Area	IS Area	Response	Primar...	Conc.	%Dev
1	m1A	282 > 150	1.27	457789.313	121686.063	3.762	bb	0.7	14.4
2	Gm	298.075 > 152.088	4.14	47057.184	121686.063	0.387	bb	0.6	-0.7
3	mcm5U	317.118 > 185.091	4.24	11329.688	121686.063	0.093	bb	0.6	3.3
4	mcm5s2U	333.032 > 201.004	4.93	7704.117	121686.063	0.063	bb	0.6	1.6
5	G13C15N	299.162 > 162.059	3.58	121686.063		121686.063	bb	0.9	-10.6

Dataset: L:\masslynx\2020DS.PRO\JH20210215R.qld

Last Altered: Monday, February 15, 2021 09:49:11 Eastern Standard Time
Printed: Monday, February 15, 2021 09:51:12 Eastern Standard Time

Name: JH20210214STD6, Date: 14-Feb-2021, Time: 15:04:33, ID: STD6, Description: 0.3125

#	Name	Trace	RT	Area	IS Area	Response	Primar...	Conc.	%Dev
1	m1A	282 > 150	1.27	244860.906	131911.875	1.856	bb	0.3	-0.3
2	Gm	298.075 > 152.088	4.14	25190.252	131911.875	0.191	bb	0.3	0.2
3	mcm5U	317.118 > 185.091	4.24	5996.057	131911.875	0.045	bb	0.3	11.2
4	mcm5s2U	333.032 > 201.004	4.93	4011.439	131911.875	0.030	bb	0.3	9.6
5	G13C15N	299.162 > 162.059	3.57	131911.875		131911.875	bb	1.0	-3.1

Name: JH20210214STD7, Date: 14-Feb-2021, Time: 15:12:22, ID: STD7, Description: 0.15625

#	Name	Trace	RT	Area	IS Area	Response	Primar...	Conc.	%Dev
1	m1A	282 > 150	1.27	124365.297	137325.281	0.906	bb	0.1	-29.4
2	Gm	298.075 > 152.088	4.14	12601.346	137325.281	0.092	bb	0.2	0.8
3	mcm5U	317.118 > 185.091	4.24	2731.084	137325.281	0.020	bb	0.2	20.0
4	mcm5s2U	333.032 > 201.004	4.93	1978.575	137325.281	0.014	bb	0.2	28.1
5	G13C15N	299.162 > 162.059	3.58	137325.281		137325.281	bb	1.0	0.8

Name: JH20210214STD8, Date: 14-Feb-2021, Time: 15:20:10, ID: STD8, Description: 0.078125

#	Name	Trace	RT	Area	IS Area	Response	Primar...	Conc.	%Dev
1	m1A	282 > 150	1.27	63590.074	142116.281	0.447	bbX	0.0	-83.0
2	Gm	298.075 > 152.088	4.14	6728.483	142116.281	0.047	bb	0.1	12.3
3	mcm5U	317.118 > 185.091	4.24	1357.850	142116.281	0.010	bbX	0.1	57.3
4	mcm5s2U	333.032 > 201.004	4.93	935.918	142116.281	0.007	bbX	0.1	67.1
5	G13C15N	299.162 > 162.059	3.58	142116.281		142116.281	bb	1.0	4.4

Name: JH20210214Blank1, Date: 14-Feb-2021, Time: 15:27:59, ID: blk, Description: blank

#	Name	Trace	RT	Area	IS Area	Response	Primar...	Conc.	%Dev
1	m1A	282 > 150	1.27	1125.910	18.381	61.254	bb	12.9	
2	Gm	298.075 > 152.088	4.26	473.015	18.381	25.734	bb	40.4	
3	mcm5U	317.118 > 185.091	4.26	200.816	18.381	10.925	bb	68.4	
4	mcm5s2U	333.032 > 201.004			18.381				
5	G13C15N	299.162 > 162.059	3.57	18.381		18.381	bb	0.0	-100.0

Name: JH20210214S1_1, Date: 14-Feb-2021, Time: 15:35:48, ID: S1, Description: A+IS

#	Name	Trace	RT	Area	IS Area	Response	Primar...	Conc.	%Dev
1	m1A	282 > 150	1.27	14306415.0...	74478.555	192.088	bb	40.6	
2	Gm	298.075 > 152.088	4.14	457279.281	74478.555	6.140	bb	9.7	
3	mcm5U	317.118 > 185.091	4.24	17022.926	74478.555	0.229	bb	1.5	
4	mcm5s2U	333.032 > 201.004	4.93	56146.551	74478.555	0.754	bb	6.8	
5	G13C15N	299.162 > 162.059	3.58	74478.555		74478.555	bb	0.5	-45.3

Name: JH20210214S1_2, Date: 14-Feb-2021, Time: 15:43:37, ID: S2, Description: A+IS

#	Name	Trace	RT	Area	IS Area	Response	Primar...	Conc.	%Dev
1	m1A	282 > 150	1.26	14606366.0...	75776.961	192.755	bb	40.7	
2	Gm	298.075 > 152.088	4.14	475174.000	75776.961	6.271	bb	9.9	
3	mcm5U	317.118 > 185.091	4.24	18473.699	75776.961	0.244	bb	1.6	

Dataset: L:\masslynx\2020DS.PRO\JH20210215R.qld

Last Altered: Monday, February 15, 2021 09:49:11 Eastern Standard Time
Printed: Monday, February 15, 2021 09:51:12 Eastern Standard Time

Name: JH20210214S1_2, Date: 14-Feb-2021, Time: 15:43:37, ID: S2, Description: A+IS

#	Name	Trace	RT	Area	IS Area	Response	Primar...	Conc.	%Dev
4	mcm5s2U	333.032 > 201.004	4.93	59781.984	75776.961	0.789	bb	7.1	
5	G13C15N	299.162 > 162.059	3.57	75776.961		75776.961	bb	0.6	-44.4

Name: JH20210214S2_1, Date: 14-Feb-2021, Time: 15:51:25, ID: S3, Description: B+IS

#	Name	Trace	RT	Area	IS Area	Response	Primar...	Conc.	%Dev
1	m1A	282 > 150	1.27	13558216.0...	76060.313	178.256	bb	37.7	
2	Gm	298.075 > 152.088	4.14	527070.813	76060.313	6.930	bb	10.9	
3	mcm5U	317.118 > 185.091	4.24	18824.215	76060.313	0.247	bb	1.6	
4	mcm5s2U	333.032 > 201.004	4.93	61000.836	76060.313	0.802	bb	7.2	
5	G13C15N	299.162 > 162.059	3.57	76060.313		76060.313	bb	0.6	-44.1

Name: JH20210214S2_2, Date: 14-Feb-2021, Time: 15:59:14, ID: S4, Description: B+IS

#	Name	Trace	RT	Area	IS Area	Response	Primar...	Conc.	%Dev
1	m1A	282 > 150	1.26	13762308.0...	78468.984	175.385	bb	37.0	
2	Gm	298.075 > 152.088	4.14	537294.688	78468.984	6.847	bb	10.8	
3	mcm5U	317.118 > 185.091	4.24	21260.393	78468.984	0.271	bb	1.8	
4	mcm5s2U	333.032 > 201.004	4.93	61476.793	78468.984	0.783	bb	7.0	
5	G13C15N	299.162 > 162.059	3.57	78468.984		78468.984	bb	0.6	-42.4

Name: JH20210214S3_1, Date: 14-Feb-2021, Time: 16:07:03, ID: S5, Description: C+IS

#	Name	Trace	RT	Area	IS Area	Response	Primar...	Conc.	%Dev
1	m1A	282 > 150	1.27	10772274.0...	82339.227	130.828	bb	27.6	
2	Gm	298.075 > 152.088	4.14	386743.844	82339.227	4.697	bb	7.4	
3	mcm5U	317.118 > 185.091	4.24	8769.999	82339.227	0.107	bb	0.7	
4	mcm5s2U	333.032 > 201.004	4.93	27549.400	82339.227	0.335	bb	3.0	
5	G13C15N	299.162 > 162.059	3.57	82339.227		82339.227	bb	0.6	-39.5

Name: JH20210214S3_2, Date: 14-Feb-2021, Time: 16:14:51, ID: S6, Description: C+IS

#	Name	Trace	RT	Area	IS Area	Response	Primar...	Conc.	%Dev
1	m1A	282 > 150	1.27	10794668.0...	82824.008	130.333	bb	27.5	
2	Gm	298.075 > 152.088	4.14	387657.000	82824.008	4.680	bb	7.4	
3	mcm5U	317.118 > 185.091	4.24	8361.593	82824.008	0.101	bb	0.7	
4	mcm5s2U	333.032 > 201.004	4.92	28886.730	82824.008	0.349	bb	3.2	
5	G13C15N	299.162 > 162.059	3.57	82824.008		82824.008	bb	0.6	-39.2

Name: JH20210214S4_1, Date: 14-Feb-2021, Time: 16:22:40, ID: S7, Description: D+IS

#	Name	Trace	RT	Area	IS Area	Response	Primar...	Conc.	%Dev
1	m1A	282 > 150	1.27	11529576.0...	76992.242	149.750	bb	31.6	
2	Gm	298.075 > 152.088	4.14	428900.844	76992.242	5.571	bb	8.8	
3	mcm5U	317.118 > 185.091	4.24	9887.230	76992.242	0.128	bb	0.9	
4	mcm5s2U	333.032 > 201.004	4.93	29285.582	76992.242	0.380	MM	3.5	
5	G13C15N	299.162 > 162.059	3.57	76992.242		76992.242	bb	0.6	-43.5

Dataset: L:\masslynx\2020DS.PRO\JH20210215R.qld

Last Altered: Monday, February 15, 2021 09:49:11 Eastern Standard Time
Printed: Monday, February 15, 2021 09:51:12 Eastern Standard Time

Name: JH20210214S4_2, Date: 14-Feb-2021, Time: 16:30:29, ID: S8, Description: D+IS

#	Name	Trace	RT	Area	IS Area	Response	Primar...	Conc.	%Dev
1	m1A	282 > 150	1.27	11628682.0...	77198.688	150.633	bb	31.8	
2	Gm	298.075 > 152.088	4.14	436872.125	77198.688	5.659	bb	8.9	
3	mcm5U	317.118 > 185.091	4.24	10270.523	77198.688	0.133	bb	0.9	
4	mcm5s2U	333.032 > 201.004	4.93	29896.760	77198.688	0.387	MM	3.5	
5	G13C15N	299.162 > 162.059	3.57	77198.688		77198.688	bb	0.6	-43.3

Name: JH20210214blank2, Date: 14-Feb-2021, Time: 16:38:17, ID: blk, Description: blank

#	Name	Trace	RT	Area	IS Area	Response	Primar...	Conc.	%Dev
1	m1A	282 > 150	1.37	120.237			bb		
2	Gm	298.075 > 152.088							
3	mcm5U	317.118 > 185.091							
4	mcm5s2U	333.032 > 201.004							
5	G13C15N	299.162 > 162.059							

Name: JH20210214SSTD1, Date: 14-Feb-2021, Time: 16:46:06, ID: STD1, Description: 10 pg/ul+ISGm/m1A/mcm5U/mcm5s2U

#	Name	Trace	RT	Area	IS Area	Response	Primar...	Conc.	%Dev
1	m1A	282 > 150	1.26	6205513.500	149045.063	41.635	bb	8.7	-12.7
2	Gm	298.075 > 152.088	4.14	889674.188	149045.063	5.969	bb	9.4	-6.2
3	mcm5U	317.118 > 185.091	4.24	193529.766	149045.063	1.298	bb	8.2	-18.2
4	mcm5s2U	333.032 > 201.004	4.93	146206.641	149045.063	0.981	bb	8.8	-12.1
5	G13C15N	299.162 > 162.059	3.57	149045.063		149045.063	bb	1.1	9.5

Name: JH20210214SSTD2, Date: 14-Feb-2021, Time: 16:53:55, ID: STD2, Description: 5

#	Name	Trace	RT	Area	IS Area	Response	Primar...	Conc.	%Dev
1	m1A	282 > 150	1.27	3622535.000	148403.234	24.410	bb	5.1	1.7
2	Gm	298.075 > 152.088	4.14	459387.375	148403.234	3.096	bb	4.9	-2.6
3	mcm5U	317.118 > 185.091	4.24	95218.109	148403.234	0.642	bb	4.1	-18.5
4	mcm5s2U	333.032 > 201.004	4.93	69395.617	148403.234	0.468	bb	4.2	-15.4
5	G13C15N	299.162 > 162.059	3.57	148403.234		148403.234	bb	1.1	9.0

Name: JH20210214SSTD3, Date: 14-Feb-2021, Time: 17:01:43, ID: STD3, Description: 2.5

#	Name	Trace	RT	Area	IS Area	Response	Primar...	Conc.	%Dev
1	m1A	282 > 150	1.27	1933929.250	144501.766	13.383	bb	2.8	10.1
2	Gm	298.075 > 152.088	4.14	222639.859	144501.766	1.541	bb	2.4	-2.7
3	mcm5U	317.118 > 185.091	4.24	46953.887	144501.766	0.325	bb	2.1	-16.2
4	mcm5s2U	333.032 > 201.004	4.93	35777.902	144501.766	0.248	bb	2.3	-9.1
5	G13C15N	299.162 > 162.059	3.57	144501.766		144501.766	bb	1.1	6.1

Name: JH20210214SSTD4, Date: 14-Feb-2021, Time: 17:09:32, ID: STD4, Description: 1.25

#	Name	Trace	RT	Area	IS Area	Response	Primar...	Conc.	%Dev
1	m1A	282 > 150	1.26	1008937.750	148859.203	6.778	bb	1.4	8.3
2	Gm	298.075 > 152.088	4.14	110730.813	148859.203	0.744	bb	1.2	-5.5
3	mcm5U	317.118 > 185.091	4.24	23743.340	148859.203	0.160	bb	1.1	-15.1

Dataset: L:\masslynx\2020DS.PRO\JH20210215R.qld

Last Altered: Monday, February 15, 2021 09:49:11 Eastern Standard Time
Printed: Monday, February 15, 2021 09:51:12 Eastern Standard Time

Name: JH20210214SSTD4, Date: 14-Feb-2021, Time: 17:09:32, ID: STD4, Description: 1.25

#	Name	Trace	RT	Area	IS Area	Response	Primar...	Conc.	%Dev
4	mcm5s2U	333.032 > 201.004	4.93	16956.041	148859.203	0.114	bb	1.1	-13.2
5	G13C15N	299.162 > 162.059	3.57	148859.203		148859.203	bb	1.1	9.3

Name: JH20210214SSTD5, Date: 14-Feb-2021, Time: 17:17:21, ID: STD5, Description: 0.625

#	Name	Trace	RT	Area	IS Area	Response	Primar...	Conc.	%Dev
1	m1A	282 > 150	1.27	514307.031	148371.969	3.466	bb	0.7	4.4
2	Gm	298.075 > 152.088	4.14	55620.199	148371.969	0.375	bb	0.6	-3.7
3	mcm5U	317.118 > 185.091	4.24	12493.555	148371.969	0.084	bb	0.6	-5.6
4	mcm5s2U	333.032 > 201.004	4.93	8138.126	148371.969	0.055	bb	0.6	-10.5
5	G13C15N	299.162 > 162.059	3.58	148371.969		148371.969	bb	1.1	9.0

Name: JH20210214SSTD6, Date: 14-Feb-2021, Time: 17:25:09, ID: STD6, Description: 0.3125

#	Name	Trace	RT	Area	IS Area	Response	Primar...	Conc.	%Dev
1	m1A	282 > 150	1.27	256848.063	146512.922	1.753	bb	0.3	-7.3
2	Gm	298.075 > 152.088	4.14	26854.059	146512.922	0.183	bb	0.3	-3.7
3	mcm5U	317.118 > 185.091	4.24	5775.967	146512.922	0.039	bb	0.3	-0.9
4	mcm5s2U	333.032 > 201.004	4.93	3918.624	146512.922	0.027	bb	0.3	-0.9
5	G13C15N	299.162 > 162.059	3.57	146512.922		146512.922	bb	1.1	7.6

Name: JH20210214SSTD7, Date: 14-Feb-2021, Time: 17:32:58, ID: STD7, Description: 0.15625

#	Name	Trace	RT	Area	IS Area	Response	Primar...	Conc.	%Dev
1	m1A	282 > 150	1.27	129464.305	149499.781	0.866	bb	0.1	-34.8
2	Gm	298.075 > 152.088	4.14	14552.990	149499.781	0.097	bb	0.2	6.4
3	mcm5U	317.118 > 185.091	4.24	2631.953	149499.781	0.018	bb	0.2	10.9
4	mcm5s2U	333.032 > 201.004	4.93	1977.488	149499.781	0.013	bb	0.2	21.4
5	G13C15N	299.162 > 162.059	3.57	149499.781		149499.781	bb	1.1	9.8

Name: JH20210214SSTD8, Date: 14-Feb-2021, Time: 17:40:47, ID: STD8, Description: 0.078125

#	Name	Trace	RT	Area	IS Area	Response	Primar...	Conc.	%Dev
1	m1A	282 > 150	1.27	67048.828	156950.625	0.427	bbX	0.0	-88.5
2	Gm	298.075 > 152.088	4.14	7137.099	156950.625	0.045	bb	0.1	8.5
3	mcm5U	317.118 > 185.091	4.24	1282.597	156950.625	0.008	bbX	0.1	46.3
4	mcm5s2U	333.032 > 201.004	4.93	983.679	156950.625	0.006	bbX	0.1	63.5
5	G13C15N	299.162 > 162.059	3.58	156950.625		156950.625	bb	1.2	15.3

Name: JH20210214Blank3, Date: 14-Feb-2021, Time: 17:48:35, ID: blk, Description: blank

#	Name	Trace	RT	Area	IS Area	Response	Primar...	Conc.	%Dev
1	m1A	282 > 150	1.25	161.162	14.561	11.068	bb	2.3	
2	Gm	298.075 > 152.088			14.561				
3	mcm5U	317.118 > 185.091	4.24	105.661	14.561	7.256	bb	45.4	
4	mcm5s2U	333.032 > 201.004			14.561				
5	G13C15N	299.162 > 162.059	3.57	14.561		14.561	bb	0.0	-100.0

Dataset: L:\masslynx\2020DS.PRO\JH20210215R.qld

Last Altered: Monday, February 15, 2021 09:49:11 Eastern Standard Time
Printed: Monday, February 15, 2021 09:51:12 Eastern Standard Time

Method: L:\masslynx\2020DS.PRO\MethDB\JH20210215targted.mdb 15 Feb 2021 09:36:24
Calibration: 15 Feb 2021 09:49:11

No totals to report

Dataset: L:\masslynx\2020DS.PRO\JH20210215R.qld

Last Altered: Monday, February 15, 2021 09:49:11 Eastern Standard Time
Printed: Monday, February 15, 2021 09:51:12 Eastern Standard Time

Method: L:\masslynx\2020DS.PRO\MethDB\JH20210215targted.mdb 15 Feb 2021 09:36:24
Calibration: 15 Feb 2021 09:49:11

Compound name: m1A
Correlation coefficient: r = 0.990927, r^2 = 0.981936
Calibration curve: 4.72346 * x + 0.384862
Response type: Internal Std (Ref 5), Area * (IS Conc. / IS Area)
Curve type: Linear, Origin: Exclude, Weighting: 1/x, Axis trans: None

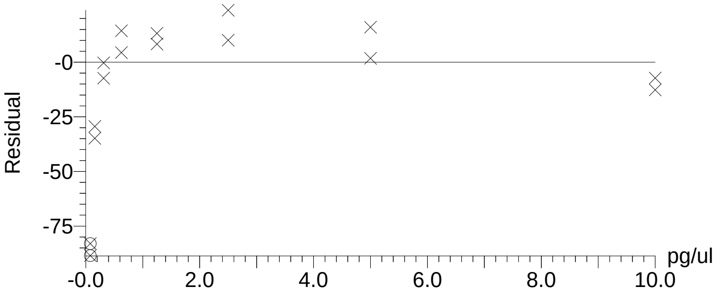

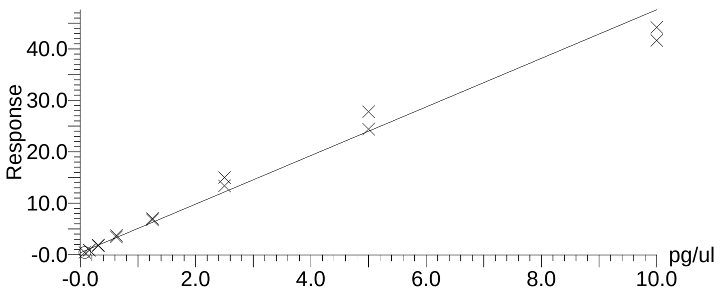

Compound name: Gm
Correlation coefficient: r = 0.996757, r^2 = 0.993524
Calibration curve: 0.637066 * x + -0.0085284
Response type: Internal Std (Ref 5), Area * (IS Conc. / IS Area)
Curve type: Linear, Origin: Exclude, Weighting: 1/x, Axis trans: None

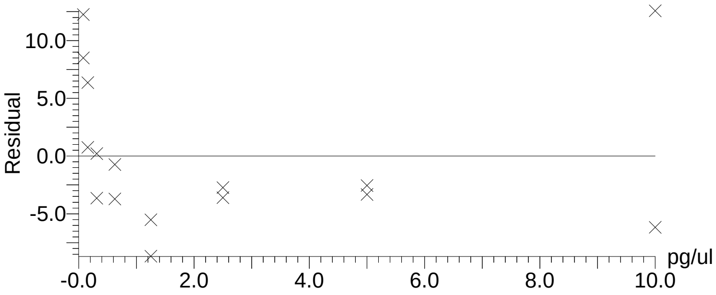

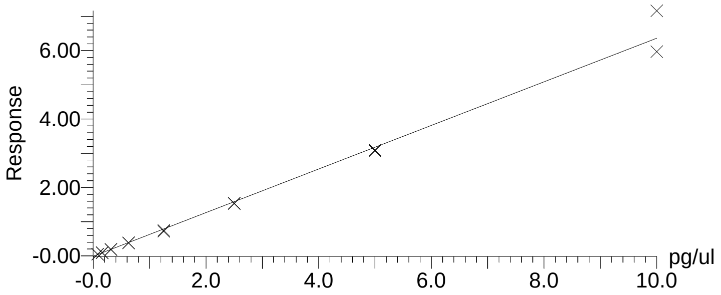

Compound name: mcm5U
Correlation coefficient: r = 0.975383, r^2 = 0.951371
Calibration curve: 0.15988 * x + -0.0100966
Response type: Internal Std (Ref 5), Area * (IS Conc. / IS Area)
Curve type: Linear, Origin: Exclude, Weighting: 1/x, Axis trans: None

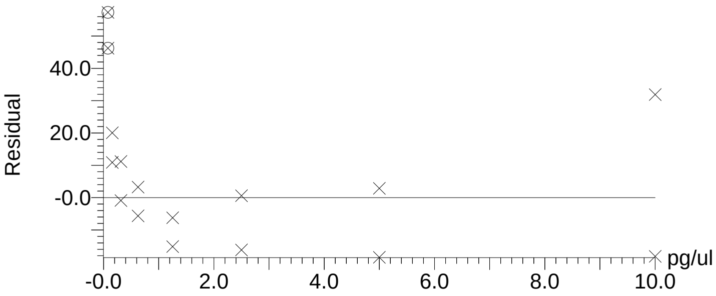

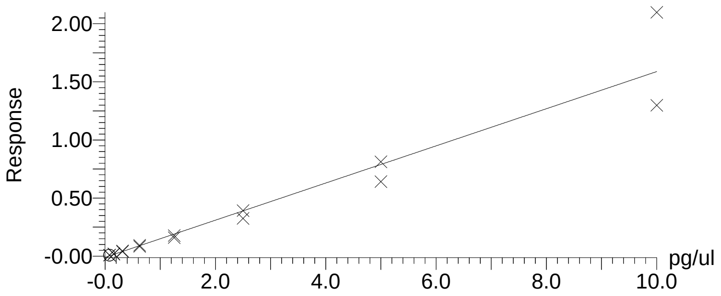

Dataset: L:\masslynx\2020DS.PRO\JH20210215R.qld

Last Altered: Monday, February 15, 2021 09:49:11 Eastern Standard Time
Printed: Monday, February 15, 2021 09:51:12 Eastern Standard Time

Compound name: mcm5s2U
Correlation coefficient: r = 0.969289, r^2 = 0.939521
Calibration curve: 0.112484 * x + -0.00810238
Response type: Internal Std (Ref 5), Area * (IS Conc. / IS Area)
Curve type: Linear, Origin: Exclude, Weighting: 1/x, Axis trans: None

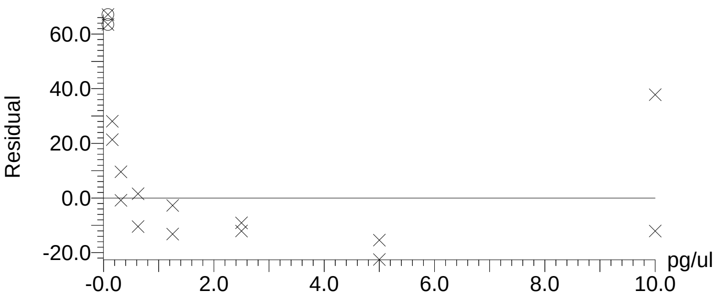

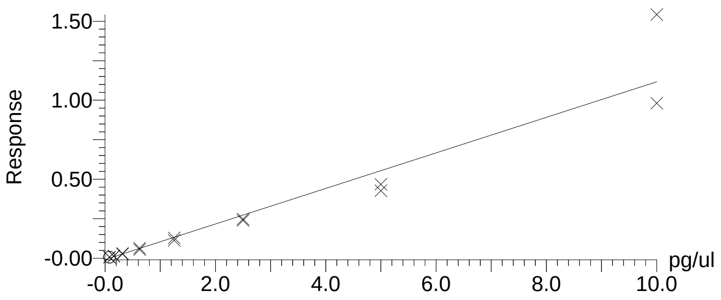

Compound name: G13C15N
Response Factor: 136170
RRF SD: 18555.9, % Relative SD: 13.627
Response type: External Std, Area
Curve type: RF

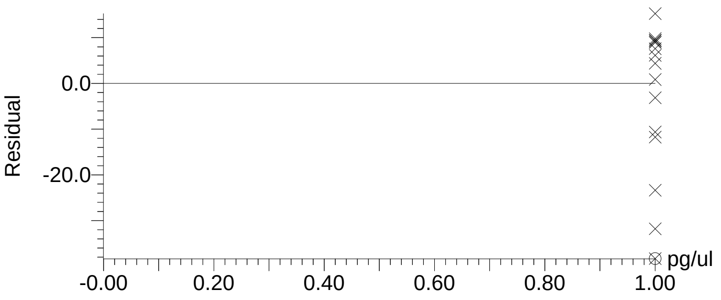

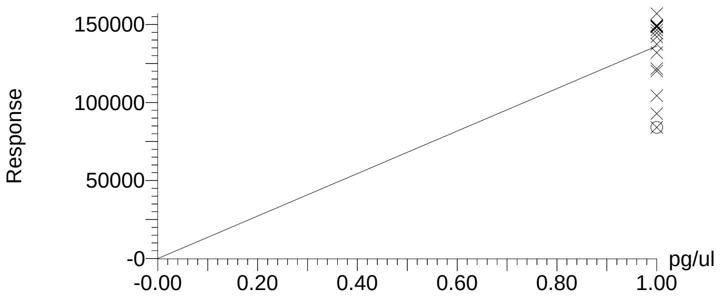

Dataset: L:\masslynx\2020DS.PRO\JH20210215R.qld

Last Altered: Monday, February 15, 2021 09:49:11 Eastern Standard Time
Printed: Monday, February 15, 2021 09:51:12 Eastern Standard Time

Method: L:\masslynx\2020DS.PRO\MethDB\JH20210215targted.mdb 15 Feb 2021 09:36:24
Calibration: 15 Feb 2021 09:49:11

Name: JH20210214STD1, Date: 14-Feb-2021, Time: 14:25:30, ID: STD1, Description: 10 pg/ul+ISGm/m1A/mcm5U/mcm5s2U

m1A

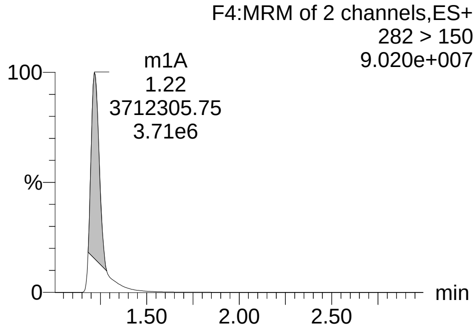

Gm

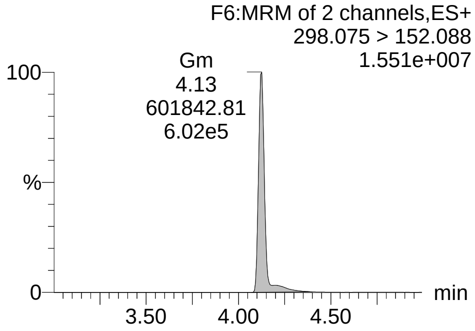

mcm5U

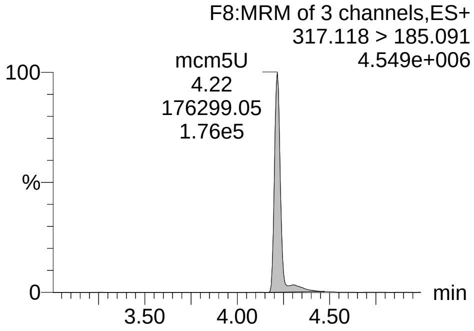

mcm5s2U

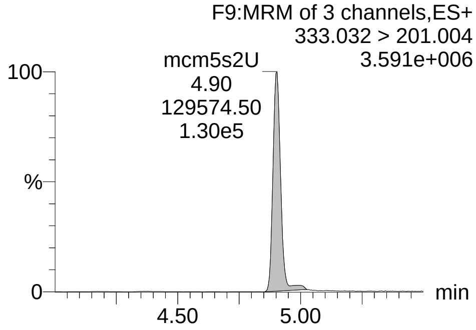

G13C15N

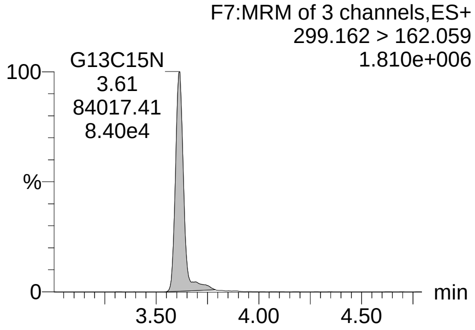

#	Name	Trace	RT	Area	IS Area	Response	Primar...	Conc.	%Dev
1	m1A	282 > 150	1.22	3712305.750	84017.414	44.185	bb	9.3	-7.3
2	Gm	298.075 > 152.088	4.13	601842.813	84017.414	7.163	bb	11.3	12.6
3	mcm5U	317.118 > 185.091	4.22	176299.047	84017.414	2.098	bb	13.2	31.9
4	mcm5s2U	333.032 > 201.004	4.90	129574.500	84017.414	1.542	bb	13.8	37.8
5	G13C15N	299.162 > 162.059	3.61	84017.414		84017.414	bbX	0.6	-38.3

Name: JH20210214STD2, Date: 14-Feb-2021, Time: 14:33:18, ID: STD2, Description: 5

m1A

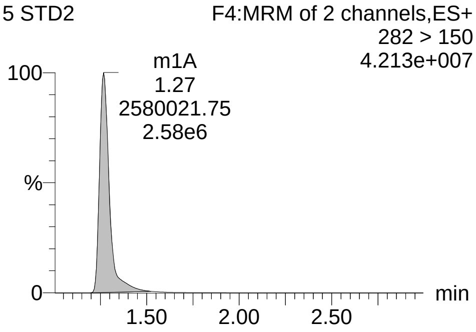

Gm

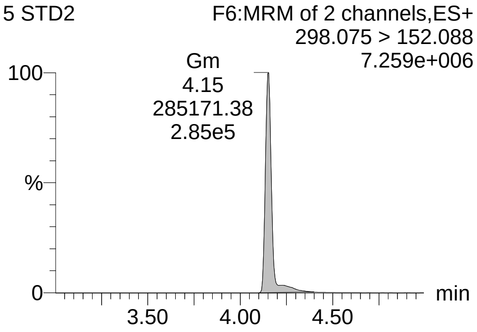

mcm5U

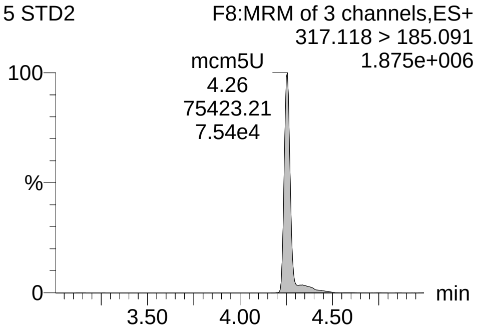

Dataset: L:\masslynx\2020DS.PRO\JH20210215R.qld

Last Altered: Monday, February 15, 2021 09:49:11 Eastern Standard Time
Printed: Monday, February 15, 2021 09:51:12 Eastern Standard Time

Name: JH20210214STD2, Date: 14-Feb-2021, Time: 14:33:18, ID: STD2, Description: 5

mcm5s2U

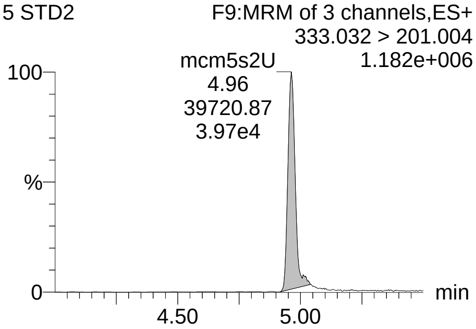

G13C15N

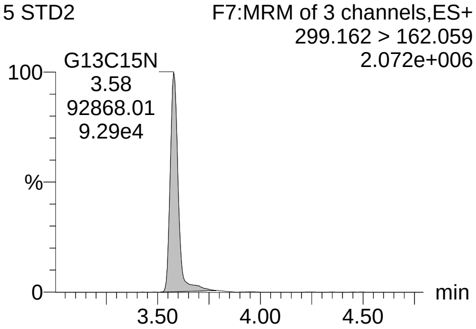

#	Name	Trace	RT	Area	IS Area	Response	Primar...	Conc.	%Dev
1	m1A	282 > 150	1.27	2580021.750	92868.008	27.782	bb	5.8	16.0
2	Gm	298.075 > 152.088	4.15	285171.375	92868.008	3.071	bb	4.8	-3.3
3	mcm5U	317.118 > 185.091	4.26	75423.211	92868.008	0.812	bb	5.1	2.9
4	mcm5s2U	333.032 > 201.004	4.96	39720.871	92868.008	0.428	bb	3.9	-22.5
5	G13C15N	299.162 > 162.059	3.58	92868.008		92868.008	bb	0.7	-31.8

Name: JH20210214STD3, Date: 14-Feb-2021, Time: 14:41:07, ID: STD3, Description: 2.5

m1A

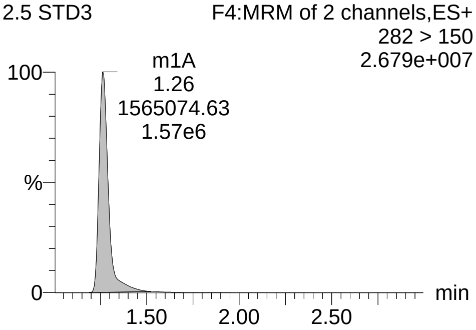

Gm

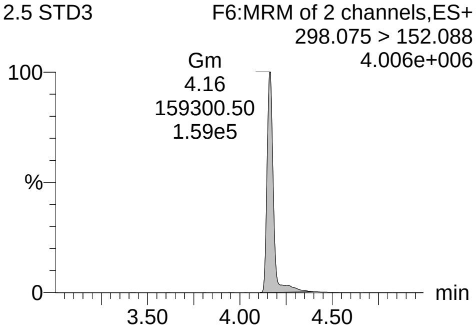

mcm5U

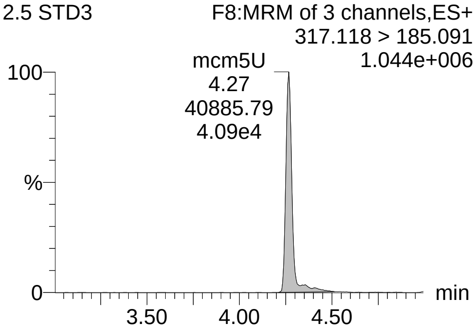

mcm5s2U

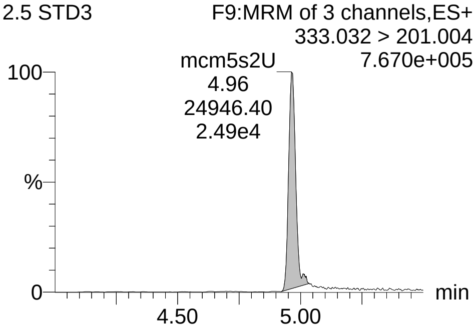

G13C15N

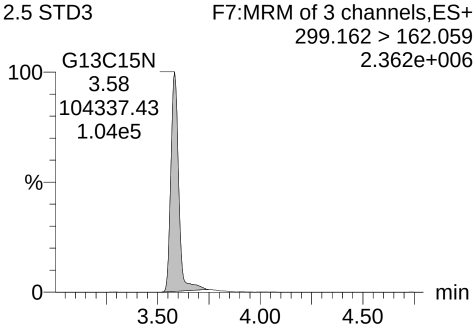

#	Name	Trace	RT	Area	IS Area	Response	Primar...	Conc.	%Dev
1	m1A	282 > 150	1.26	1565074.625	104337.430	15.000	bb	3.1	23.8
2	Gm	298.075 > 152.088	4.16	159300.500	104337.430	1.527	bb	2.4	-3.6
3	mcm5U	317.118 > 185.091	4.27	40885.785	104337.430	0.392	bb	2.5	0.6
4	mcm5s2U	333.032 > 201.004	4.96	24946.402	104337.430	0.239	bb	2.2	-12.1
5	G13C15N	299.162 > 162.059	3.58	104337.430		104337.430	bb	0.8	-23.4

Dataset: L:\masslynx\2020DS.PRO\JH20210215R.qld

Last Altered: Monday, February 15, 2021 09:49:11 Eastern Standard Time
Printed: Monday, February 15, 2021 09:51:12 Eastern Standard Time

Name: JH20210214STD4, Date: 14-Feb-2021, Time: 14:48:56, ID: STD4, Description: 1.25

m1A

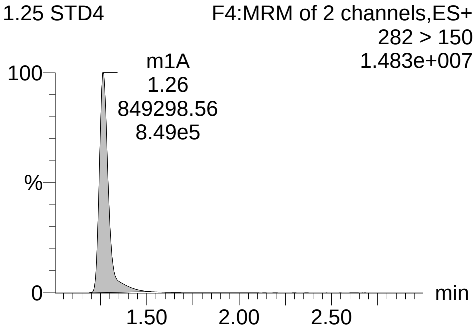

Gm

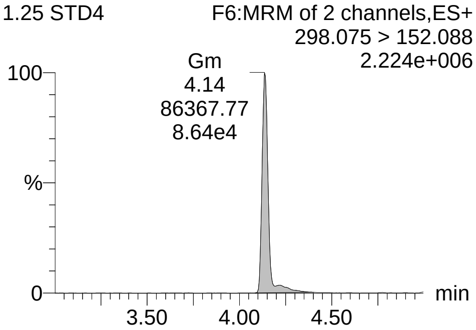

mcm5U

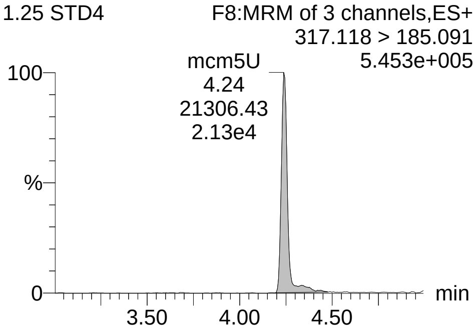

mcm5s2U

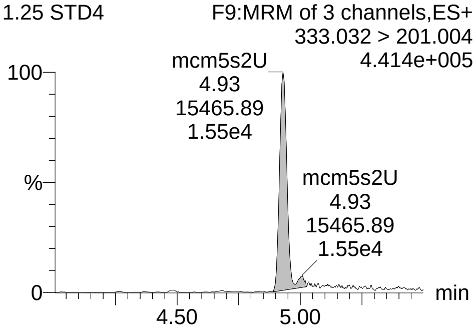

G13C15N

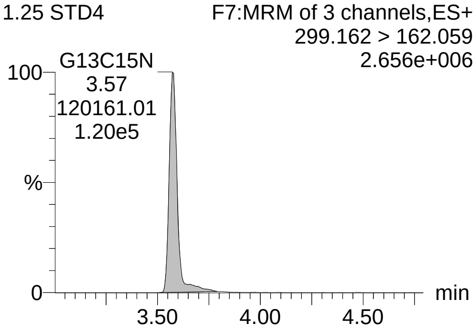

#	Name	Trace	RT	Area	IS Area	Response	Primar...	Conc.	%Dev
1	m1A	282 > 150	1.26	849298.563	120161.008	7.068	bb	1.4	13.2
2	Gm	298.075 > 152.088	4.14	86367.773	120161.008	0.719	bb	1.1	-8.7
3	mcm5U	317.118 > 185.091	4.24	21306.426	120161.008	0.177	bb	1.2	-6.2
4	mcm5s2U	333.032 > 201.004	4.93	15465.890	120161.008	0.129	bb	1.2	-2.7
5	G13C15N	299.162 > 162.059	3.57	120161.008		120161.008	bb	0.9	-11.8

Name: JH20210214STD5, Date: 14-Feb-2021, Time: 14:56:45, ID: STD5, Description: 0.625

m1A

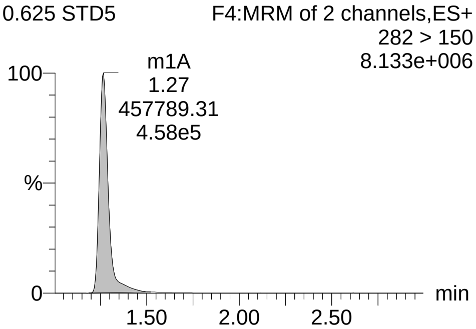

Gm

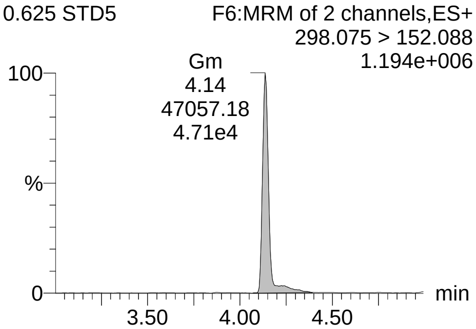

mcm5U

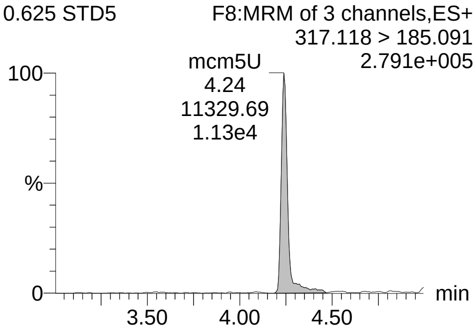

Dataset: L:\masslynx\2020DS.PRO\JH20210215R.qld

Last Altered: Monday, February 15, 2021 09:49:11 Eastern Standard Time
Printed: Monday, February 15, 2021 09:51:12 Eastern Standard Time

Name: JH20210214STD5, Date: 14-Feb-2021, Time: 14:56:45, ID: STD5, Description: 0.625

mcm5s2U

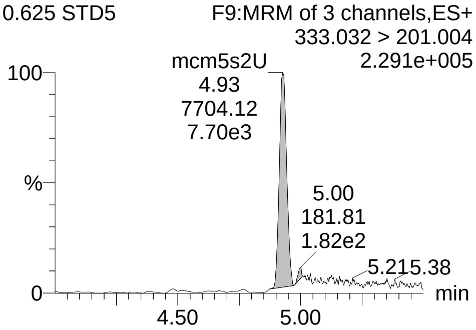

G13C15N

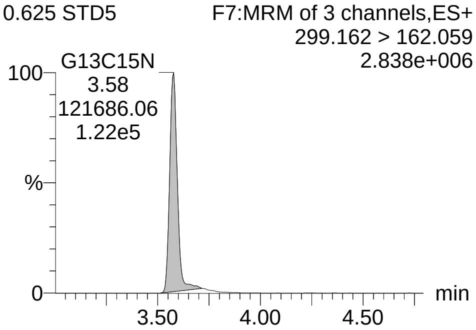

#	Name	Trace	RT	Area	IS Area	Response	Primar...	Conc.	%Dev
1	m1A	282 > 150	1.27	457789.313	121686.063	3.762	bb	0.7	14.4
2	Gm	298.075 > 152.088	4.14	47057.184	121686.063	0.387	bb	0.6	-0.7
3	mcm5U	317.118 > 185.091	4.24	11329.688	121686.063	0.093	bb	0.6	3.3
4	mcm5s2U	333.032 > 201.004	4.93	7704.117	121686.063	0.063	bb	0.6	1.6
5	G13C15N	299.162 > 162.059	3.58	121686.063		121686.063	bb	0.9	-10.6

Name: JH20210214STD6, Date: 14-Feb-2021, Time: 15:04:33, ID: STD6, Description: 0.3125

m1A

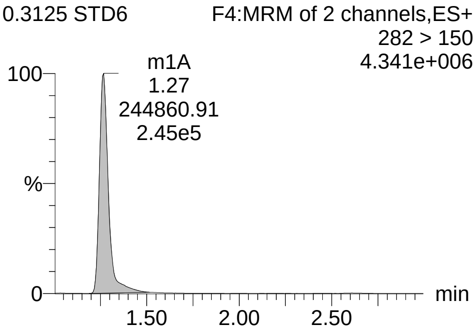

Gm

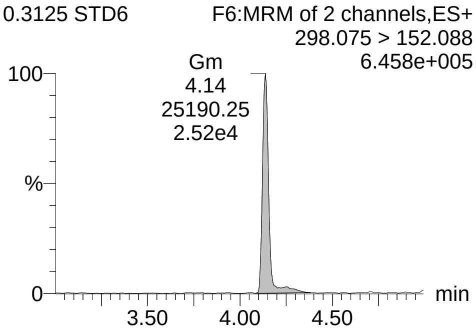

mcm5U

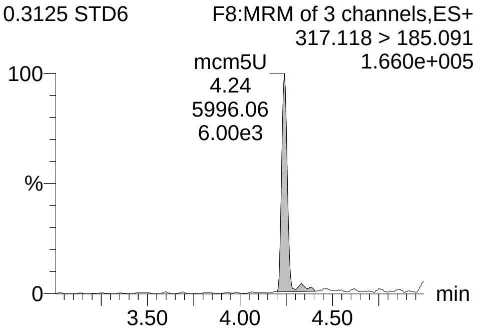

mcm5s2U

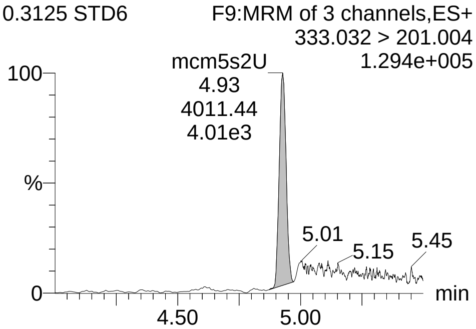

G13C15N

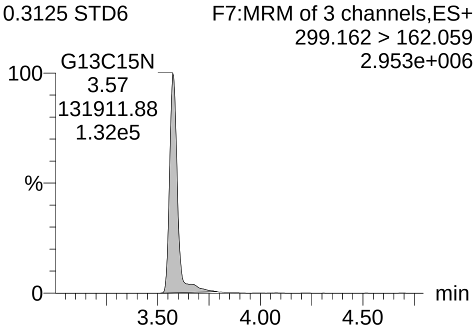

#	Name	Trace	RT	Area	IS Area	Response	Primar...	Conc.	%Dev
1	m1A	282 > 150	1.27	244860.906	131911.875	1.856	bb	0.3	-0.3
2	Gm	298.075 > 152.088	4.14	25190.252	131911.875	0.191	bb	0.3	0.2
3	mcm5U	317.118 > 185.091	4.24	5996.057	131911.875	0.045	bb	0.3	11.2
4	mcm5s2U	333.032 > 201.004	4.93	4011.439	131911.875	0.030	bb	0.3	9.6
5	G13C15N	299.162 > 162.059	3.57	131911.875		131911.875	bb	1.0	-3.1

Dataset: L:\masslynx\2020DS.PRO\JH20210215R.qld

Last Altered: Monday, February 15, 2021 09:49:11 Eastern Standard Time
Printed: Monday, February 15, 2021 09:51:12 Eastern Standard Time

Name: JH20210214STD7, Date: 14-Feb-2021, Time: 15:12:22, ID: STD7, Description: 0.15625

m1A

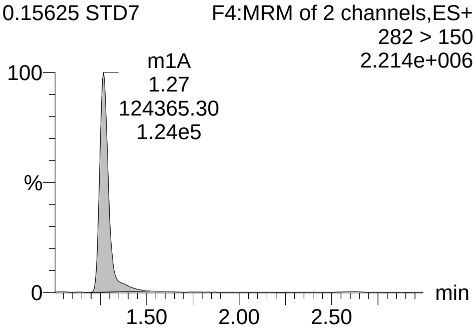

Gm

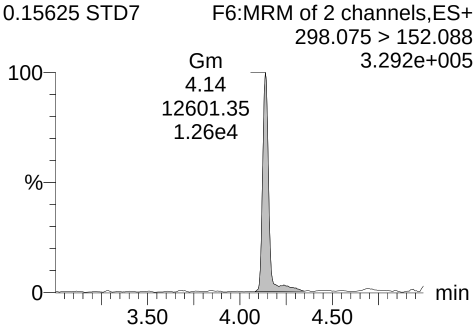

mcm5U

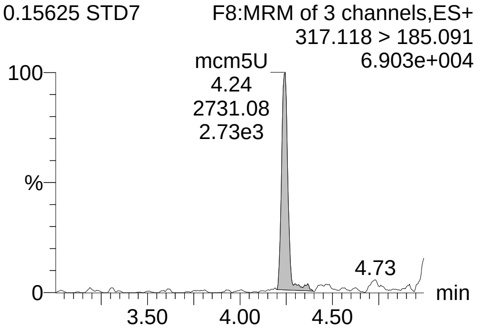

mcm5s2U

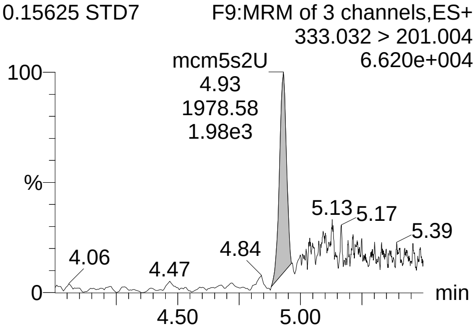

G13C15N

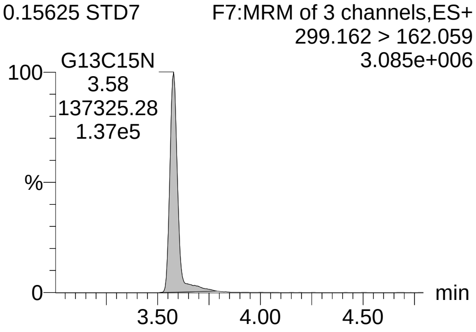

#	Name	Trace	RT	Area	IS Area	Response	Primar...	Conc.	%Dev
1	m1A	282 > 150	1.27	124365.297	137325.281	0.906	bb	0.1	-29.4
2	Gm	298.075 > 152.088	4.14	12601.346	137325.281	0.092	bb	0.2	0.8
3	mcm5U	317.118 > 185.091	4.24	2731.084	137325.281	0.020	bb	0.2	20.0
4	mcm5s2U	333.032 > 201.004	4.93	1978.575	137325.281	0.014	bb	0.2	28.1
5	G13C15N	299.162 > 162.059	3.58	137325.281		137325.281	bb	1.0	0.8

Name: JH20210214STD8, Date: 14-Feb-2021, Time: 15:20:10, ID: STD8, Description: 0.078125

m1A

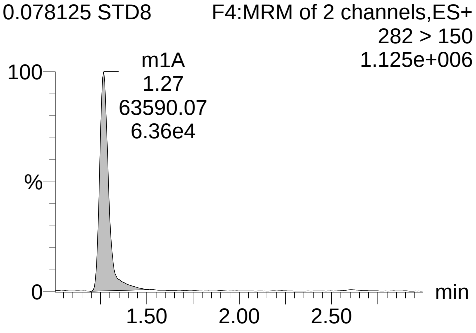

Gm

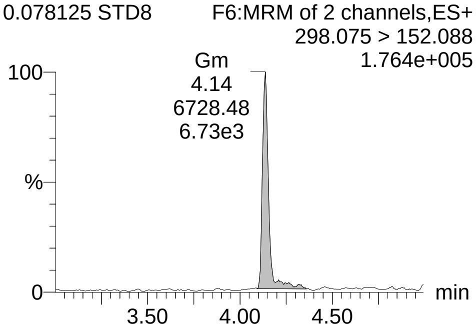

mcm5U

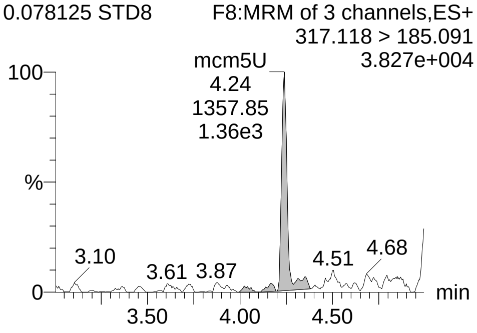

Dataset: L:\masslynx\2020DS.PRO\JH20210215R.qld

Last Altered: Monday, February 15, 2021 09:49:11 Eastern Standard Time
Printed: Monday, February 15, 2021 09:51:12 Eastern Standard Time

Name: JH20210214STD8, Date: 14-Feb-2021, Time: 15:20:10, ID: STD8, Description: 0.078125

mcm5s2U

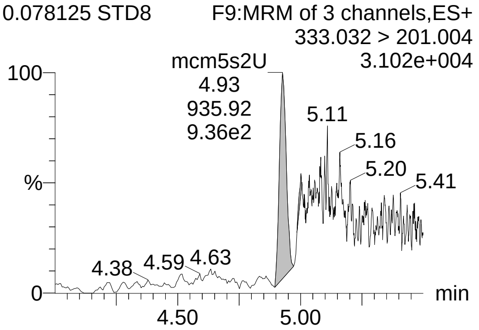

G13C15N

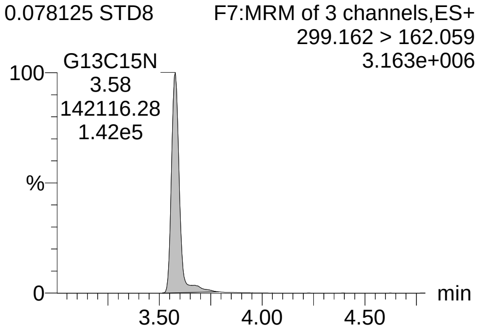

#	Name	Trace	RT	Area	IS Area	Response	Primar...	Conc.	%Dev
1	m1A	282 > 150	1.27	63590.074	142116.281	0.447	bbX	0.0	-83.0
2	Gm	298.075 > 152.088	4.14	6728.483	142116.281	0.047	bb	0.1	12.3
3	mcm5U	317.118 > 185.091	4.24	1357.850	142116.281	0.010	bbX	0.1	57.3
4	mcm5s2U	333.032 > 201.004	4.93	935.918	142116.281	0.007	bbX	0.1	67.1
5	G13C15N	299.162 > 162.059	3.58	142116.281		142116.281	bb	1.0	4.4

Name: JH20210214Blank1, Date: 14-Feb-2021, Time: 15:27:59, ID: blk, Description: blank

m1A

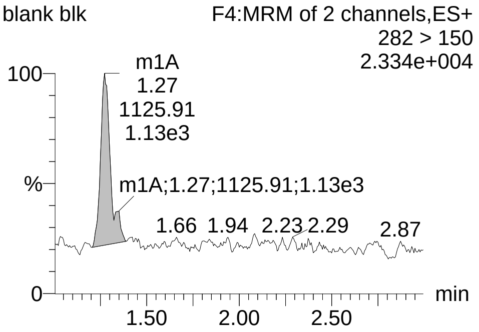

Gm

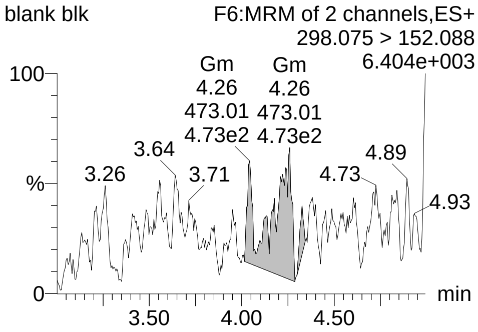

mcm5U

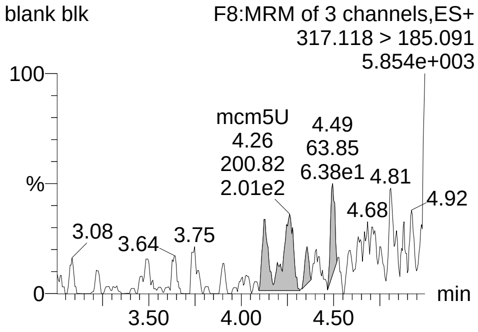

mcm5s2U

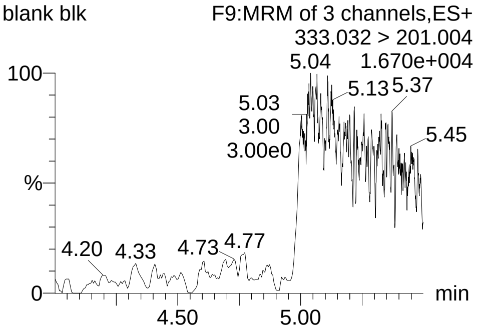

G13C15N

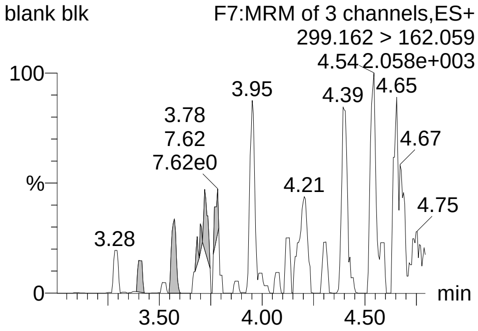

#	Name	Trace	RT	Area	IS Area	Response	Primar...	Conc.	%Dev
1	m1A	282 > 150	1.27	1125.910	18.381	61.254	bb	12.9	
2	Gm	298.075 > 152.088	4.26	473.015	18.381	25.734	bb	40.4	
3	mcm5U	317.118 > 185.091	4.26	200.816	18.381	10.925	bb	68.4	
4	mcm5s2U	333.032 > 201.004			18.381				
5	G13C15N	299.162 > 162.059	3.57	18.381		18.381	bb	0.0	-100.0

Dataset: L:\masslynx\2020DS.PRO\JH20210215R.qld

Last Altered: Monday, February 15, 2021 09:49:11 Eastern Standard Time
Printed: Monday, February 15, 2021 09:51:12 Eastern Standard Time

Name: JH20210214S1_1, Date: 14-Feb-2021, Time: 15:35:48, ID: S1, Description: A+IS

m1A

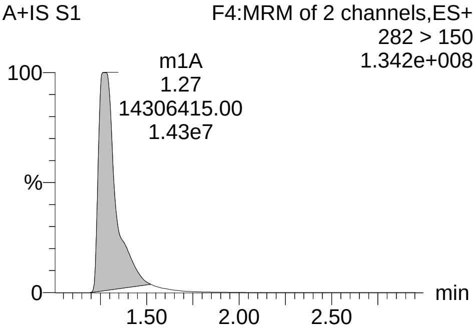

Gm

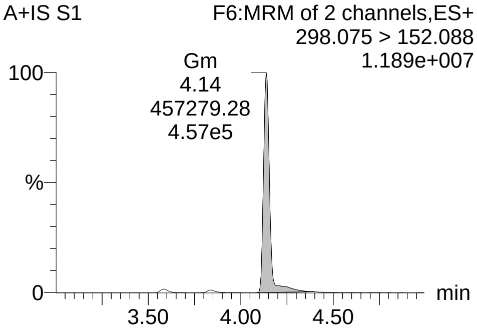

mcm5U

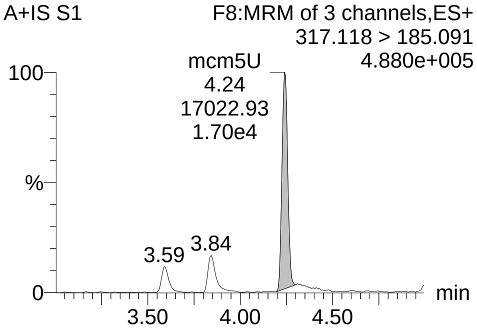

mcm5s2U

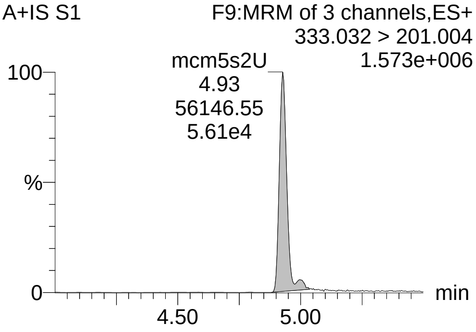

G13C15N

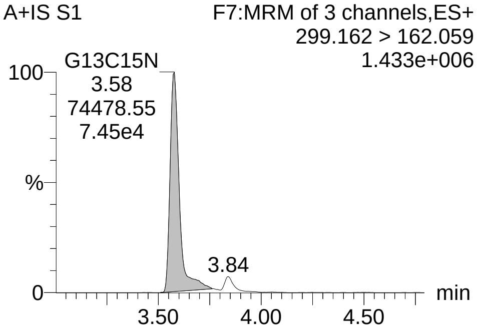

#	Name	Trace	RT	Area	IS Area	Response	Primar...	Conc.	%Dev
1	m1A	282 > 150	1.27	14306415.0...	74478.555	192.088	bb	40.6	
2	Gm	298.075 > 152.088	4.14	457279.281	74478.555	6.140	bb	9.7	
3	mcm5U	317.118 > 185.091	4.24	17022.926	74478.555	0.229	bb	1.5	
4	mcm5s2U	333.032 > 201.004	4.93	56146.551	74478.555	0.754	bb	6.8	
5	G13C15N	299.162 > 162.059	3.58	74478.555		74478.555	bb	0.5	-45.3

Name: JH20210214S1_2, Date: 14-Feb-2021, Time: 15:43:37, ID: S2, Description: A+IS

m1A

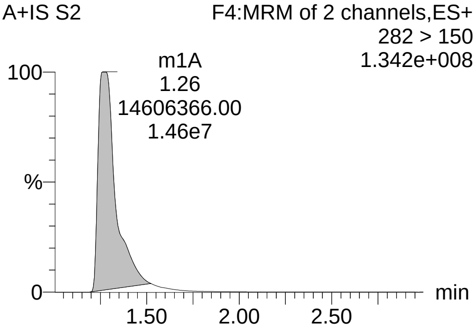

Gm

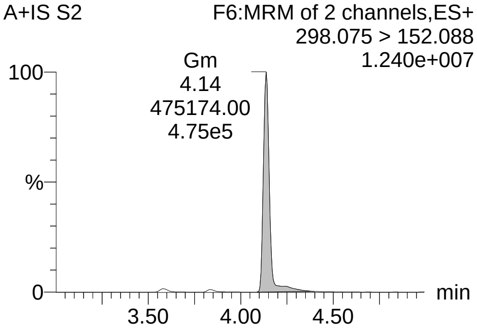

mcm5U

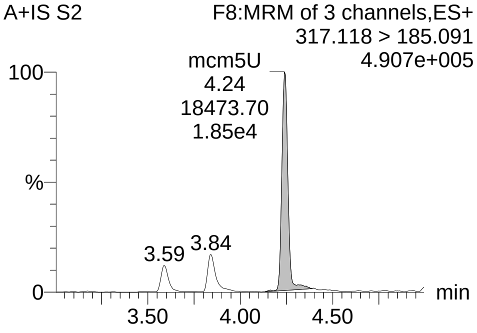

Dataset: L:\masslynx\2020DS.PRO\JH20210215R.qld

Last Altered: Monday, February 15, 2021 09:49:11 Eastern Standard Time
Printed: Monday, February 15, 2021 09:51:12 Eastern Standard Time

Name: JH20210214S1_2, Date: 14-Feb-2021, Time: 15:43:37, ID: S2, Description: A+IS

mcm5s2U

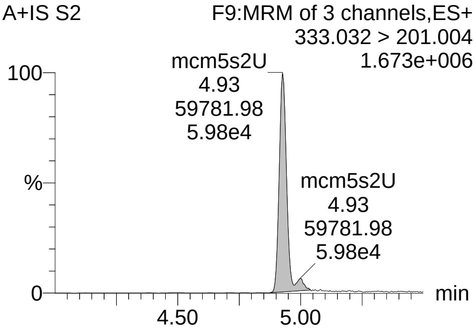

G13C15N

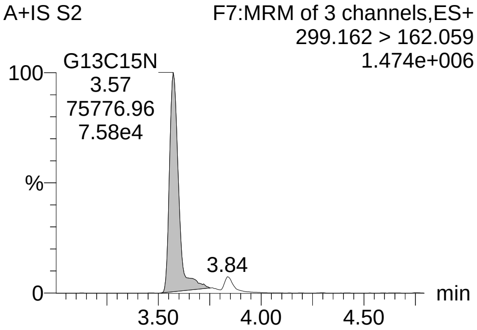

#	Name	Trace	RT	Area	IS Area	Response	Primar...	Conc.	%Dev
1	m1A	282 > 150	1.26	14606366.0...	75776.961	192.755	bb	40.7	
2	Gm	298.075 > 152.088	4.14	475174.000	75776.961	6.271	bb	9.9	
3	mcm5U	317.118 > 185.091	4.24	18473.699	75776.961	0.244	bb	1.6	
4	mcm5s2U	333.032 > 201.004	4.93	59781.984	75776.961	0.789	bb	7.1	
5	G13C15N	299.162 > 162.059	3.57	75776.961		75776.961	bb	0.6	-44.4

Name: JH20210214S2_1, Date: 14-Feb-2021, Time: 15:51:25, ID: S3, Description: B+IS

m1A

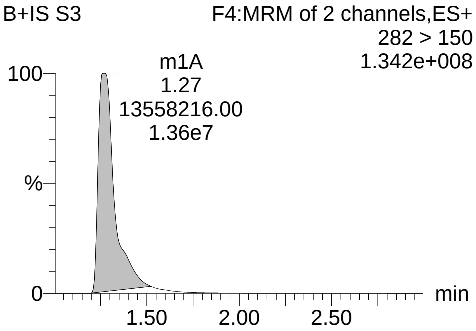

Gm

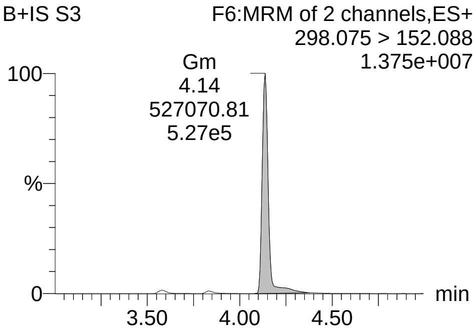

mcm5U

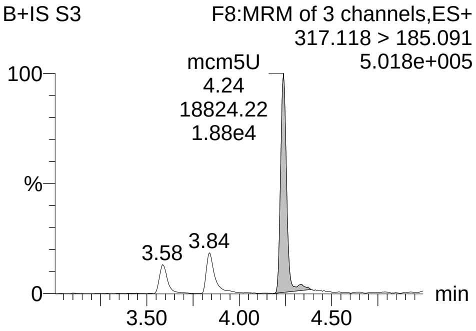

mcm5s2U

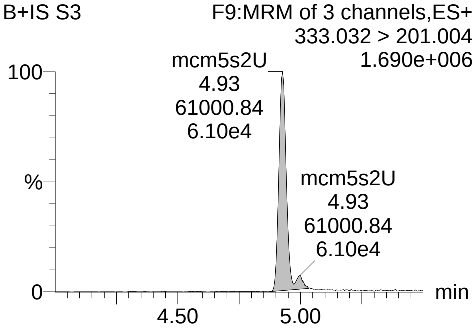

G13C15N

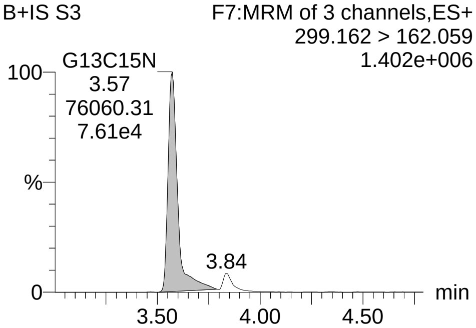

#	Name	Trace	RT	Area	IS Area	Response	Primar...	Conc.	%Dev
1	m1A	282 > 150	1.27	13558216.0...	76060.313	178.256	bb	37.7	
2	Gm	298.075 > 152.088	4.14	527070.813	76060.313	6.930	bb	10.9	
3	mcm5U	317.118 > 185.091	4.24	18824.215	76060.313	0.247	bb	1.6	
4	mcm5s2U	333.032 > 201.004	4.93	61000.836	76060.313	0.802	bb	7.2	
5	G13C15N	299.162 > 162.059	3.57	76060.313		76060.313	bb	0.6	-44.1

Dataset: L:\masslynx\2020DS.PRO\JH20210215R.qld

Last Altered: Monday, February 15, 2021 09:49:11 Eastern Standard Time
Printed: Monday, February 15, 2021 09:51:12 Eastern Standard Time

Name: JH20210214S2_2, Date: 14-Feb-2021, Time: 15:59:14, ID: S4, Description: B+IS

m1A

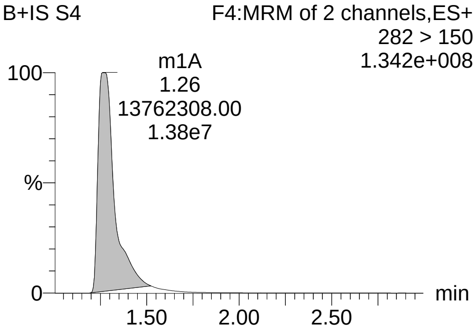

Gm

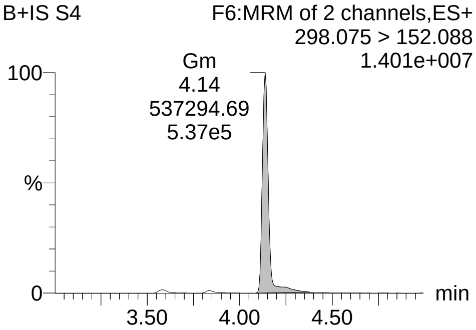

mcm5U

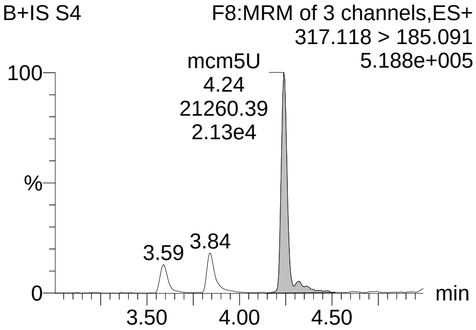

mcm5s2U

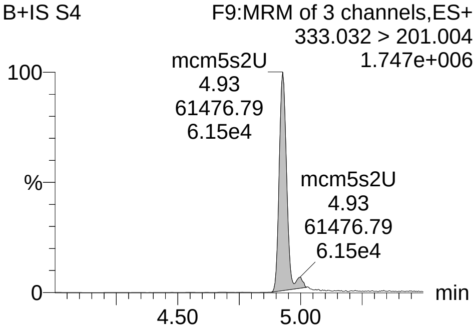

G13C15N

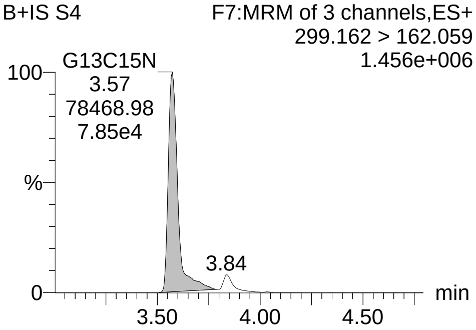

#	Name	Trace	RT	Area	IS Area	Response	Primar...	Conc.	%Dev
1	m1A	282 > 150	1.26	13762308.0...	78468.984	175.385	bb	37.0	
2	Gm	298.075 > 152.088	4.14	537294.688	78468.984	6.847	bb	10.8	
3	mcm5U	317.118 > 185.091	4.24	21260.393	78468.984	0.271	bb	1.8	
4	mcm5s2U	333.032 > 201.004	4.93	61476.793	78468.984	0.783	bb	7.0	
5	G13C15N	299.162 > 162.059	3.57	78468.984		78468.984	bb	0.6	-42.4

Name: JH20210214S3_1, Date: 14-Feb-2021, Time: 16:07:03, ID: S5, Description: C+IS

m1A

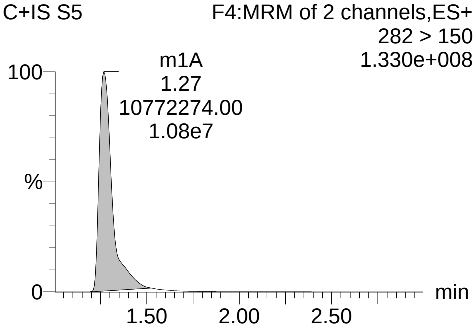

Gm

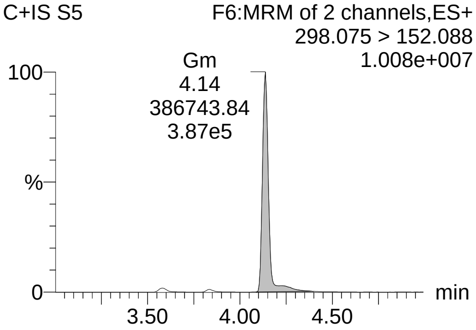

mcm5U

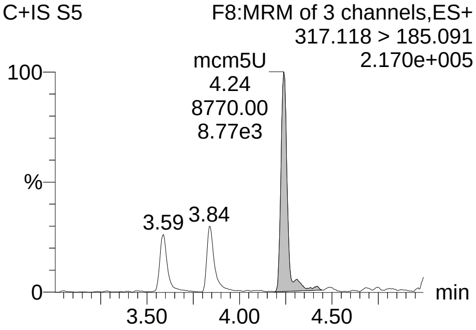

Dataset: L:\masslynx\2020DS.PRO\JH20210215R.qld

Last Altered: Monday, February 15, 2021 09:49:11 Eastern Standard Time
Printed: Monday, February 15, 2021 09:51:12 Eastern Standard Time

Name: JH20210214S3_1, Date: 14-Feb-2021, Time: 16:07:03, ID: S5, Description: C+IS

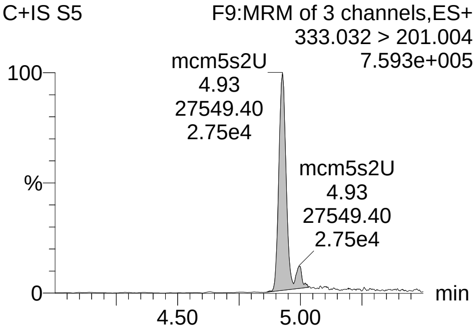

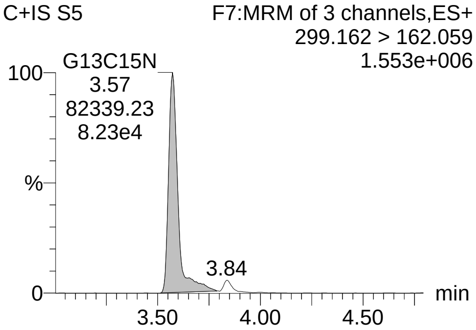

#	Name	Trace	RT	Area	IS Area	Response	Primar...	Conc.	%Dev
1	m1A	282 > 150	1.27	10772274.0...	82339.227	130.828	bb	27.6	
2	Gm	298.075 > 152.088	4.14	386743.844	82339.227	4.697	bb	7.4	
3	mcm5U	317.118 > 185.091	4.24	8769.999	82339.227	0.107	bb	0.7	
4	mcm5s2U	333.032 > 201.004	4.93	27549.400	82339.227	0.335	bb	3.0	
5	G13C15N	299.162 > 162.059	3.57	82339.227		82339.227	bb	0.6	-39.5

Name: JH20210214S3_2, Date: 14-Feb-2021, Time: 16:14:51, ID: S6, Description: C+IS

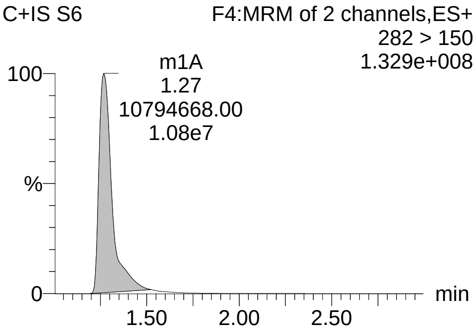

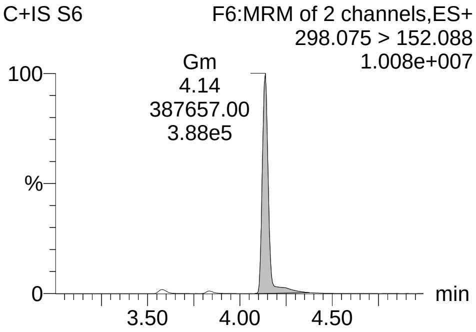

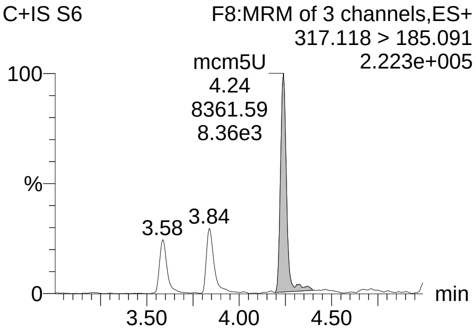

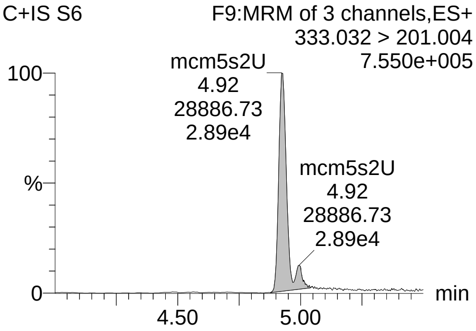

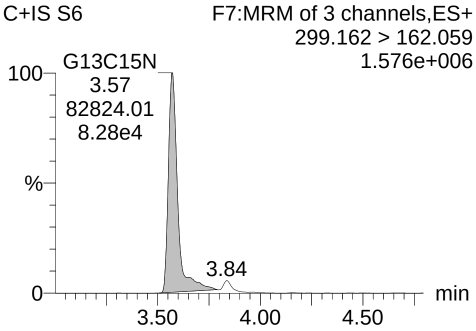

#	Name	Trace	RT	Area	IS Area	Response	Primar...	Conc.	%Dev
1	m1A	282 > 150	1.27	10794668.0...	82824.008	130.333	bb	27.5	
2	Gm	298.075 > 152.088	4.14	387657.000	82824.008	4.680	bb	7.4	
3	mcm5U	317.118 > 185.091	4.24	8361.593	82824.008	0.101	bb	0.7	
4	mcm5s2U	333.032 > 201.004	4.92	28886.730	82824.008	0.349	bb	3.2	
5	G13C15N	299.162 > 162.059	3.57	82824.008		82824.008	bb	0.6	-39.2

Dataset: L:\masslynx\2020DS.PRO\JH20210215R.qld

Last Altered: Monday, February 15, 2021 09:49:11 Eastern Standard Time
Printed: Monday, February 15, 2021 09:51:12 Eastern Standard Time

Name: JH20210214S4_1, Date: 14-Feb-2021, Time: 16:22:40, ID: S7, Description: D+IS

m1A

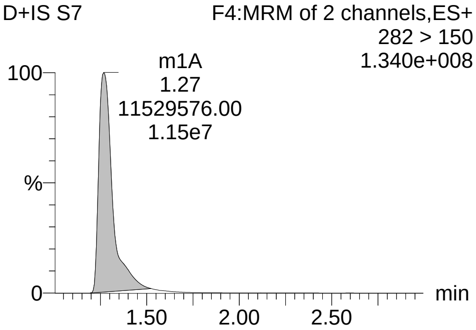

Gm

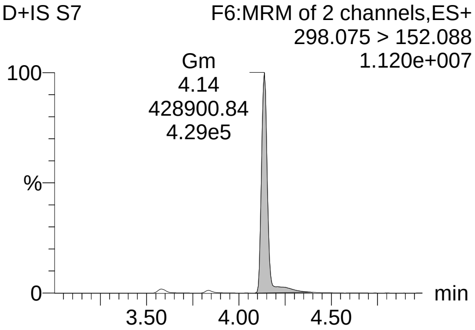

mcm5U

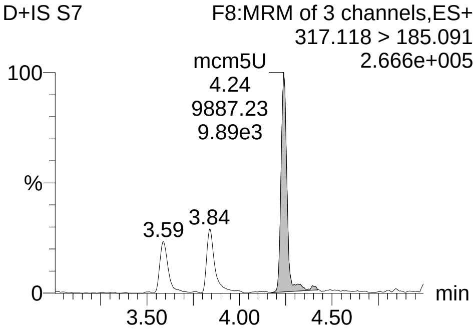

mcm5s2U

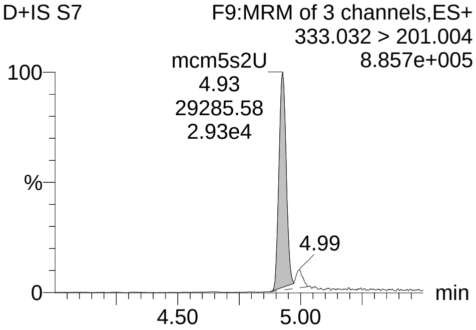

G13C15N

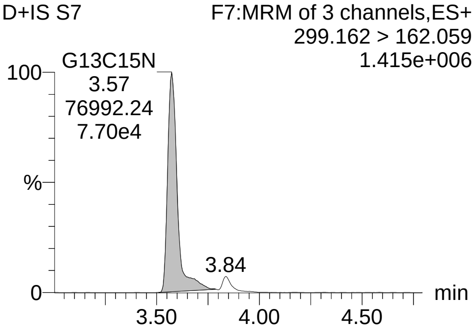

#	Name	Trace	RT	Area	IS Area	Response	Primar...	Conc.	%Dev
1	m1A	282 > 150	1.27	11529576.0...	76992.242	149.750	bb	31.6	
2	Gm	298.075 > 152.088	4.14	428900.844	76992.242	5.571	bb	8.8	
3	mcm5U	317.118 > 185.091	4.24	9887.230	76992.242	0.128	bb	0.9	
4	mcm5s2U	333.032 > 201.004	4.93	29285.582	76992.242	0.380	MM	3.5	
5	G13C15N	299.162 > 162.059	3.57	76992.242		76992.242	bb	0.6	-43.5

Name: JH20210214S4_2, Date: 14-Feb-2021, Time: 16:30:29, ID: S8, Description: D+IS

m1A

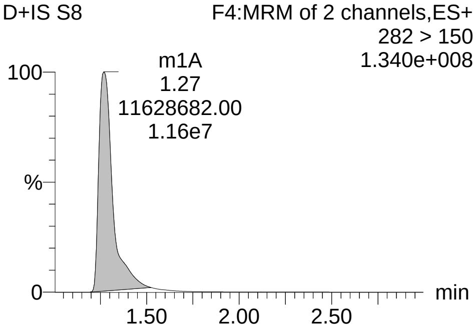

Gm

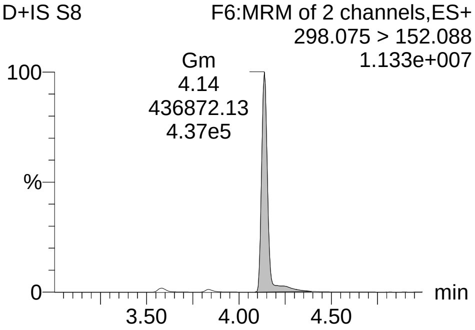

mcm5U

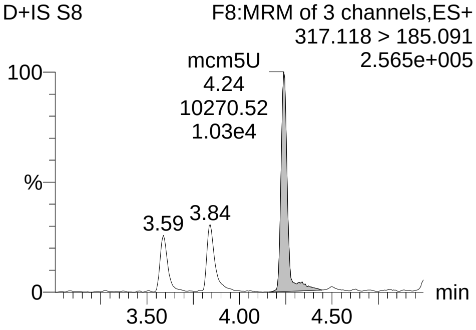

Dataset: L:\masslynx\2020DS.PRO\JH20210215R.qld

Last Altered: Monday, February 15, 2021 09:49:11 Eastern Standard Time
Printed: Monday, February 15, 2021 09:51:12 Eastern Standard Time

Name: JH20210214S4_2, Date: 14-Feb-2021, Time: 16:30:29, ID: S8, Description: D+IS

mcm5s2U

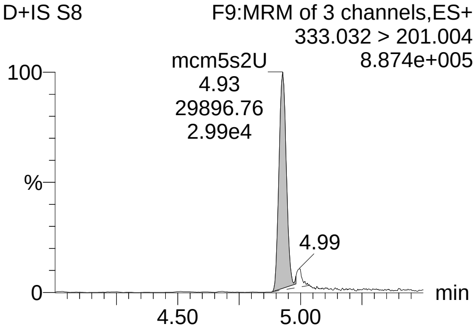

G13C15N

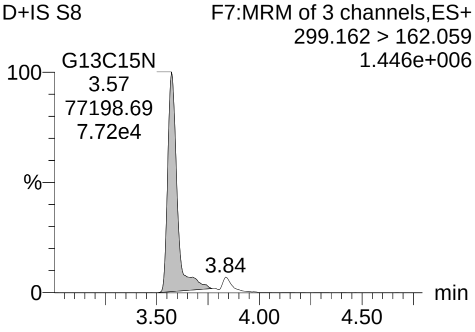

#	Name	Trace	RT	Area	IS Area	Response	Primar...	Conc.	%Dev
1	m1A	282 > 150	1.27	11628682.0...	77198.688	150.633	bb	31.8	
2	Gm	298.075 > 152.088	4.14	436872.125	77198.688	5.659	bb	8.9	
3	mcm5U	317.118 > 185.091	4.24	10270.523	77198.688	0.133	bb	0.9	
4	mcm5s2U	333.032 > 201.004	4.93	29896.760	77198.688	0.387	MM	3.5	
5	G13C15N	299.162 > 162.059	3.57	77198.688		77198.688	bb	0.6	-43.3

Name: JH20210214blank2, Date: 14-Feb-2021, Time: 16:38:17, ID: blk, Description: blank

m1A

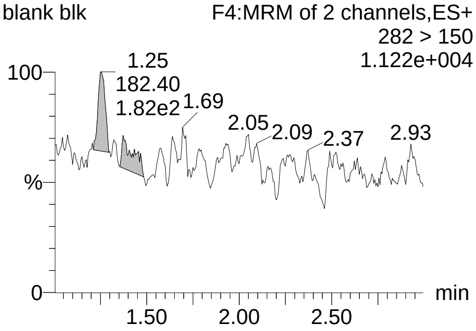

Gm

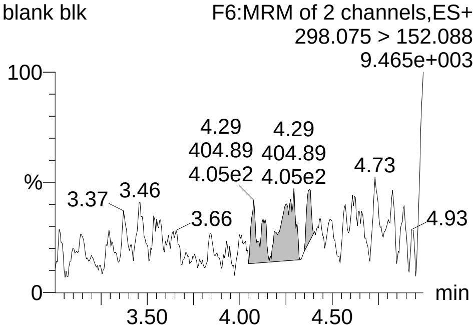

mcm5U

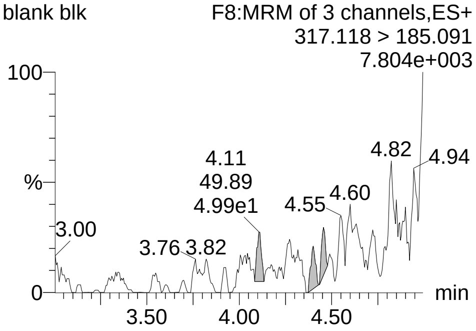

mcm5s2U

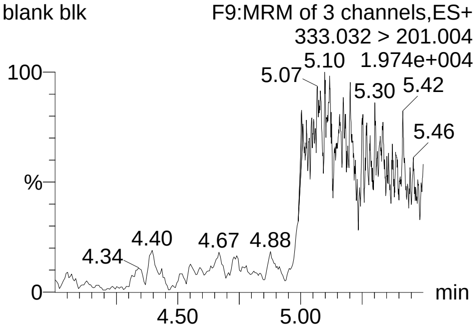

G13C15N

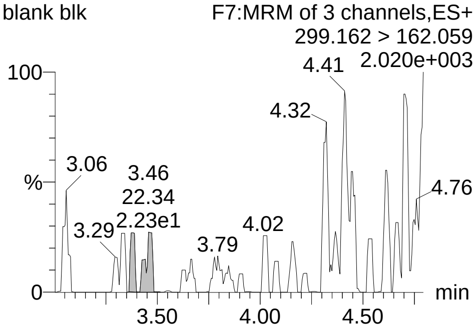

#	Name	Trace	RT	Area	IS Area	Response	Primar...	Conc.	%Dev
1	m1A	282 > 150	1.37	120.237			bb		
2	Gm	298.075 > 152.088							
3	mcm5U	317.118 > 185.091							
4	mcm5s2U	333.032 > 201.004							
5	G13C15N	299.162 > 162.059							

Dataset: L:\masslynx\2020DS.PRO\JH20210215R.qld

Last Altered: Monday, February 15, 2021 09:49:11 Eastern Standard Time
Printed: Monday, February 15, 2021 09:51:12 Eastern Standard Time

Name: JH20210214SSTD1, Date: 14-Feb-2021, Time: 16:46:06, ID: STD1, Description: 10 pg/ul+ISGm/m1A/mcm5U/mcm5s2U

m1A

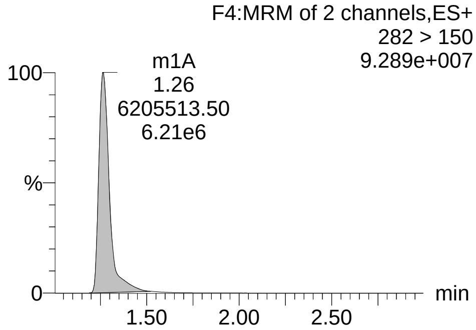

Gm

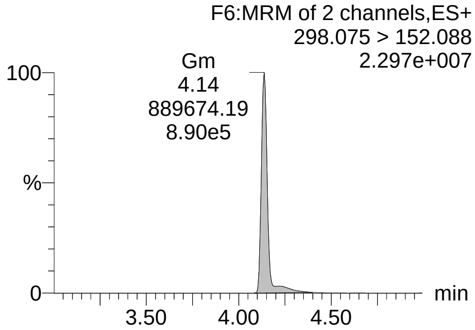

mcm5U

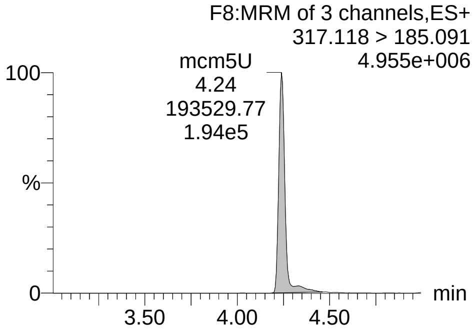

mcm5s2U

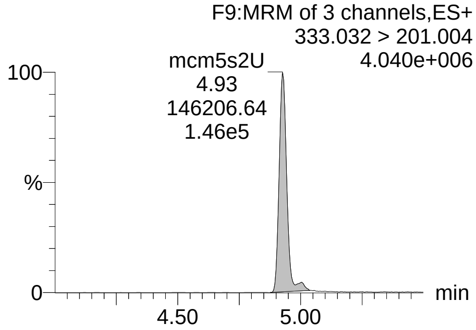

G13C15N

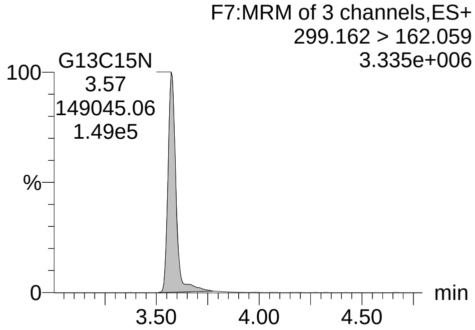

#	Name	Trace	RT	Area	IS Area	Response	Primar...	Conc.	%Dev
1	m1A	282 > 150	1.26	6205513.500	149045.063	41.635	bb	8.7	-12.7
2	Gm	298.075 > 152.088	4.14	889674.188	149045.063	5.969	bb	9.4	-6.2
3	mcm5U	317.118 > 185.091	4.24	193529.766	149045.063	1.298	bb	8.2	-18.2
4	mcm5s2U	333.032 > 201.004	4.93	146206.641	149045.063	0.981	bb	8.8	-12.1
5	G13C15N	299.162 > 162.059	3.57	149045.063		149045.063	bb	1.1	9.5

Name: JH20210214SSTD2, Date: 14-Feb-2021, Time: 16:53:55, ID: STD2, Description: 5

m1A

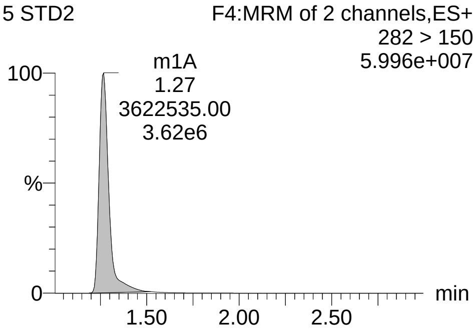

Gm

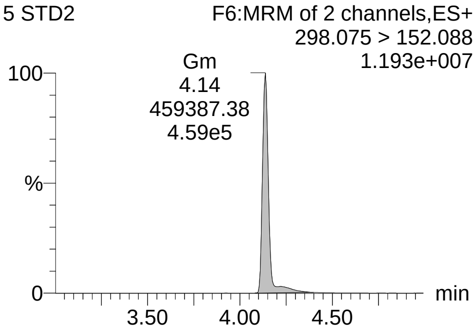

mcm5U

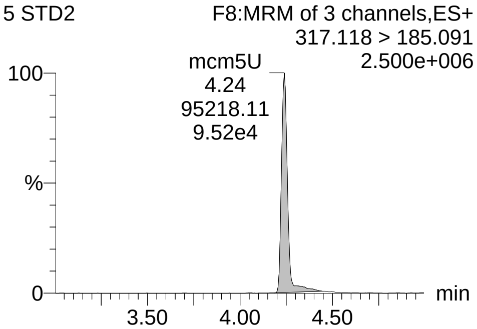

Dataset: L:\masslynx\2020DS.PRO\JH20210215R.qld

Last Altered: Monday, February 15, 2021 09:49:11 Eastern Standard Time
Printed: Monday, February 15, 2021 09:51:12 Eastern Standard Time

Name: JH20210214SSTD2, Date: 14-Feb-2021, Time: 16:53:55, ID: STD2, Description: 5

mcm5s2U

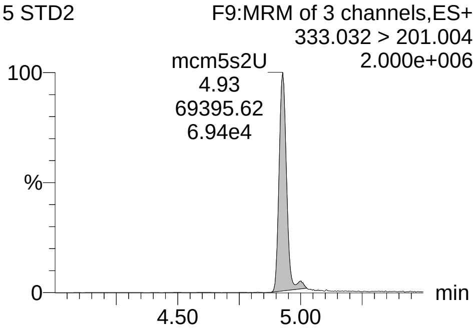

G13C15N

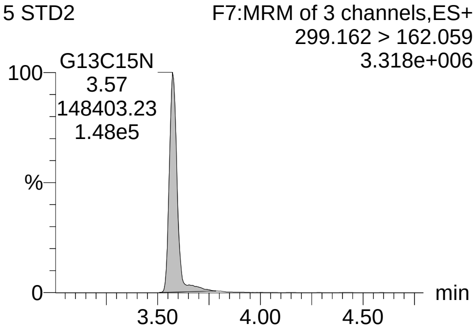

#	Name	Trace	RT	Area	IS Area	Response	Primar...	Conc.	%Dev
1	m1A	282 > 150	1.27	3622535.000	148403.234	24.410	bb	5.1	1.7
2	Gm	298.075 > 152.088	4.14	459387.375	148403.234	3.096	bb	4.9	-2.6
3	mcm5U	317.118 > 185.091	4.24	95218.109	148403.234	0.642	bb	4.1	-18.5
4	mcm5s2U	333.032 > 201.004	4.93	69395.617	148403.234	0.468	bb	4.2	-15.4
5	G13C15N	299.162 > 162.059	3.57	148403.234		148403.234	bb	1.1	9.0

Name: JH20210214SSTD3, Date: 14-Feb-2021, Time: 17:01:43, ID: STD3, Description: 2.5

m1A

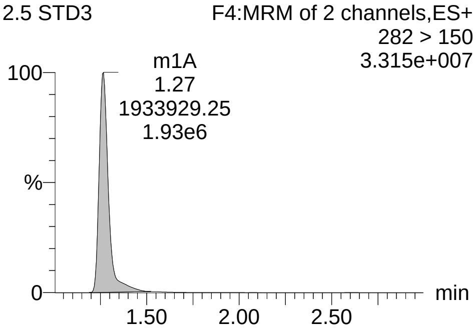

Gm

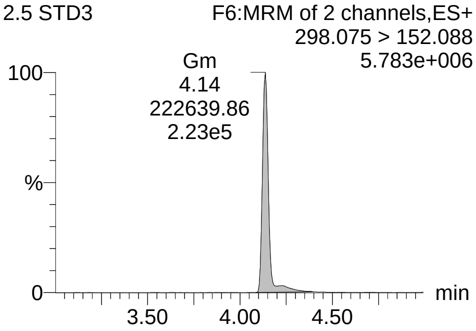

mcm5U

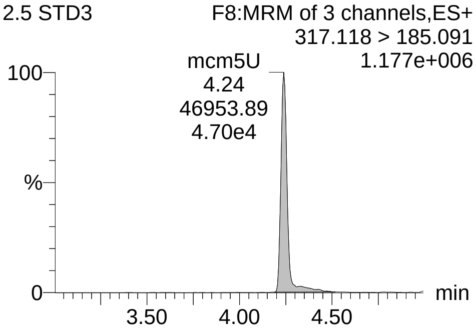

mcm5s2U

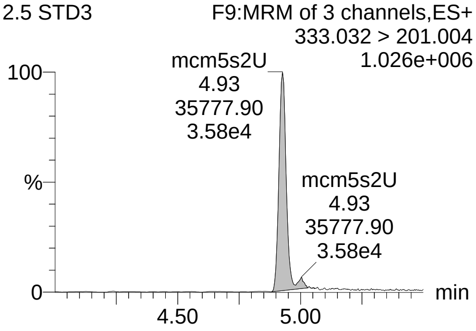

G13C15N

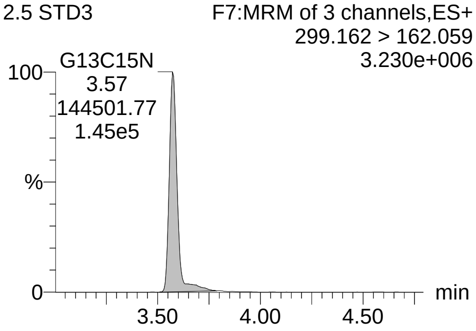

#	Name	Trace	RT	Area	IS Area	Response	Primar...	Conc.	%Dev
1	m1A	282 > 150	1.27	1933929.250	144501.766	13.383	bb	2.8	10.1
2	Gm	298.075 > 152.088	4.14	222639.859	144501.766	1.541	bb	2.4	-2.7
3	mcm5U	317.118 > 185.091	4.24	46953.887	144501.766	0.325	bb	2.1	-16.2
4	mcm5s2U	333.032 > 201.004	4.93	35777.902	144501.766	0.248	bb	2.3	-9.1
5	G13C15N	299.162 > 162.059	3.57	144501.766		144501.766	bb	1.1	6.1

Dataset: L:\masslynx\2020DS.PRO\JH20210215R.qld

Last Altered: Monday, February 15, 2021 09:49:11 Eastern Standard Time
Printed: Monday, February 15, 2021 09:51:12 Eastern Standard Time

Name: JH20210214SSTD4, Date: 14-Feb-2021, Time: 17:09:32, ID: STD4, Description: 1.25

m1A

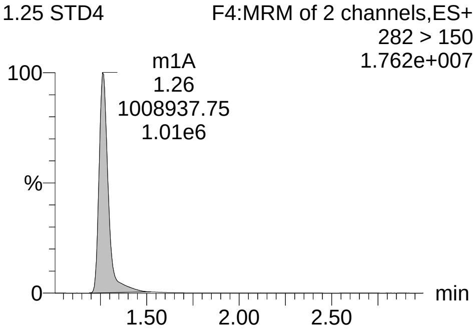

Gm

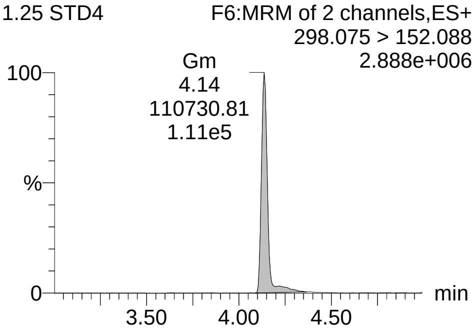

mcm5U

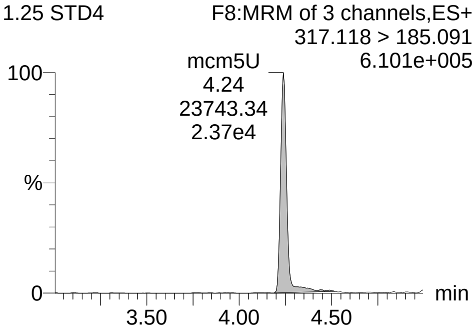

mcm5s2U

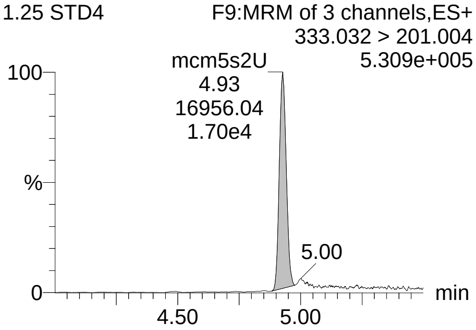

G13C15N

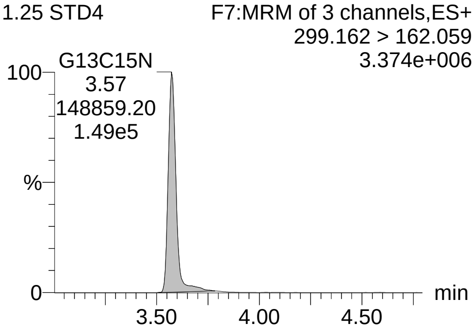

#	Name	Trace	RT	Area	IS Area	Response	Primar...	Conc.	%Dev
1	m1A	282 > 150	1.26	1008937.750	148859.203	6.778	bb	1.4	8.3
2	Gm	298.075 > 152.088	4.14	110730.813	148859.203	0.744	bb	1.2	-5.5
3	mcm5U	317.118 > 185.091	4.24	23743.340	148859.203	0.160	bb	1.1	-15.1
4	mcm5s2U	333.032 > 201.004	4.93	16956.041	148859.203	0.114	bb	1.1	-13.2
5	G13C15N	299.162 > 162.059	3.57	148859.203		148859.203	bb	1.1	9.3

Name: JH20210214SSTD5, Date: 14-Feb-2021, Time: 17:17:21, ID: STD5, Description: 0.625

m1A

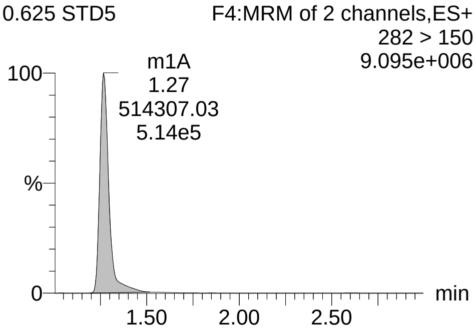

Gm

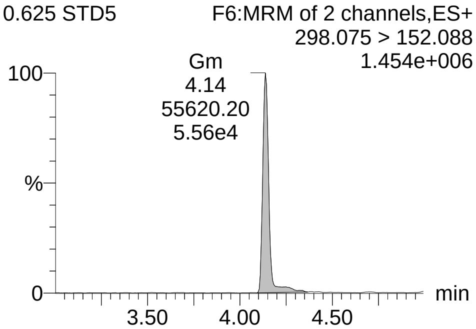

mcm5U

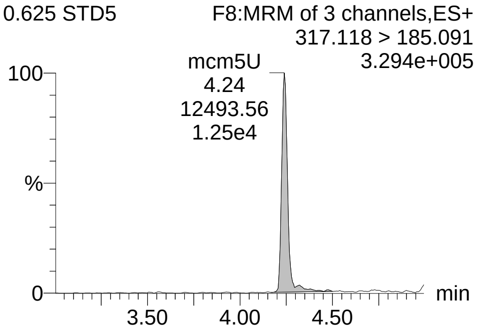

Dataset: L:\masslynx\2020DS.PRO\JH20210215R.qld

Last Altered: Monday, February 15, 2021 09:49:11 Eastern Standard Time
Printed: Monday, February 15, 2021 09:51:12 Eastern Standard Time

Name: JH20210214SSTD5, Date: 14-Feb-2021, Time: 17:17:21, ID: STD5, Description: 0.625

mcm5s2U

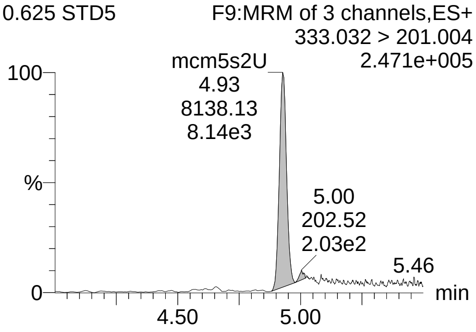

G13C15N

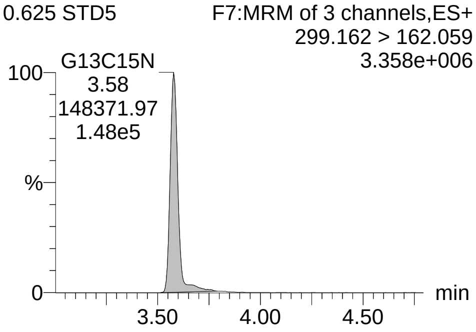

#	Name	Trace	RT	Area	IS Area	Response	Primar...	Conc.	%Dev
1	m1A	282 > 150	1.27	514307.031	148371.969	3.466	bb	0.7	4.4
2	Gm	298.075 > 152.088	4.14	55620.199	148371.969	0.375	bb	0.6	-3.7
3	mcm5U	317.118 > 185.091	4.24	12493.555	148371.969	0.084	bb	0.6	-5.6
4	mcm5s2U	333.032 > 201.004	4.93	8138.126	148371.969	0.055	bb	0.6	-10.5
5	G13C15N	299.162 > 162.059	3.58	148371.969		148371.969	bb	1.1	9.0

Name: JH20210214SSTD6, Date: 14-Feb-2021, Time: 17:25:09, ID: STD6, Description: 0.3125

m1A

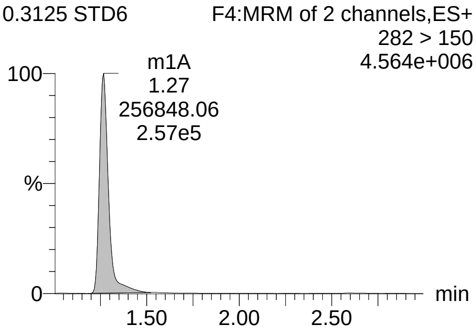

Gm

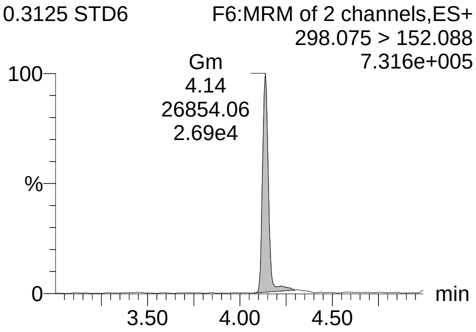

mcm5U

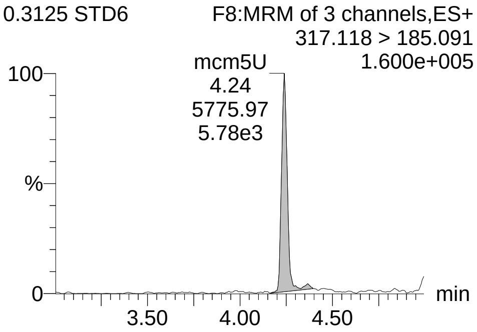

mcm5s2U

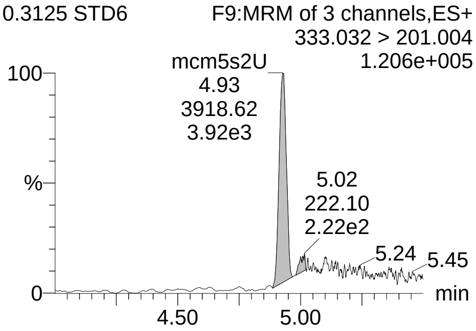

G13C15N

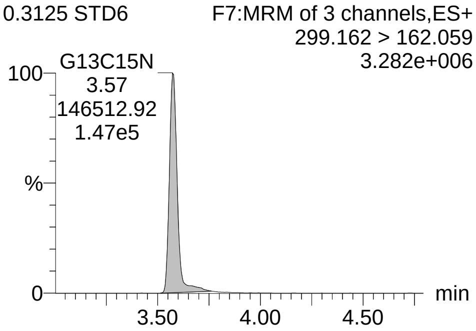

#	Name	Trace	RT	Area	IS Area	Response	Primar...	Conc.	%Dev
1	m1A	282 > 150	1.27	256848.063	146512.922	1.753	bb	0.3	-7.3
2	Gm	298.075 > 152.088	4.14	26854.059	146512.922	0.183	bb	0.3	-3.7
3	mcm5U	317.118 > 185.091	4.24	5775.967	146512.922	0.039	bb	0.3	-0.9
4	mcm5s2U	333.032 > 201.004	4.93	3918.624	146512.922	0.027	bb	0.3	-0.9
5	G13C15N	299.162 > 162.059	3.57	146512.922		146512.922	bb	1.1	7.6

Dataset: L:\masslynx\2020DS.PRO\JH20210215R.qld

Last Altered: Monday, February 15, 2021 09:49:11 Eastern Standard Time
Printed: Monday, February 15, 2021 09:51:12 Eastern Standard Time

Name: JH20210214SSTD7, Date: 14-Feb-2021, Time: 17:32:58, ID: STD7, Description: 0.15625

m1A

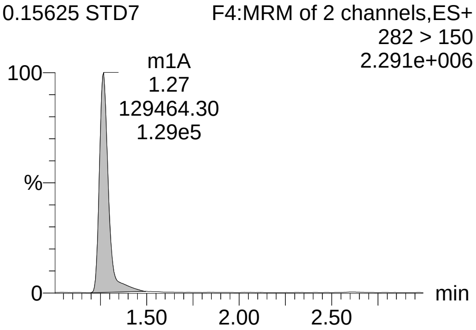

Gm

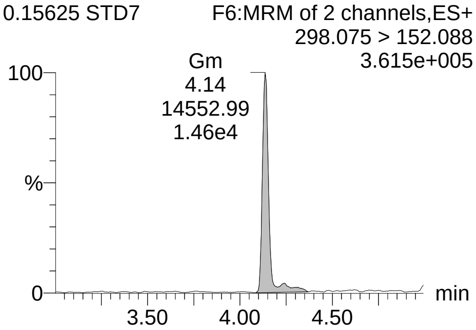

mcm5U

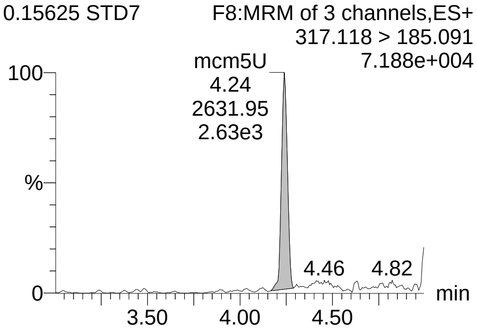

mcm5s2U

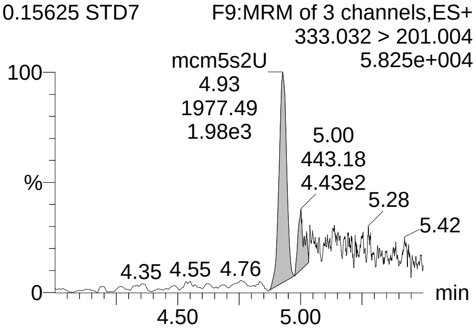

G13C15N

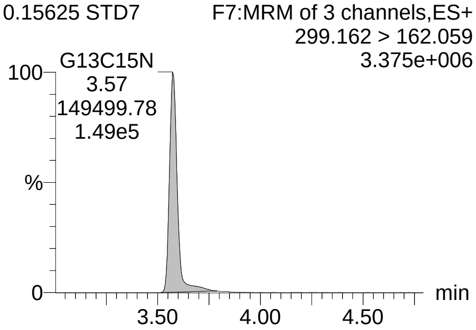

#	Name	Trace	RT	Area	IS Area	Response	Primar...	Conc.	%Dev
1	m1A	282 > 150	1.27	129464.305	149499.781	0.866	bb	0.1	-34.8
2	Gm	298.075 > 152.088	4.14	14552.990	149499.781	0.097	bb	0.2	6.4
3	mcm5U	317.118 > 185.091	4.24	2631.953	149499.781	0.018	bb	0.2	10.9
4	mcm5s2U	333.032 > 201.004	4.93	1977.488	149499.781	0.013	bb	0.2	21.4
5	G13C15N	299.162 > 162.059	3.57	149499.781		149499.781	bb	1.1	9.8

Name: JH20210214SSTD8, Date: 14-Feb-2021, Time: 17:40:47, ID: STD8, Description: 0.078125

m1A

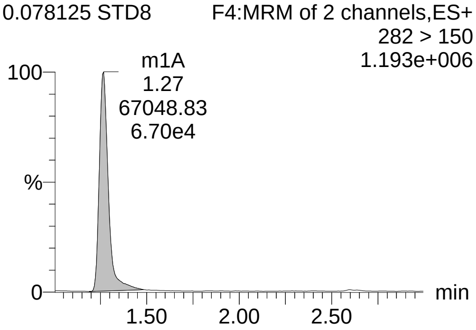

Gm

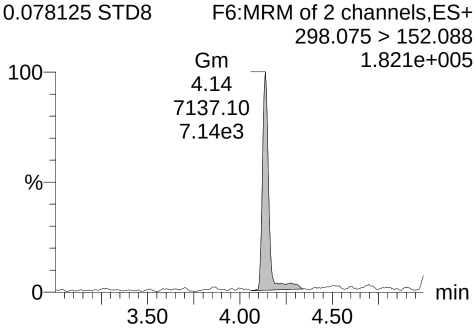

mcm5U

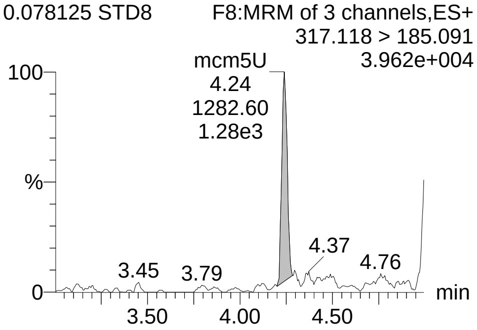

Dataset: L:\masslynx\2020DS.PRO\JH20210215R.qld

Last Altered: Monday, February 15, 2021 09:49:11 Eastern Standard Time
Printed: Monday, February 15, 2021 09:51:12 Eastern Standard Time

Name: JH20210214SSTD8, Date: 14-Feb-2021, Time: 17:40:47, ID: STD8, Description: 0.078125

mcm5s2U

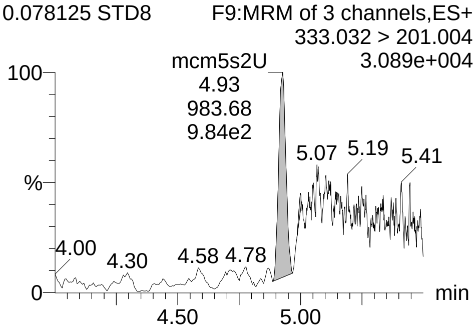

G13C15N

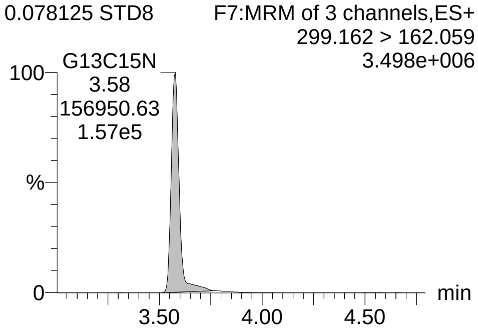

#	Name	Trace	RT	Area	IS Area	Response	Primar...	Conc.	%Dev
1	m1A	282 > 150	1.27	67048.828	156950.625	0.427	bbX	0.0	-88.5
2	Gm	298.075 > 152.088	4.14	7137.099	156950.625	0.045	bb	0.1	8.5
3	mcm5U	317.118 > 185.091	4.24	1282.597	156950.625	0.008	bbX	0.1	46.3
4	mcm5s2U	333.032 > 201.004	4.93	983.679	156950.625	0.006	bbX	0.1	63.5
5	G13C15N	299.162 > 162.059	3.58	156950.625		156950.625	bb	1.2	15.3

Name: JH20210214Blank3, Date: 14-Feb-2021, Time: 17:48:35, ID: blk, Description: blank

m1A

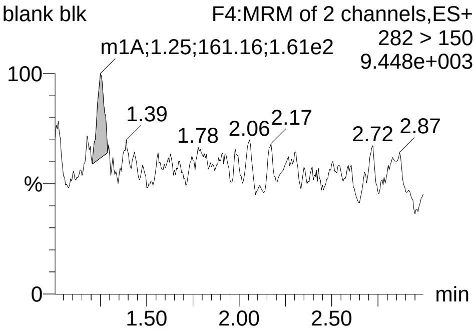

Gm

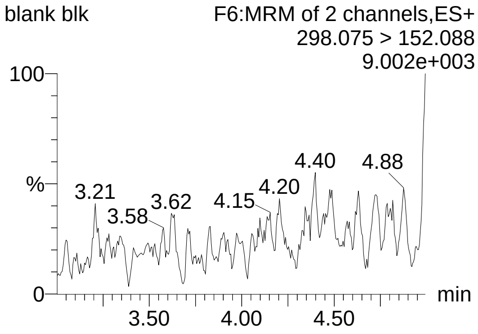

mcm5U

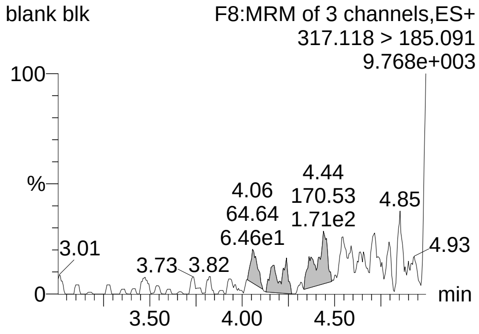

mcm5s2U

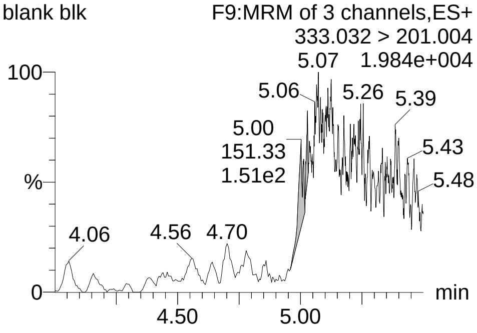

G13C15N

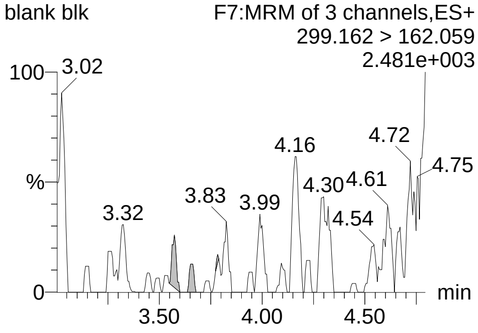

#	Name	Trace	RT	Area	IS Area	Response	Primar...	Conc.	%Dev
1	m1A	282 > 150	1.25	161.162	14.561	11.068	bb	2.3	
2	Gm	298.075 > 152.088			14.561				
3	mcm5U	317.118 > 185.091	4.24	105.661	14.561	7.256	bb	45.4	
4	mcm5s2U	333.032 > 201.004			14.561				
5	G13C15N	299.162 > 162.059	3.57	14.561		14.561	bb	0.0	-100.0
